# Physical activity and molecular subtypes of colorectal cancer: a pooled observational analysis and Mendelian randomization study

**DOI:** 10.1093/jncics/pkaf095

**Published:** 2025-10-01

**Authors:** Christos V Chalitsios, Georgios Markozannes, Elom K Aglago, Sonja I Berndt, Daniel D Buchanan, Peter T Campbell, Yin Cao, Andrew T Chan, Niki Dimou, David A Drew, Amy J French, Peter Georgeson, Marios Giannakis, Stephen B Gruber, Marc J Gunter, Tabitha A Harrison, Michael Hoffmeister, Li Hsu, Wen-Yi Huang, Meredith A J Hullar, Jeroen R Huyghe, Brigid M Lynch, Victor Moreno, Neil Murphy, Christina C Newton, Jonathan A Nowak, Mireia Obón-Santacana, Shuji Ogino, Conghui Qu, Stephanie L Schmit, Robert S Steinfelder, Wei Sun, Claire E Thomas, Amanda E Toland, Quang M Trinh, Tomotaka Ugai, Caroline Y Um, Bethany Van Guelpen, Syed H Zaidi, Robert E Schoen, Michael O Woods, Hermann Brenner, Laura Andreson, Andrew J Pellatt, Ulrike Peters, Amanda I Phipps, Konstantinos K Tsilidis

**Affiliations:** Department of Hygiene and Epidemiology, University of Ioannina, Ioannina, Greece; Department of Hygiene and Epidemiology, University of Ioannina, Ioannina, Greece; Department of Epidemiology and Biostatistics, School of Public Health, Imperial College London, London, United Kingdom; Department of Epidemiology and Biostatistics, School of Public Health, Imperial College London, London, United Kingdom; Division of Cancer Epidemiology and Genetics, National Cancer Institute, National Institutes of Health, Bethesda, MD, United States; Colorectal Oncogenomics Group, Department of Clinical Pathology, Melbourne Medical School, The University of Melbourne, Parkville, Australia; University of Melbourne Centre for Cancer Research, The University of Melbourne, Parkville, Australia; Genomic Medicine and Family Cancer Clinic, The Royal Melbourne Hospital, Parkville, Victoria, Australia; Department of Epidemiology and Population Health, Albert Einstein College of Medicine, Bronx, NY, United States; Division of Public Health Sciences, Department of Surgery, Washington University School of Medicine, St Louis, MO, United States; Alvin J. Siteman Cancer Center at Barnes-Jewish Hospital and Washington University School of Medicine, St Louis, MO, United States; Division of Gastroenterology, Department of Medicine, Washington University School of Medicine, St Louis, MO, United States; Division of Gastroenterology, Massachusetts General Hospital and Harvard Medical School, Boston, MA, United States; Channing Division of Network Medicine, Brigham and Women’s Hospital and Harvard Medical School, Boston, MA, United States; Clinical and Translational Epidemiology Unit, Massachusetts General Hospital and Harvard Medical School, Boston, MA, United States; Broad Institute of Harvard and MIT, Cambridge, MA, United States; Department of Epidemiology, Harvard T.H. Chan School of Public Health, Harvard University, Boston, MA, United States; Department of Immunology and Infectious Diseases, Harvard T.H. Chan School of Public Health, Harvard University, Boston, MA, United States; Nutrition and Metabolism Branch, International Agency for Research on Cancer, Lyon, France; Division of Gastroenterology, Massachusetts General Hospital and Harvard Medical School, Boston, MA, United States; Clinical and Translational Epidemiology Unit, Massachusetts General Hospital and Harvard Medical School, Boston, MA, United States; Division of Laboratory Genetics, Department of Laboratory Medicine and Pathology, Mayo Clinic, Rochester, MN, United States; Colorectal Oncogenomics Group, Department of Clinical Pathology, Melbourne Medical School, The University of Melbourne, Parkville, Australia; University of Melbourne Centre for Cancer Research, The University of Melbourne, Parkville, Australia; Department of Medical Oncology, Dana-Farber Cancer Institute, Boston, MA, United States; Broad Institute of MIT and Harvard, Cambridge, MA, United States; Department of Medicine, Brigham and Women’s Hospital, Harvard Medical School, Boston, MA, United States; Department of Medical Oncology and Therapeutics Research and Center for Precision Medicine, City of Hope National Medical Center, Duarte, CA, United States; Department of Epidemiology and Biostatistics, School of Public Health, Imperial College London, London, United Kingdom; Nutrition and Metabolism Branch, International Agency for Research on Cancer, Lyon, France; Public Health Sciences Division, Seattle, WA, United States; Division of Clinical Epidemiology and Aging Research, German Cancer Research Center (Deutsches Krebsforschungszentrum, DKFZ), Heidelberg, Germany; Public Health Sciences Division, Seattle, WA, United States; Department of Biostatistics, University of Washington, Seattle, WA, United States; Division of Cancer Epidemiology and Genetics, National Cancer Institute, National Institutes of Health, Bethesda, MD, United States; Public Health Sciences Division, Seattle, WA, United States; Public Health Sciences Division, Seattle, WA, United States; Centre for Epidemiology and Biostatistics, Melbourne School of Population and Global Health, The University of Melbourne, Victoria, Australia; Cancer Epidemiology Division, Cancer Council Victoria, Melbourne, Victoria, Australia; Unit of Biomarkers and Susceptibility, Oncology Data Analytics Program, Catalan Institute of Oncology, L’Hospitalet del Llobregat, Barcelona, Spain; ONCOBELL Program, Bellvitge Biomedical Research Institute (IDIBELL), L’Hospitalet de Llobregat, Barcelona, Spain; Consortium for Biomedical Research in Epidemiology and Public Health, Madrid, Spain; Department of Clinical Sciences, Faculty of Medicine and health Sciences and Universitat de Barcelona Institute of Complex Systems, University of Barcelona, L’Hospitalet de Llobregat, Barcelona, Spain; Nutrition and Metabolism Branch, International Agency for Research on Cancer, Lyon, France; Department of Population Science, American Cancer Society, Atlanta, GA, United States; Program in MPE Molecular Pathological Epidemiology, Department of Pathology, Brigham and Women’s Hospital, Harvard Medical School, Boston, MA, United States; Unit of Biomarkers and Susceptibility, Oncology Data Analytics Program, Catalan Institute of Oncology, L’Hospitalet del Llobregat, Barcelona, Spain; ONCOBELL Program, Bellvitge Biomedical Research Institute (IDIBELL), L’Hospitalet de Llobregat, Barcelona, Spain; Consortium for Biomedical Research in Epidemiology and Public Health, Madrid, Spain; Department of Clinical Sciences, Faculty of Medicine and health Sciences and Universitat de Barcelona Institute of Complex Systems, University of Barcelona, L’Hospitalet de Llobregat, Barcelona, Spain; Department of Epidemiology, Harvard T.H. Chan School of Public Health, Harvard University, Boston, MA, United States; Broad Institute of MIT and Harvard, Cambridge, MA, United States; Program in MPE Molecular Pathological Epidemiology, Department of Pathology, Brigham and Women’s Hospital, Harvard Medical School, Boston, MA, United States; Tokyo Medical and Dental University (Institute of Science Tokyo), Tokyo, Japan; Public Health Sciences Division, Seattle, WA, United States; Genomic Medicine Institute, Cleveland Clinic, Cleveland, OH, United States; Population and Cancer Prevention Program, Case Comprehensive Cancer Center, Cleveland, OH, United States; Public Health Sciences Division, Seattle, WA, United States; Public Health Sciences Division, Seattle, WA, United States; Public Health Sciences Division, Seattle, WA, United States; Departments of Cancer Biology and Genetics, The Ohio State University, Comprehensive Cancer Center, College of Medicine, The Ohio State University Wexner Medical Center, Columbus, OH, United States; Ontario Institute for Cancer Research, Toronto, Ontario, Canada; Department of Epidemiology, Harvard T.H. Chan School of Public Health, Harvard University, Boston, MA, United States; Department of Population Science, American Cancer Society, Atlanta, GA, United States; Department of Diagnostics and Intervention, Oncology Unit, Umeå University, Umeå, Sweden; Wallenberg Centre for Molecular Medicine, Umeå University, Umeå, Sweden; Ontario Institute for Cancer Research, Toronto, Ontario, Canada; Department of Medicine and Epidemiology, University of Pittsburgh Medical Center, Pittsburgh, PA, United States; Discipline of Genetics, Memorial University of Newfoundland, St John’s, Canada; Division of Clinical Epidemiology and Aging Research, German Cancer Research Center (Deutsches Krebsforschungszentrum, DKFZ), Heidelberg, Germany; German Cancer Consortium (Deutsches Konsortium für Translationale Krebsforschung, DKTK), German Cancer Research Center (Deutsches Krebsforschungszentrum, DKFZ), Heidelberg, Germany; Health Research Methods, Evidence and Impact, McMaster University, Hamilton, Ontario, Canada; Intermountain Health, Salt Lake City, UT, United States; Public Health Sciences Division, Seattle, WA, United States; Department of Epidemiology, University of Washington School of Public Health, Seattle, WA, United States; Public Health Sciences Division, Seattle, WA, United States; Department of Epidemiology, University of Washington School of Public Health, Seattle, WA, United States; Department of Hygiene and Epidemiology, University of Ioannina, Ioannina, Greece; Department of Epidemiology and Biostatistics, School of Public Health, Imperial College London, London, United Kingdom

## Abstract

**Background:**

Physical activity is associated with lower colorectal cancer (CRC) risk, but its association with molecular subtypes defined by genetic and epigenetic alterations of the disease is unclear. Such information may enhance the understanding of the mechanisms related to the benefits of physical activity.

**Methods:**

Pooled observational (cases: n = 5386; controls: n = 6798; studies n = 5) and genome-wide association data (cases: n = 8178; controls: n = 10 472; studies n = 5) were used. We used multivariable logistic regression models and Mendelian randomization to assess the association between physical activity and the risk of CRC subtypes defined by individual tumor markers (and marker combinations), namely microsatellite instability status, CpG island methylator phenotype status, and *BRAF* and *KRAS* mutations. We used case-only analysis to test for differences between molecular subtypes. We applied Bonferroni correction to account for multiple tests.

**Results:**

In the pooled observational analysis, higher levels of physical activity were associated with lower CRC risk (Obs-per 1SD, odds ratio [OR] = 0.94, 95% confidence interval [CI] = 0.90 to 0.97), with an association that was stronger in males (Obs-per 1SD, OR = 0.91, 95% CI = 0.87 to 0.96) than in females (Obs-per 1SD, OR = 0.97, 95% CI = 0.91 to 1.03; *P*_interaction_ = .04). Higher physical activity was associated with a lower risk of CRC across all molecular subtypes, especially in males. There was no difference in the associations by subtypes by pooled observational or Mendelian randomization analyses. The findings did not differ by study design, anatomical site, and early or late age onset of CRC.

**Conclusions:**

Our findings suggest that physical activity is not differentially associated with the 4 major molecular subtypes involved in colorectal carcinogenesis, indicating that its benefits extend broadly across colorectal cancer pathogenesis.

## Introduction

An important element of colorectal cancer (CRC) primary prevention involves identifying modifiable lifestyle factors that influence the risk of developing the disease. Such factors associated with CRC development include smoking,^1^ anthropometric measures (eg, body mass index [BMI],^2^^,^^3^ waist circumference^2^) alcohol intake,^4^ diet (eg, wholegrains, fiber, dairy products, processed and red meat),^5^ and physical activity.^6-8^ Physical activity may reduce CRC risk through lower circulating insulin^9^^,^^10^ and pro-inflammatory factors,^11^^,^^12^ enhanced immune response,^11^^,^^13^ and beneficial changes to the gut microbiome.^14^^,^^15^

CRC is one of the most common cancers and is responsible for a large burden of cancer death.^16^ It is characterized by substantial genetic and epigenetic diversity.^17^ Detailed molecular characterization of CRC using clinically relevant genetic and epigenetic markers shows great potential for improving prognosis^18^ and guiding treatment decisions.^19^ Mutations in the *KRAS* gene play a crucial role in developing colorectal adenomas, occurring in approximately 30%-40% of sporadic cases.^20^ Additionally, microsatellite instability, identified by frequent changes in repetitive DNA sequences, is present in approximately 15% of CRC cases and is associated with a more favorable prognosis.^21^ Some microsatellite instability–high CRC cases also feature the CpG island methylator phenotype and *BRAF* c.1799T>A (p.V600E) mutations.^7^  *BRAF* p. V600E is associated with a worse prognosis,^22^ whereas BRAF mutations predict response to encorafenib plus cetuximab,^23^^,^^24^  *KRAS* mutations indicate poor response to estimated glomerular filtration rate inhibitors,^25^ and microsatellite instability–high status predicts response to immunotherapy.^26^^,^^27^ Currently, there is no evidence of whether the association of physical activity with CRC varies by tumor molecular markers; however, such information might enhance the understanding of the mechanisms related to the benefits of physical activity.

This study aimed to illuminate the association between physical activity and the risk of CRC by tumor molecular subtypes (microsatellite instability, CpG island methylator phenotype, *BRAF*, and *KRAS* mutation status). Thus, we performed an observational and a 2-sample Mendelian randomization analysis using data from the Genetics and Epidemiology of Colorectal Cancer Consortium (GECCO) and the Colon Cancer Family Registry (CCFR).

## Methods

### Observational analysis

#### Study population

This study sample included CRC cases and controls nested in 3 cohorts (Cancer Prevention Study-II [CPS-II],^28^^,^^29^ Nurses’ Health Study [NHS],^30^ and Health Professionals Follow-up Study [HPFS]) and 2 case-control studies (Darmkrebs: Chancen der Verhütung durch Screening Study [DACHS]^31^^,^^32^ and Diet, Activity and Lifestyle Study [DALS])^33^^,^^34^ within GECCO with available tumor marker and physical activity data (Table S1). All CRC cases were defined as colorectal adenocarcinoma and confirmed by pathological records, medical records, and/or death certificate information. Additional information on the contributing studies is included in the Supplements (Table S1 and Description of included studies). All participants provided written informed consent. A research ethics committee or institutional review board approved each study.

#### Tumor molecular subtypes of CRC

Testing for microsatellite instability, CpG island methylator phenotype, and mutations in the *BRAF* and *KRAS* genes was conducted previously by each study and according to individual study protocols. Briefly, microsatellite instability testing was primarily conducted using polymerase chain reaction following accepted guidelines (CPS-II, HPFS, NHS)^35^ with 4 or more interpretable markers typically required to classify tumors (Table S2). DALS and DACHS used a mononucleotide panel of 2 and 3 markers, respectively.

The studies used polymerase chain reaction, sequencing, and immunohistochemistry techniques to assess *BRAF* and *KRAS* mutations, as detailed in the Supplements (harmonization of colorectal tumor marker data). CpG island methylator phenotype status was determined using methylation analyses (Table S3). The CPS-II, HPFS, and NHS used MethyLight to determine CpG island methylator phenotype status. CPS-II, HPFS, and NHS used an 8-gene panel.^36^ CpG island methylator phenotype categories for our analyses were CpG island methylator phenotype high and CpG island methylator phenotype low/negative. DACHS determined CpG island methylator phenotype status using a different 5-gene panel,^36^ and DALS determined CpG island methylator phenotype status using a classic panel of CpG islands.^37^^,^^38^ Additionally, we combined markers to create subtype classifications: subtypes 1-5 were created according to the Jass classification,^39^ and types 6-16 were numbered consecutively by the status of microsatellite instability, CpG island methylator phenotype, *BRAF*, and *KRAS*.

#### Physical activity definition

In cohort studies, data on physical activity were collected at baseline through in-person interviews or structured self-administered questionnaires. In case-control studies, this information was obtained from cases and controls, referencing the period 1-2 years before enrollment. Dietary variables were ascertained using food frequency questionnaires. The exposure of interest was physical activity, defined as the sum of leisure time and undifferentiated activity, expressed as the metabolic equivalent of task (MET) hours per week. As the range of MET hours per week was different among the studies, and to make the effect estimates comparable, MET hours per week were standardized by subtracting the mean (CPS-II = 16, HPFS = 33, NHS = 17, DACHS = 52, DALS = 38) and dividing it by the SD (CPS-II = 11, HPFS = 28, NHS = 16, DACHS = 33, DALS = 18) (Figure S1).

#### Confounders

In cohort studies (CPS-II, HPFS, and NHS), sociodemographic and lifestyle information was collected at baseline through in-person interviews or structured self-administered questionnaires. In case-control studies (DACHS and DALS), this information was gathered from cases and controls with reference to the period 1-2 years before enrollment. Dietary variables were ascertained using food frequency questionnaires. A multistep iterative data-harmonization procedure was applied, reconciling each study’s unique protocols and data collection instruments. Multiple quality-control checks were performed, and outlying values of variables were truncated to the minimum or maximum value of an established range for each variable. Variables were combined into a single dataset with standard definitions, standardized coding, and standardized permissible values.

#### Statistical analysis

Physical activity was modeled continuously (per 1-SD) and categorically in tertiles defined among controls only. Multinomial logistic regression models were used to estimate the relative risk ratio for the association between physical activity and CRC subtypes (compared with control participants) defined by tumor markers (microsatellite instability high vs microsatellite stable or microsatellite instability low, CpG island methylator phenotype high vs low/negative, and *BRAF* or *KRAS* mutated vs wild type). Logistic regression models were performed to assess if the differences in the associations of physical activity with CRC across molecularly defined subtypes were statistically significant among cases only. In the case-only analysis, we also conducted a logistic regression analysis defining Jass type 4 (microsatellite stable or microsatellite instability low, CpG island methylator phenotype low/negative, *BRAF* wild type, *KRAS* mutated) as the reference group to evaluate significance of differences among Jass types of tumor marker combinations. Jass types with at least 50 cases were assessed in relation to physical activity. The multivariable models included study population, age (continuous, years), sex (male, female), smoking status (never, former, current smokers), alcohol consumption (continuous, grams per day), education (less than high school graduate, high school graduate, some college, college graduate), and red meat intake (continuous, servings per day).

The analysis included only participants with complete information. Separate analyses were conducted by study design (cohort and case-control studies), sex, cancer anatomical site (proximal colon, distal colon, colon, rectum), and by early (aged 50 years and younger) and later (older than 50 years) age onset CRC. A sensitivity analysis was conducted after imputing missing values for physical activity and all covariates used in the adjustment model (see Table 1) using multiple imputations by chained equations^40^ with 5 imputed datasets and 5 iterations using the random forest method. Diagnostic plots suggested a good performance of this method (Figure S2-S4). As body size and adiposity is potentially on the causal pathway linking physical activity with colorectal cancer, we did not include BMI in the main analyses. However, we additionally adjusted our main model for BMI, processed meat, fiber, fruits, and vegetables without observing any difference in the results. Finally, we pooled study-specific odds ratios (ORs) for the associations between physical activity and overall CRC using random effects meta-analysis models to evaluate for heterogeneity between studies.

**Table 1.** Baseline characteristics of cases and controls used in observational analysis

Characteristics	Cases, No. (%) (n = 5386)	Controls, No. (%) (n = 6798)
Study		
CPS-II	860 (16)	1003 (14.8)
DACHS	2009 (37.3)	2789 (41)
DALS	1095 (20.3)	1162 (17.1)
HPFS	629 (11.7)	602 (8.9)
NHS	793 (14.7)	1242 (18.3)
Age,^a^ mean (SD), y	69 (10)	69 (10)
Unknown	5 (<0.1)	0
Sex		
Male	2834 (52.6)	3430 (50.5)
Female	2552 (47.4)	3368 (49.5)
Smoking status		
Never smoker	2290 (42.5)	3217 (47.3)
Former smoker	2389 (44.4)	2869 (42.2)
Current smoker	634 (11.8)	661 (9.7)
Unknown	73 (1.4)	51 (0.8)
Body mass index, kg/m^2^		
Mean (SD)	27 (4.6)	26.1 (4.2)
Underweight, <18.5	48 (0.9)	74 (1.1)
Normal, 18.5 to <24.9	1824 (33.9)	2795 (41.1)
Overweight, 25- 30	2276 (42.3)	2813 (41.4)
Obese, >30	1088 (20.2)	1001 (14.7)
Unknown	228 (3.8)	115 (1.7)
Dietary intake, mean (SD)		
Red meat, servings per day	0.85 (0.61)	0.77 (0.55)
Unknown	141 (2.6)	167 (2.5)
Processed meat, servings per day	0.48 (0.46)	0.45 (0.43)
Unknown	279 (5.2)	253 (3.7)
Fruits, servings per day	1.64 (1.44)	1.67 (1.36)
Unknown	230 (4.3)	316 (4.6)
Vegetables, servings per day	2.47 (1.91)	2.41 (1.85)
Unknown	225 (4.2)	312 (4.6)
Fiber, gm per day	21 (9)	21 (9)
Unknown	2260 (42)	2987 (44)
Education level		
Less than high school graduate	627 (11.6)	598 (8.8)
High school graduate or completed General Education Development test	1592 (29.6)	1984 (29.2)
Some college or technical school	1162 (21.6)	1194 (17.6)
College graduate	1882 (34.9)	2980 (43.8)
Unknown	123 (2.3)	42 (0.6)
First-degree relative with CRC	565 (10.3)	483 (7.1)
Unknown	390 (7.2)	368 (5.4)
Location of CRC		
Distal colon	1722 (32)	—
Proximal colon	2373 (44.1)	—
Rectum, including rectosigmoid junction	1230 (22.8)	—
Unknown	61 (1.1)	—
CRC stage		
Stage 1 or local	1438 (26.7)	
Stage 2-3 or regional	3146 (58.4)	
Stage 4 or distant	601 (11.2)	
Unknown	201 (3.7)	
*BRAF*		
Wild type	4262 (79.2)	—
Mutated	543 (10.1)	—
Unknown	577 (10.7)	—
*KRAS*		
Wild type	3162 (58.7)	—
Mutated	1630 (30.3)	—
Unknown	594 (11)	—
MSI		
MSS/MSI low	4221 (78.4)	—
MSI high	705 (13.1)	—
Unknown	460 (8.5)	—
CIMP		
Low/negative	4015 (74.5)	—
High	926 (17.2)	—
Unknown	445 (8.3)	—
Jass type (subtypes combinations)		
[1] MSI-high, CIMP+, *BRAF* mut, *KRAS* wt	260 (4.8)	—
[2] MSS or MSI-low, CIMP+, *BRAF* mut, *KRAS* wt	103 (1.9)	—
[3] MSS or MSI-low, CIMP-, *BRAF* wt, *KRAS* mut	1161 (21.6)	—
[4] MSS or MSI-low, CIMP-, *BRAF* wt, *KRAS* wt	1868 (34.7)	—
[5] MSI-high, CIMP-, *BRAF* wt, *KRAS* wt	98 (1.8)	—
[6] MSS or MSI-low, CIMP-, *BRAF* mut, *KRAS* wt	77 (1.4)	—
[7] MSS or MSI-low, CIMP-, *BRAF* mut, *KRAS* mut	5 (0.1)	—
[8] MSS or MSI-low, CIMP+, *BRAF* wt, *KRAS* wt	115 (2.1)	—
[9] MSS or MSI-low, CIMP+, *BRAF* wt, *KRAS* mut	147 (2.7)	—
[10] MSS or MSI-low, CIMP+, *BRAF* mut, *KRAS* mut	—	—
[11] MSI-high, CIMP-, *BRAF* wt, *KRAS* mut	59 (1.1)	—
[12] MSI-high, CIMP-, *BRAF* mut, *KRAS* wt	13 (0.2)	—
[13] MSI-high, CIMP-, *BRAF* mut, *KRAS* mut	2 (0)	—
[14] MSI-high, CIMP+, *BRAF* wt, *KRAS* wt	108 (2)	—
[15] MSI-high, CIMP+, *BRAF* wt, *KRAS* mut	21 (0.4)	—
[16] MSI-high, CIMP+, *BRAF* mut, *KRAS* mut	9 (0.2)	—
Unknown	1913 (32.1)	—

Abbreviations: CIMP = CpG island methylator phenotype; CIMP- = CIMP low/negative; CIMP+ = CIMP high; CPSII = Cancer Prevention Study II; CRC = colorectal cancer; DACHS = Darmkrebs: Chancen der Verhctung durch Screening Study; DALS = Activity and Lifestyle Study; HPFS = Health Professionals Follow-up Study; MSI = microsatellite instability; MSS = microsatellite stable; mut = mutated; NHS = Nurses’ Health Study; wt = wild type.

Figures are expressed as No (%) unless otherwise specified. The em dashes mean that there are not available data as they are relevant only to colorectal cancer cases and not for those without (controls).

a Age at diagnosis (cases) and selection (controls).

We used a Bonferroni-corrected threshold of 0.004 (0.05/12; 4 subtype classifications based on 4 individual tumor markers being tested × 3 analyses: men only, women only, sex combined) to assess statistical significance for the case-control and case-only analyses of the primary subtypes (microsatellite instability, CpG island methylator phenotype–status *BRAF*, and *KRAS*). For secondary analyses (marginal analysis for overall CRC and subsequent subgroup analyses), we considered a 2-sided *P* value less than .05 as statistically significant. Associations with *P* values between the Bonferroni-corrected and nominal threshold (.05) were characterized as suggestive. Analyses were performed using R v4.3.1 (R Foundation for Statistical Computing, Vienna, Austria).

### Mendelian randomization

#### Study population

For overall CRC, summary statistics were drawn from a genome-wide association study (GWAS) meta-analysis of 17 studies, encompassing 78 473 CRC cancer cases and 107 143 controls of European descent.^41^ Summary data for CRC molecular subtypes were drawn from a GWAS meta-analysis of 10 studies (CCFR,^42^ CPSII,^28^^,^^29^ NHS,^30^ HPFS, DACHS,^31^^,^^32^ DALS,^33^^,^^34^ Early Detection Research Network,^43^ European Prospective Investigation into Cancer Sweden,^44^ Melbourne Collaborative Cohort Study,^45^ and Northern Sweden Health and Disease Study^46^) within the CCFR and the GECCO consortia (Table S1). The current study includes 8178 CRC cases and 10 472 controls of European ancestry, with available information on the 4 molecular markers (Tables S4 and S5). Additional information on contributing studies is included in Table S3 and the Supplements (Table S1 and description of included studies).

#### Physical activity definition

Summary statistics for accelerometer-based average physical activity were obtained from a GWAS of 91 105 UK Biobank participants that explained approximately 0.2% of the variance in physical activity.^47^ Physical activity was objectively measured using a wrist-worn accelerometer worn continuously for 7 days. Activity levels were recorded in milligravity units (mg), with a mean of 29.0 (8.14 mg). One standard deviation corresponds to approximately 50 minutes of moderate-intensity activity (eg, brisk walking) per week.

#### Statistical analysis

Mendelian randomization leverages genetic variants, typically single-nucleotide polymorphisms, as instrumental variables to estimate the causal effect of an exposure on an outcome.^48^^,^^49^ Because genetic variants are randomly assorted at meiosis and fixed at conception, Mendelian randomization is less susceptible to confounding and reverse causation than observational studies. For Mendelian randomization estimates to be valid, 3 core assumptions must hold: (1) the genetic variants should be associated with the exposure, (2) they should not be associated with confounders of the exposure-outcome association, and (3) they should influence the outcome solely through the exposure and not via alternative biological pathways. A violation of the third assumption—known as horizontal pleiotropy—can bias results.

To create the genetic instruments, all relevant genetic variants were selected if they demonstrated genome-wide significant association (*P* < 5 × $10^{-8})\;$to represent genetic susceptibility to physical activity and were uncorrelated ($r^{2}$ < 0.001) within a 10-Mb window based on the European 1000 genomes reference panel. F-statistics of at least 10 were considered of adequate instrument strength.

To estimate study power, we considered that the genetic instruments explained 0.2% of the variance in physical activity; given the sample size for overall CRC, the minimum detectable odds ratio with 80% power at a 5% significance level was 0.74. The primary method was random-effects inverse variance–weighted Mendelian randomization.^50^ To account for potential horizontal pleiotropy, several Mendelian randomization sensitivity analyses (Mendelian randomization–Egger,^51^ weighted median,^52^ weighted mode^53^) were performed, each providing a valid Mendelian randomization estimate under different combinations of assumptions. We additionally implemented the Mendelian randomization pleiotropy residual sum and outlier test (Mendelian randomization–Pleiotropy RESidual Sum and Outlie [PRESSO]) to detect and exclude potential outlying genetic variants.^54^ Mendelian randomization analysis was performed with R v4.1.2 using the TwoSampleMR package.

We applied a Bonferroni-corrected significance threshold of a *P* value less than .012 (0.05/4), accounting for the 4 primary tumor markers evaluated (Mendelian randomization status, CpG island methylator phenotype status, *BRAF*, and *KRAS* mutations), in the case-control and case-only analyses. For the secondary analysis assessing overall CRC risk, a 2-sided *P* value less than .05 was considered statistically significant. Associations with *P* values between the Bonferroni-corrected and nominal threshold (0.05) were characterized as suggestive.

## Results

### Observational analysis

#### Study population characteristics

The study sample comprised 5386 CRC cases and 6798 controls from 5 observational studies (Table 1). Compared with controls, individuals with a CRC diagnosis were more likely to be male than females (52.6% vs 50.5%), former or current smokers (56.2% vs 51.9%), and obese (20.2% vs 14.7%) and to have a first-degree relative with CRC (10.3% vs 7.1%). Among cases, 13.1% were microsatellite instability high (*n* = 705), 17.2% were CpG island methylator phenotype high (*n* = 926), 10.1% were *BRAF*-mutated (n = 543), and 30.3% were *KRAS* mutated (*n* = 1630), with the type 4 (34.7%) being the most frequent Jass type.

#### Physical activity and overall CRC risk

Higher MET hours per week of physical activity per 1 standard deviation (across all studies approximately 30.1 MET hours per week) were associated with lower odds of developing CRC (Obs-per 1SD, OR = 0.94, 95% confidence interval [CI] = 0.90 to 0.97), with a stronger association observed in males (Obs-per 1SD, OR = 0.91, 95% CI = 0.87 to 0.96), and no association observed in females (Obs-per 1SD, OR = 0.97, 95% CI = 0.92 to 1.03) (*P*_interaction_ = .04) (Table 2). A similar association between physical activity and overall CRC was found when individual study odds ratios were pooled in a meta-analysis with a low between-study heterogeneity (*I*^2^ = 27.4%, *P*_Cochran_  _*Q*_ = .24, τ^2^ < 0.01) (Figure S5). In the subgroup analysis by study design, the association between physical activity and overall CRC risk was present only in the cohort (Obs-per 1SD, OR = 0.89, 95% CI = 0.84 to 0.95) and not in case-control studies (Obs-per 1SD, OR = 0.97, 95% CI = 0.92 to 1.02; *P*_heterogeneity_ = .0768) (Table S6). When analyzed by anatomical site, physical activity was associated with lower odds of rectal (cohorts: Obs-per 1SD, OR = 0.80, 95% CI = 0.67 to 0.95) and colon cancer (cohorts: Obs-per 1SD, OR = 0.84, 95% CI = 0.76 to 0.93) in males (Tables S7-S14).

**Table 2.** Association between physical activity, colorectal cancer, and its molecular subtypes according to the observational analysis^a^^,^^b^^,^^c^

Standardized MET h/wk	Overall CRC OR (95% CI)	MSI		CIMP		*BRAF*		*KRAS*	
		MSS or MSI-low RR ratio (95% CI)	MSI-high RR ratio (95% CI)	CIMP-low/negative RR ratio (95% CI)	CIMP-high RR ratio (95% CI)	*BRAF*-wild type RR ratio (95% CI)	*BRAF*-mutated RR ratio (95% CI)	*KRAS*-wild type RR ratio (95% CI)	*KRAS*-mutated RR ratio (95% CI)
Sex combined									
No. cases	4989	3915	651	3720	849	3947	493	2930	1501
−1.58 to −0.591	Referent	Referent	Referent	Referent	Referent	Referent	Referent	Referent	Referent
−0.590 to 0.2	0.85 (0.78 to 0.93)	0.84 (0.77 to 0.93)	0.83 (0.68 to 1.01)	0.85 (0.77 to 0.94)	0.90 (0.75 to 1.06)	0.86 (0.78 to 0.95)	0.89 (0.71 to 1.11)	0.83 (0.75 to 0.93)	0.85 (0.74 to 0.98)
0.21 to 3.03	0.86 (0.78 to 0.94)	0.86 (0.78 to 0.95)	0.80 (0.66 to 0.98)	0.88 (0.80 to 0.98)	0.79 (0.66 to 0.95)	0.86 (0.78 to 0.95)	0.80 (0.64 to 1.01)	0.82 (0.74 to 0.92)	0.85 (0.74 to 0.97)
*P*_trend_	.0012	.0033	.0314	.0121	.0111	.0027	.0670	.0004	.0176
Per 1-SD	0.94 (0.90 to 0.97)	0.94 (0.90 to 0.98)	0.91 (0.83 to 0.99)	0.95 (0.91 to 0.99)	0.90 (0.83 to 0.97)	0.94 (0.90 to 0.98)	0.90 (0.82 to 1.00)	0.92 (0.88 to 0.97)	0.93 (0.88 to 0.99)
*P*	.0011	.0043	.0302	.0105	.0081	.0029	.0461	.0007	.0144
*P*_difference_	NA	.36		.17		.40		.75	
Male									
No. cases	2718	2206	268	2123	360	2242	180	1583	836
−1.58 to −0.591	Referent	Referent	Referent	Referent	Referent	Referent	Referent	Referent	Referent
−0.590 to 0.2	0.82 (0.73 to 0.93)	0.80 (0.70 to 0.91)	0.84 (0.63 to 1.14)	0.81 (0.71 to 0.93)	0.79 (0.61 to 1.02)	0.80 (0.70 to 0.91)	0.92 (0.65 to 1.32)	0.80 (0.69 to 0.93)	0.81 (0.67 to 0.98)
0.21 to 3.03	0.77 (0.68 to 0.88)	0.79 (0.69 to 0.91)	0.65 (0.47 to 0.89)	0.81 (0.71 to 0.93)	0.62 (0.47 to 0.82)	0.78 (0.68 to 0.89)	0.68 (0.46 to 1.00)	0.72 (0.62 to 0.84)	0.83 (0.69 to 1.01)
*P*_trend_	.0001	.0007	.0079	.0021	.0008	.0003	.0561	.0001	.0567
Per 1-SD	0.91 (0.87 to 0.96)	0.92 (0.87 to 0.98)	0.86 (0.75 to 0.98)	0.93 (0.88 to 0.98)	0.82 (0.72 to 0.92)	0.92 (0.87 to 0.97)	0.87 (0.74 to 1.02)	0.89 (0.84 to 0.95)	0.93 (0.86 to 1.00)
*P*	.0004	.0042	.0247	.0122	.0008	.0015	.0896	.0002	.0618
*P*_difference_	NA	.28		.02		.52		.30	
Female									
No. cases	2271	1709	383	1597	489	1705	313	1347	665
−1.58 to −0.591	Referent	Referent	Referent	Referent	Referent	Referent	Referent	Referent	Referent
−0.590 to 0.2	0.89 (0.78 to 1.01)	0.90 (0.78 to 1.04)	0.82 (0.64 to 1.07)	0.89 (0.77 to 1.03)	0.99 (0.79 to 1.25)	0.93 (0.81 to 1.08)	0.87 (0.66 to 1.16)	0.88 (0.75 to 1.03)	0.89 (0.73 to 1.09)
0.21 to 3.03	0.98 (0.85 to 1.12)	0.96 (0.82 to 1.11)	0.96 (0.74 to 1.26)	0.98 (0.84 to 1.14)	0.96 (0.75 to 1.22)	0.97 (0.84 to 1.13)	0.90 (0.67 to 1.21)	0.96 (0.82 to 1.14)	0.86 (0.69 to 1.06)
*P*_trend_	.6109	.5526	.6951	.7197	.7551	.6945	.4617	.5840	.1528
Per 1-SD	0.97 (0.92 to 1.03)	0.97 (0.91 to 1.03)	0.96 (0.85 to 1.05)	0.97 (0.92 to 1.03)	0.98 (0.88 to 1.09)	0.97 (0.91 to 1.04)	0.93 (0.81 to 1.06)	0.97 (0.91 to 1.04)	0.93 (0.84 to 1.02)
*P*	.3847	.3097	.4661	.3046	.6885	.4148	.2645	.4312	.1141
*P*_difference_	NA	.97		.69		.58		.38	

Abbreviations: CI = confidence interval; CIMP = CpG island methylator phenotype; CRC = colorectal cancer; MET = metabolic equivalent of task; MSI = microsatellite instability; MSS = microsatellite stable; OR = odds ratio; RR = relative risk.

a Controls are used as the referents for all effect estimates.

b Models are adjusted for the study population, age, sex (when not stratified), smoking status, alcohol consumption, education, and red meat intake.

c Case-only analysis used to calculate *P*_difference_.

#### Physical activity and molecular subtypes

Higher MET hours per week were associated with a lower risk of CRC across all molecular subtypes, especially in males. The case-only analysis showed no statistically significant difference in the association of MET hours per week with different molecular subtypes, after multiple testing correction (Table 2). The associations across all molecular subtypes did not differ by study design (Table S6), anatomical site (Tables S7-S14), and early- and later-age onset of CRC (Table S15). A reanalysis of the data with imputed missing values for physical activity and confounders yielded identical findings (Table S16).

**Figure 1.**
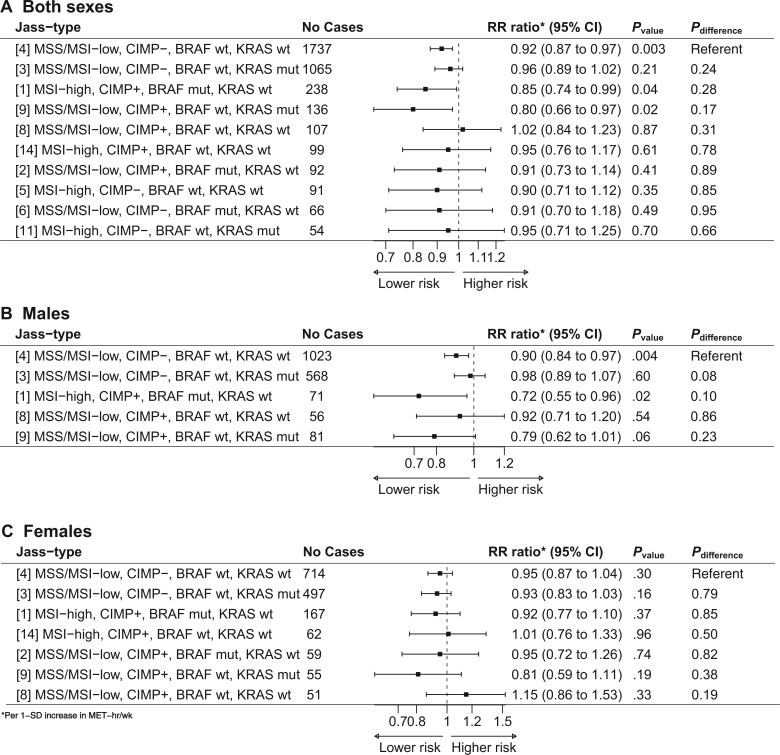
Association between physical activity (per 1-SD increase in MET hours per week) and Jass classified types of colorectal cancer, overall and stratified by sex, according to the observational analysis. Controls were used as the referent for all relative risk ratios. The models were adjusted for age, sex, study population, alcohol consumption, smoking status, education, and red meat intake. Jass types with more than 50 cases were included. *P* values were calculated using multinomial logistic regression comparing CRC cases with cancer-free controls separately for each defined Jass type. *P*_difference_ was calculated using multinomial logistic regression comparing cases of each Jass type with all additional cases not belonging to that type. Abbreviations: CI = confidence interval; CIMP = CpG island methylator phenotype; CIMP- = CIMP low/negative; CIMP+ = CIMP high; CRC = colorectal cancer; MET = metabolic equivalent of task; MSI = microsatellite instability; MSS = microsatellite stable; mut = mutated; RR = relative risk; wt = wild type.

Of 16 possible combined CRC subtypes, defined by microsatellite instability, CpG island methylator phenotype, *BRAF* and *KRAS*, 10 had 50 or more cases and were included in the combined sex analysis. There was no evidence of differences by Jass type in case-only analyses in sex-combined or sex-specific analyses (Figure 1).

### Mendelian randomization analysis

#### Physical activity and molecular subtypes

**Figure 2.**
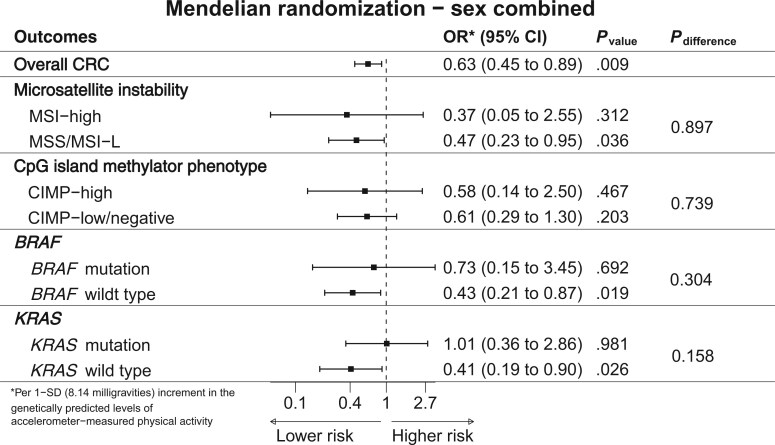
The association of genetically predicted levels of accelerometer-measured physical activity (per 1-SD [8.14 milligravities] increase) with CRC risk based on the individual tumor molecular markers, according to the Mendelian randomization analysis. *P* was calculated using the inverse variance–weighted method comparing CRC cases with cancer-free controls. *P*_difference_ was calculated using the inverse variance–weighted method comparing cases of each molecular subtype. Abbreviations: CI = confidence interval; CIMP = CpG island methylator phenotype; CRC = colorectal cancer; MET = metabolic equivalent of task; MSI = microsatellite instability; MSS = microsatellite stable; OR = odds ratio.

Six genetic variants were used as genetic instruments for physical activity, with the F-statistics ranging from 29.5 to 46.4 (Table S17). Higher genetically predicted levels (per 1-SD increase = 8.14 mg, corresponding to approximately 50 minutes of moderate activity per week) of accelerometer-measured physical activity were inversely associated with overall CRC (inverse variance–weighted OR = 0.63, 95% CI = 0.45 to 0.89) and its tumor subtypes defined by individual molecular markers, with suggestive (after correcting for multiple testing) associations (microsatellite stable or microsatellite instability low [inverse variance–weighted OR = 0.46, 95% CI = 0.23 to 0.95], *BRAF* wild type [inverse variance–weighted OR = 0.43, 95% CI = 0.21 to 0.87], and *KRAS* wild type [inverse variance–weighted OR = 0.41, 95% CI = 0.19 to 0.90]), consistent with the observational analysis (Figure 2;  Table S18). The sensitivity analyses generated similar and consistent associations (Table S18). There was no heterogeneity in the associations between genetically predicted levels of accelerometer-measured physical activity and molecular subtypes.

## Discussion

We performed an observational pooled analysis using individual-level and a 2-sample Mendelian randomization analysis using GWAS summary data to illuminate the association between physical activity and the risk of CRC by tumor molecular subtypes. Higher levels of physical activity were associated with a lower risk of CRC in males but not in females. Higher physical activity was associated with a lower risk of CRC across all molecular subtypes, especially in males, but there was no difference in the associations by subtypes, and the Mendelian randomization findings agreed. The associations across all molecular subtypes did not differ by study design, anatomical site, and early and later age onset CRC.

Our findings align with previous meta-analyses and individual cohort studies. Specifically, Johnson et al.^55^ conducted a meta-analysis across 12 cohort studies and found that each 2-unit increase in standardized physical activity score was associated with an 8% (95% CI = 0.88 to 0.96) lower CRC risk. In addition, 2 prior Mendelian randomization studies on genetically predicted higher self-reported and accelerometer-measured physical activity showed lower CRC risk.^6^^,^^56^ Another meta-analysis combining cohort and case-control studies demonstrated that individuals with the highest levels of recreational physical activity had a 20% lower risk of colon cancer (95% CI = 0.71 to 0.89) and a 13% lower risk of rectal cancer (95% CI = 0.75 to 1.01) compared with those with the lowest levels of activity.^57^ We observed that the protective association of physical activity with overall CRC risk was stronger in males than in females, consistent with previous evidence.^58^^,^^59^ This sex difference could be partly attributed to hormonal variations and differences^13^ and potential biological differences in how males and females respond to exercise.^60^ In addition, sex-specific differences in the type and intensity of physical activity may also contribute to the variation in CRC risk between males and females.^59^

CRC is a heterogeneous disease with several distinct molecular subtypes, suggesting different pathways of tumorigenesis and progression.^17^ Therefore, considering molecular markers that differentiate these pathways is crucial for a deeper understanding of how lifestyle factors, like physical activity, influence CRC risk. Previous research has identified variations in the relationship between smoking and CRC risk based on these molecular markers,^61^ however, evidence regarding physical activity remains limited. Our findings indicate that the inverse association of physical activity with CRC is not specific to any specific molecular phenotype of CRC. This aligns with a smaller pooled analysis from 3 CRC patient cohorts, namely the Assessment of Targeted Therapies Against Colorectal Cancer, the biomarker-based protocol (Integromics), and The Cancer Genome Atlas, which found no differentiation in the association of physical activity between CpG island methylator phenotype–low/negative and CpG island methylator phenotype–high tumors.^62^ Similarly, long-term vigorous physical activity was associated with a lower risk of CpG island methylator phenotype–low and CpG island methylator phenotype–high tumors, using data from a large population-based case-control study of incident colon cancer.^63^ These results suggest that the benefits of physical activity may apply broadly across various CRC molecular subtypes, reinforcing its importance in CRC prevention strategies.

This is the largest study investigating the association between physical activity and the risk of CRC by tumor molecular subtypes. A major strength of our study is the ability to pool individual-level data from 5 observational studies with available information on physical activity measurements and tumor-marker status, providing insights into how physical activity is associated with different pathways of colorectal tumorigenesis. Another key strength is that we triangulated the results, employing a Mendelian randomization analysis. Triangulation integrates evidence from different approaches, each with different and unrelated sources of bias, to potentially strengthen causal inference.^64^ Some limitations should be considered when interpreting the findings. Our analysis used well-known molecular subtypes (CpG island methylator phenotype, microsatellite instability, *KRAS*, *BRAF*). Nevertheless, comprehensive tumor characterization, including next-generation sequencing of somatic mutations to include genome-wide DNA methylation analysis, histone modification profiling, gene expression studies, immune profiling through protein-based assays, and spatial transcriptomics could allow a deeper classification of tumor subtypes. The included studies used different tools to assess physical activity, introducing heterogeneity, and some studies measured recreational and others undifferentiated physical activity. To deal with this heterogeneity, we used *z* scores of physical activity in the analysis. In the observational pooled analysis, physical activity data were obtained through self-reports, which can be subject to social desirability and approval biases,^65^ but the results were not different when we performed Mendelian randomization based on accelerometer-derived activity. Investigation by Jass subtypes resulted in small sample sizes for some types because of their rarity, which may have limited statistical power to detect modest associations. Despite the adjustment for several potential confounders, we cannot rule out the possibility of unmeasured and residual confounding, although further adjustments in sensitivity analyses did not alter the results. Finally, this study included predominantly White populations (United States, Europe, Canada, and Australia); therefore, the findings may not be generalizable to other racial and ethnic groups.

In conclusion, this study confirms the benefits of physical activity on CRC risk, especially in males. The evidence suggests that physical activity does not differentially influence the major molecular subtypes, defined by microsatellite instability, CpG island methylator phenotype, BRAF, and KRAS involved in colorectal carcinogenesis. Maintaining regular physical activity plays a vital role in preventing colorectal cancer.

## Supplementary Material

pkaf095_Supplementary_Data

## Data Availability

Tumor markers and epidemiologic data are available on request and permission. Please contact gecco@fredhutch.org to request the standardized proposal form. The principal investigators of each contributing study will evaluate and approve the proposal, and data access will be managed centrally.
