# Supplementary material for: Physical activity and molecular subtypes of colorectal cancer: a pooled observational analysis and Mendelian randomization study
Source: JNCI Cancer Spectr. 2025 Oct 1;9(6):pkaf095. doi: 10.1093/jncics/pkaf095 (PMC12596107; doi:10.1093/jncics/pkaf095)
Supplement: pkaf095_Supplementary_Data [file pkaf095_supplementary_data.docx]

**Physical activity and molecular subtypes of colorectal cancer: A pooled observational analysis and Mendelian randomisation study**

**Supplements**

[**Figure S1**. Distribution of MET-hr/wk by study before and after standardisation 11](#_Toc206406098)

[**Figure S2**. Diagnostic trace plots for the convergence of the Multiple Imputation by Chained Equations (MICE). The plots show the mean and standard deviation of the imputed values of each variable at each iteration of the imputation process. Each coloured line shows a different imputed dataset. 12](#_Toc206406099)

[**Figure S3**. Density plots of continuous variables comparing the observed (blue line) and imputed (red lines) values. 13](#_Toc206406100)

[**Figure S4**. Density plots of categorical variables comparing the observed (black bar) and imputed (blue bars) values 14](#_Toc206406101)

[**Figure S5**. Meta-analysis of the association between physical activity (per 1 standard deviation increase in MET-hr/week) and colorectal cancer risk across five studies. 16](#_Toc206406102)

[**Table S1**. Description of participating studies in observational or Mendelian randomisation analysis. 2](#_Toc206405979)

[**Table S2**. Summary of study-specific assessment of microsatellite instability (MSI) status. 7](#_Toc206405980)

[**Table S3**. Summary of study-specific CpG Island Methylation Phenotype (CIMP) status assessment. 9](#_Toc206405981)

[**Table S4**. Baseline characteristics overall and by individual study used in the Mendelian randomisation analysis. 14](#_Toc206405982)

[**Table S5**. Sample size by molecular subtype status and sex of the summary statistics used in Mendelian randomisation analysis. 15](#_Toc206405983)

[**Table S6**. Association between physical activity, colorectal cancer, and its molecular subtypes^a, b, c^ stratified by study design. 16](#_Toc206405984)

[**Table S7**. Association between physical activity, proximal colon cancer, and its molecular subtypes^a, b, c^. 18](#_Toc206405985)

[**Table S8**. Association between physical activity, proximal colon cancer, and its molecular subtypes stratified by study design ^a, b, c^. 19](#_Toc206405986)

[**Table S9**. Association between physical activity, distal colon cancer, and its molecular subtypes^a, b, c^. 21](#_Toc206405987)

[**Table S10**. Association between physical activity, distal colon cancer, and its molecular subtypes stratified by study design ^a, b, c^. 22](#_Toc206405988)

[**Table S11**. Association between physical activity, colon (proximal and distal) cancer, and its molecular subtypes^a, b, c^. 24](#_Toc206405989)

[**Table S12**. Association between physical activity, colon (proximal and distal) cancer, and its molecular subtypes stratified by study design^a, b, c^. 25](#_Toc206405990)

[**Table S13**. Association between physical activity, rectal cancer, and its molecular subtypes^a, b, c^. 27](#_Toc206405991)

[**Table S14**. Association between physical activity, rectal cancer, and its molecular subtypes stratified by study design ^a, b, c^. 28](#_Toc206405992)

[**Table S15**. Association between physical activity, colorectal cancer, and its molecular subtypes^a, b, c^ stratifying by early and later-onset. 30](#_Toc206405993)

[**Table S16**. Association between physical activity, colorectal cancer, and its molecular subtypes according to the observational analysis after conducting multiple imputation^a, b, c^. 31](#_Toc206405994)

[**Table S17**. Genetic instrument-physical activity and instrument-colorectal cancer molecular subtypes associations used for the Mendelian randomisation analyses. 32](#_Toc206405995)

[**Table S18**. Results from Mendelian randomisation analysis for physical activity and risk of colorectal cancer defined by molecular markers. 34](#_Toc206405996)

**Table S1**. Description of participating studies in observational or Mendelian randomisation analysis.

| **Study name** | **Abbreviation** | **Design** | **Country** | **Matching factors** | **Analysis inclusion** |
| --- | --- | --- | --- | --- | --- |
| Cancer Prevention Study II | CPSII | Cohort | United States | Age, sex, race, date of blood draw | Observational  Mendelian randomisation |
| Health Professionals Follow-up Study | HPFS | Cohort | United States | Age, month/year of blood sampling | Observational  Mendelian randomisation |
| Nurses’ Health Study | NHS | Cohort | United States | Age, month/year of blood sampling | Observational  Mendelian randomisation |
| Darmkrebs: Chancen der Verhütung durch Screening Study | DACHS | Case-control | Germany | Age, sex, county of residence | Observational  Mendelian randomisation |
| Diet, Activity and Lifestyle Study | DALS | Case-control | United States | Age, sex | Observational  Mendelian randomisation |
| Colon Cancer Family Registry | CCFR_Australia | Case-control | Australia | Age, sex | Mendelian randomisation |
| Colon Cancer Family Registry | CCFR_Ontario | Case-control | Canada | Age, sex | Mendelian randomisation |
| Colon Cancer Family Registry | CCFR_Seattle | Case-control | United States | Age, sex | Mendelian randomisation |
| Early Detection Research Network | EDRN | Case–control | United States | Age, sex | Mendelian randomisation |
| European Prospective Investigation into Cancer and Nutrition_Sweden | EPIC_Sweden | Cohort | Sweden | Age, sex, study center, follow-up time, time of day of blood collection, fasting status, menopausal status, phase of menstrual cycle at blood collection | Mendelian randomisation |
| Melbourne Collaborative Cohort Study | MCCS | Cohort | Australia | Sex, country of birth, year of baseline attendance | Mendelian randomisation |
| Newfoundland Familial Colorectal Cancer Registries | NFCCR | Case-control | Canada | Age, sex | Mendelian randomisation |
| Northern Sweden Health and Disease Study | NSHDS | Cohort | Sweden | Subcohort, age, sex, age and year of blood sampling, fasting status | Mendelian randomisation |

# Description of included studies

Cancer Prevention Study-II (CPS-II)

The CPS-II Nutrition cohort (established in 1992) is a prospective study of cancer incidence and mortality in the United States (1,2). All participants filled out a self-administered questionnaire with information on demographical, medical, dietary, and lifestyle factors. Biennial follow-up questionnaires have been sent out since 1997 to collect continuous information about current exposures and new cancer diagnoses. All reported cancers are verified through medical records, state cancer registry linkage, or death certificates. Controls were matched on race, gender, and age. The Emory University Institutional Review Board approves all aspects of the CPS-II Nutrition Cohort.

Health Professionals Follow-up Study (HPFS)

The HPFS was started in 1986 to evaluate underlying cardiovascular disease and cancer etiologies. It originally included 51,529 male health professionals in the United States who all completed a detailed questionnaire on health and diet. The all-male study was designed to complement the all-female Nurses’ Health Study, which examines similar hypotheses. Colorectal cancer and other outcomes were reported by participants or next-of-kin and were followed up through a review of the medical and pathology records by physicians. Overall, more than 97% of self-reported colorectal cancers were confirmed by medical record review. Information on histology and the primary anatomical location of the tumour was abstracted. Follow-up evaluation has been excellent, with 94% of the men responding to date. Patients with available tumour molecular characterisation were included in this study.

Nurses’ Health Study (NHS)

The NHS cohort, initiated in 1976, initially included information on health-related exposures from 121,700 married female registered nurses aged 30-55 (3). Since 1976, follow-up questionnaires have been mailed every two years. Colorectal cancer and other outcomes were reported by participants or next-of-kin and followed up through a review of the medical and pathology records by physicians. Overall, more than 97% of self-reported colorectal cancers were confirmed by medical-record review. Information was abstracted on histology and the primary anatomical location of the tumour. The rate of follow-up evaluation has been high: as a proportion of the total possible follow-up time, follow-up evaluation has been more than 92%. Colorectal cancer cases were ascertained through June 1, 2008.

Melbourne Collaborative Cohort Study (MCCS)

The MCCS is a prospective study, run between 1990 and 1994, that recruited 41,514 healthy adult participants aged between 27 and 76 years (99% aged 40-69) from the Melbourne metropolitan area (4). This study aimed to examine the role of lifestyle factors in the risk of cancer and heart disease. Incident cases of colorectal cancer were identified through linkage to population-based cancer registries in Australia. Cases included participants with a histopathological diagnosis of invasive colorectal adenocarcinoma diagnosed after baseline. Participants provided informed consent and sufficient FFPE material for somatic testing. Study protocols were approved by the Human Research Ethics Committee at the Cancer Council Victoria.

Darmkrebs: Chancen der Verhütung durch Screening Study (DACHS)

DACHS is a large German population-based case-control study started in 2003 in the Rhine-Neckar-Odenwald region (southwest region of Germany) (5,6). The purpose of DACHS was to assess the potential of endoscopic screening for the reduction of colorectal cancer risk and to investigate etiologic determinants of the disease, particularly lifestyle/environmental factors and genetic factors. Briefly, cases with a first diagnosis of invasive colorectal cancer (ICD-10 codes C18-C20) who were at least 30 years of age, German speaking, resident in the study region, and mentally and physically able to participate in a one-hour interview, were recruited by their treating physicians either in the hospital a few days after surgery, or by mail after hospital discharge. Cases were confirmed by histologic reports and hospital discharge letters following the diagnosis of colorectal cancer. All hospitals treating colorectal cancer patients in the study region participated. Community-based controls were randomly selected from population registries, employing age frequency matching (5-year groups), sex, and county of residence. Controls without a history of colorectal cancer were contacted by mail and follow-up calls. Data on demographics, medical history, family history of colorectal cancer, and various lifestyle factors were collected during an in-person interview. Participants also donated blood and mouthwash samples.

Diet, Activity and Lifestyle Study (DALS)

DALS, described in more detail elsewhere (7,8), was a population-based, case-control study of colon cancer. Participants were recruited between 1991 and 1994 from 3 locations: the Kaiser Permanente Medical Care Program of Northern California, an 8-county area in Utah, and Minnesota's metropolitan Twin Cities area. Eligibility criteria for cases included age at diagnosis between 30 and 79 years, diagnosis with first primary colon cancer (International Classification of Disease for Oncology, Second Edition, 18.0 and 18.2–18.9) between October 1, 1991, and September 30, 1994, English speaking, and competency to complete the interview. Individuals with cancer of the rectosigmoid junction or rectum were excluded, as were those with a pathology report noting familial adenomatous polyposis, Crohn’s disease, or ulcerative colitis. A rapid-reporting system was used to identify all incident cases of colon cancer, resulting in most cases being interviewed within 4 months of diagnosis. Controls from the Kaiser Permanente Medical Care Program were selected randomly from membership lists. In Utah, controls younger than 65 years of age were selected randomly through random-digit dialling and driver’s license lists. Controls 65 years of age and older were selected randomly from Health Care Financing Administration lists. In Minnesota, controls were identified from Minnesota driver’s license or state identification lists. Patients with available tumour molecular characterisation were included in this study.

Early Detection Research Network (EDRN)

The EDRN initiative aims to develop and sustain a biorepository that supports translational research (9). High-quality biospecimens from colorectal cancer patients ages 18 years or above were accrued and annotated with pertinent clinical, epidemiologic, molecular and genomic information. Information on molecular markers was abstracted from patient medical records, and colorectal cancer with available MSI, CIMP, KRAS mutation, or BRAF mutation characterisation was included in this study.

European Prospective Investigation into Cancer (EPIC) – Sweden

EPIC is an ongoing multicenter prospective cohort study designed to investigate the associations between diet, lifestyle, genetic and environmental factors and various types of cancer (10). Briefly, 521,448 participants (~70% women), mostly aged 35 years or above, were recruited between 1992 and 2000. Participants were recruited from 23 study centres in ten European countries. All study participants provided written informed consent, and ethical approval for the EPIC study was obtained from the review boards of IARC and local participating centres. The current study included participants from the northern Swedish EPIC-Umeå site, the Västerbotten Intervention Study (VIP). Colorectal cancer cases were identified by linkage with the Cancer Registry of Northern Sweden, which reports to the Swedish Cancer Registry, and were verified by a gastrointestinal pathologist. Controls were selected from the full cohort of individuals who were alive and free of cancer (except non-melanoma skin cancer) at the time of case diagnosis.

Northern Sweden Health and Disease Study (NSHDS)

The NSHDS is a population-based study that includes residents of Västerbotten County in Northern Sweden (11). It includes more than 110,000 participants, of which approximately one-third have repeated samples, from three population-based cohorts: the Västerbotten Intervention Project (VIP), the Northern Sweden WHO Monitoring of Trends and Cardiovascular Disease (MONICA) Study, and the local Mammography Screening Project (MSP). The VIP cohort, which makes up approximately 85% of the NSHDS, aims to invite all residents of Västerbotten County to a health examination upon turning 30 (some years), 40, 50 and 60 years of age. It was established in 1985 and continues to recruit participants. In both the VIP and MONICA cohorts, extensive measured and self-reported health and lifestyle data were collected, whereas data in the MSP are more limited. Blood samples for research purposes are collected in all three cohorts. The NSHDS is a part of EPIC, and the selection of colorectal cases and controls were as described for EPIC-Sweden.

Colon Cancer Family Registry (CCFR)

The CCFR (www.coloncfr.org) is a National Cancer Institute-supported consortium of six centres (12). The CCFR includes data from approximately 42,500 subjects (10,500 case probands and 26,900 unaffected and affected relatives, 4,280 unrelated population-based controls, and 920 spouse controls). The study recruited cases and unaffected controls (aged 20 to 74 years) beginning in 1998. All participants self-completed a standardised questionnaire that included questions about established and suspected risk factors for colorectal cancer, including questions on medical history and medication use, reproductive history (for female participants), family history, physical activity, demographics, alcohol and tobacco use, and dietary factors. Colorectal case and population-based control participants from three of the six participating centres (Seattle-SCCFR, Australia-ACCFR, Ontario-OFCCR) were included in this study.

# Harmonization of Colorectal Tumor Marker Data

Microsatellite Instability (MSI) Status

The included studies used polymerase chain reaction (PCR) to assess microsatellite status. The specific markers assessed using PCR-based methods are summarised in Table S2. Two categories were created for downstream analyses to harmonise markers across all studies: MSI-high and non-MSI-high. For studies that categorised MSI status as MSI-high (MSI-H), MSI-low (MSI-L), and MSS, we collapsed MSI-L and MSS into the non-MSI-high category. Tumour classification was based on >5 interpretable markers for CPS-II (unless all four markers were unstable, in which case the tumour was classified as MSI), and >7 interpretable markers for NHS and HPFS (13). For these studies, tumours were classified as MSI-high (MSI-H) if 30% or more of the markers showed instability and non-MSI-high if < 30% and > 0% showed instability or if no marker exhibited instability. DALS, which conducted MSI testing before developing the Bethesda Consensus Panel (14) , determined MSI based on the mononucleotides BAT26 and TGFBR2 (TGFβRII) and a panel of 10 tetranucleotide repeats (15–17). These have been shown to correlate highly with the Bethesda Panel (18). A tumour classification of unstable was given if the panel of 10 tetranucleotides, BAT26, or TGFBR2 were determined as unstable. Tumoral and normal DNA were PCR amplified with these 12 primer sets, and MSI was defined as > 1 new PCR product, either smaller or larger than those produced from normal DNA. Specifically, for BAT26, the PCR product from the tumour had to be >4 base pairs smaller than that from the germline. A tumour classification of MSI from the tetranucleotide repeat panel was based on > 30% markers showing instability and MSS if <30% of repeats were unstable, with > 6 interpretable markers of the 10 evaluated. DACHS (19) determined MSI status using a mononucleotide marker panel (20) that has high concordance with the National Cancer Institute Bethesda Consensus Panel (14).

**Table S2**. Summary of study-specific assessment of microsatellite instability (MSI) status.

| **Study** | **Markers*/Proteins** | **Threshold for Interpretability** | **Definitions** |
| --- | --- | --- | --- |
| **CPSII** | BAT25, BAT26, BAT40,  BAT34C4, ACTC, D10S197,  D17S250, D18S55, D5S346, MYCL | >5 interpretable markers  (Unless 4 markers were  unstable) | * MSI-H if >30% markers showed instability  * MSI-L if <30% and >0% showed instability  * MSS if no marker exhibited instability |
| **HPFS** | BAT25, BAT26, BAT40, D18S55, D18S56, D18S67, D18S487, D2S123, D5S346, D17S250 | >7 interpretable markers | * MSI-H if >30% markers showed instability  * MSI-L if <30% and >0% showed instability  * MSS if no marker exhibited instability |
| **NHS** | BAT25, BAT26, BAT40, D18S55, D18S56, D18S67, D18S487, D2S123, D5S346, D17S250 | >7 interpretable markers | * MSI-H if >30% markers showed instability  * MSI-L if <30% and >0% showed instability  * MSS if no marker exhibited instability |
| **DACHS** | BAT25, BAT26, CAT25 | All 3 markers interpretable | * MSI- high if >1 marker showed instability |
| **DALS** | BAT26, TGFBRII | >6 of 10 markers be interpretable from tetranucleotide repeat panel | * MSI: Instability in BAT26, TGFBRII, or 10 tetranucleotide marker panel. - 10 marker panel: >30% unstable repeats. |
| **CCFR** | BAT25, BAT26, BAT40, BAT34C4, D5S346, D17S250, ACTC, D18S55, D10S197, MYCL | >4 interpretable markers | * MSI-high if >30% markers showed instability |
| **EDRN** | BAT-25, BAT-26, CAT25 | - | - |
| **EPIC_Sweden** | MLH1, MSH2, MSH6, and PMS2 | Immunohistochemistry | Immunohistochemical detection of deficiency for selected mismatch repair proteins was used to determine MSI status. |
| **MCCS** | BAT25, BAT26, BAT40, BAT34C4, D5S346, D17S250, ACTC, D18S55, D10S197, MYCL | >4 interpretable markers | * MSI-high if >30% markers showed instability |
| **NSHDS** | MLH1, MSH2, MSH6, and PMS2 | Immunohistochemistry | Immunohistochemical detection of deficiency for selected mismatch repair proteins was used to determine MSI status. |
| **NFCCR** | BAT-25, BAT-26, BAT-40, BAT-34C4, D5S346, D17S250, ACTC, D18S55, D10S197, MYCL | >4 interpretable markers | * MSI-high if >30% markers showed instability |

*BRAF* and *KRAS* Mutation Status

Studies used PCR, sequencing, and IHC techniques to assess *BRAF* and *KRAS* mutations. Most studies evaluated the c.1799T>A (p.V600E) mutation in *BRAF* exon 15 and *KRAS* mutations in codons 12 and 13, though a few evaluated additional loci. In analyses, we included any mutation identified by at least one study. CPS-II used PCR to assess *BRAF* c.1799T>A (p.V600E) mutations and *KRAS* codon 12, 13, and 14 mutations. HPFS and NHS performed PCR and pyrosequencing to identify *BRAF* codon 600 mutations (21–23). HPFS and NHS used PCR and pyrosequencing to identify *KRAS* mutations in codons 12, 13, 61, and 146 (21,24). DACHS (6) used Sanger sequencing and IHC analysis of V600E expression to determine BRAF mutation status. For sequencing, they amplified exon 15 of BRAF using FideliTaq polymerase and sequenced using the BigDye Terminator v1.1 Cycle Sequencing Kit on an ABI 3500 Genetic Analyzer. DACHS determined *KRAS* mutation status by a single-stranded conformational polymorphism technique (SSCP) or Sanger sequencing, as reported previously (6).

DALS amplified exon 15 using Applied Biosystems AmpliTaq Gold and sequencing (25).

CpG Island Methylator Phenotype Status

Studies used gene promoter methylation analysis to determine CIMP status. The specific genes assessed in each study are shown in Table S3. Similar to the harmonisation of MSI status, we created two CIMP categories for downstream analyses, CIMP-high and CIMP-low/negative. In instances where studies categorised CIMP-high, CIMP-low, and CIMP-negative, we collapsed CIMP-low and CIMP-negative into the CIMP-low/negative category. HPFS, NHS (22,26), and CPS-II (27) used the MethyLight (28) method to determine CIMP status. HPFS, NHS, and CPS-II used a panel of eight genes. The percent of methylated reference (PMR) value was calculated, and for CPS-II, a gene was considered positive for methylation when the PMR>10. HPFS and NHS used a PMR cutoff value of >4 for CDKN2A, MLH1, CACNA1G, NEUROG1, RUNX3, SOCS1, and a PMR of >6 for CRABP1 and IGF2. HPFS, NHS, and CPS-II classified tumours with ≥6 methylated markers as CIMP-high, 1-4 markers as CIMP-low, and no markers as CIMP-negative. MCCS classified tumours with >3 methylated markers as CIMP-high and, otherwise, as CIMP-low/negative. DACHS determined CIMP status using a panel of five genes and methods described previously (29) . They determined methylation status from the methylation-specific PCR based on the presence or absence of amplified product and classified tumours with ≥3/5 methylated markers as CIMP-high. DALS determined CIMP status using a classic panel of CpG islands (30,31). Tumours with ≥3/5 methylated markers were classified as CIMP-high, and no methylated markers were classified as CIMP-low/negative, with three or more loci successfully evaluated.

**Table S3**. Summary of study-specific CpG Island Methylation Phenotype (CIMP) status assessment.

| **Study** | **Markers*/Proteins** | **Threshold for Interpretability** | **Definitions** |
| --- | --- | --- | --- |
| **CPSII** | CDKN2A, MLH1, CACNA1G, NEUROG1, RUNX3, SOCS1, IGF2, CRABP1 | PMR >4 (>6 for CRABP1, IGF2) | ≥5/8 methylated markers |
| **HPFS** | CDKN2A, MLH1, CACNA1G, NEUROG1, RUNX3, SOCS1, IGF2, CRABP1 | PMR > 4 for CDKN2A, MLH1, CACNA1G, NEUROG1, RUNX3, SOCS1.  PMR > 6 for CRABP1, IGF2 | ≥5/8 methylated markers |
| **NHS** | CDKN2A, MLH1, CACNA1G, NEUROG1, RUNX3, SOCS1, IGF2, CRABP1 | PMR > 4 for CDKN2A, MLH1, CACNA1G, NEUROG1, RUNX3, SOCS1.  PMR > 6 for CRABP1, IGF2 | ≥5/8 methylated markers |
| **DACHS** | MGMT, MLH1, MINT1, MINT2, MINT31 | N/A | ≥3/5 methylated markers |
| **DALS** | MINT1, MINT2, MINT31, CDKN2A9, and hMLH1 | N/A | ≥3/5 methylated markers |
| **CCFR** | CACNA1G, IGF2, NEUROG1, RUNX3, and SOCS1 | PMR > 10 | >3 methylated markers |
| **EDRN*** | N/A | N/A | N/A |
| **EPIC_Sweden** | CDKN2A, MLH1, CACNA1G, NEUROG1, RUNX3, SOCS1, IGF2, CRABP1 | PMR > 10 | ≥5/8 methylated markers |
| **MCCS** | CACNA1G, IGF2, NEUROG1, RUNX3, and SOCS1 | PMR > 10 | ≥3/5 methylated markers |
| **NFCCR*** | N/A | N/A | N/A |
| **NSHDS** | CDKN2A, MLH1, CACNA1G, NEUROG1, RUNX3, SOCS1, IGF2, CRABP1 | PMR > 10 | ≥5/8 methylated markers |


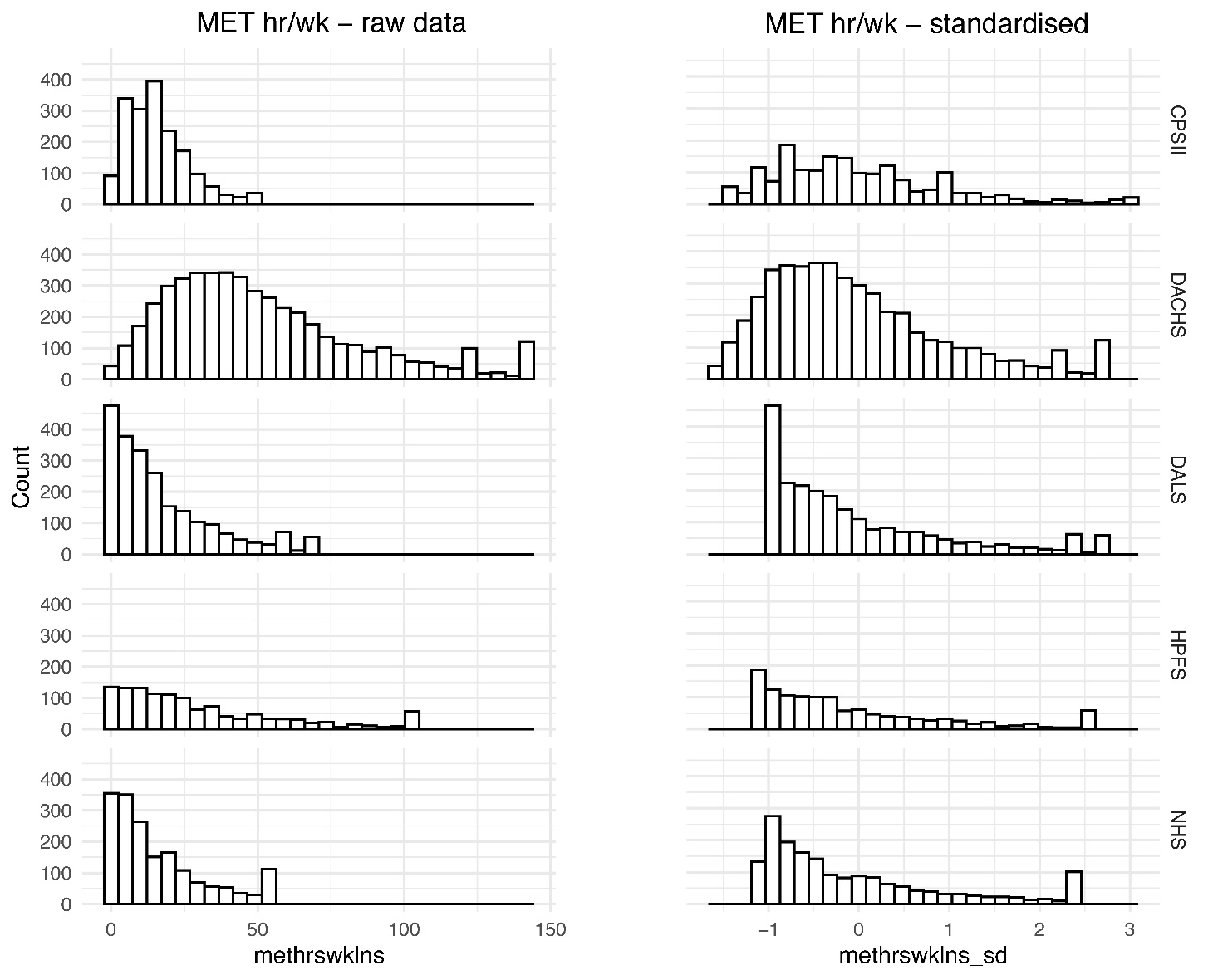


**Figure S1**. Distribution of MET-hr/wk by study before and after standardisation


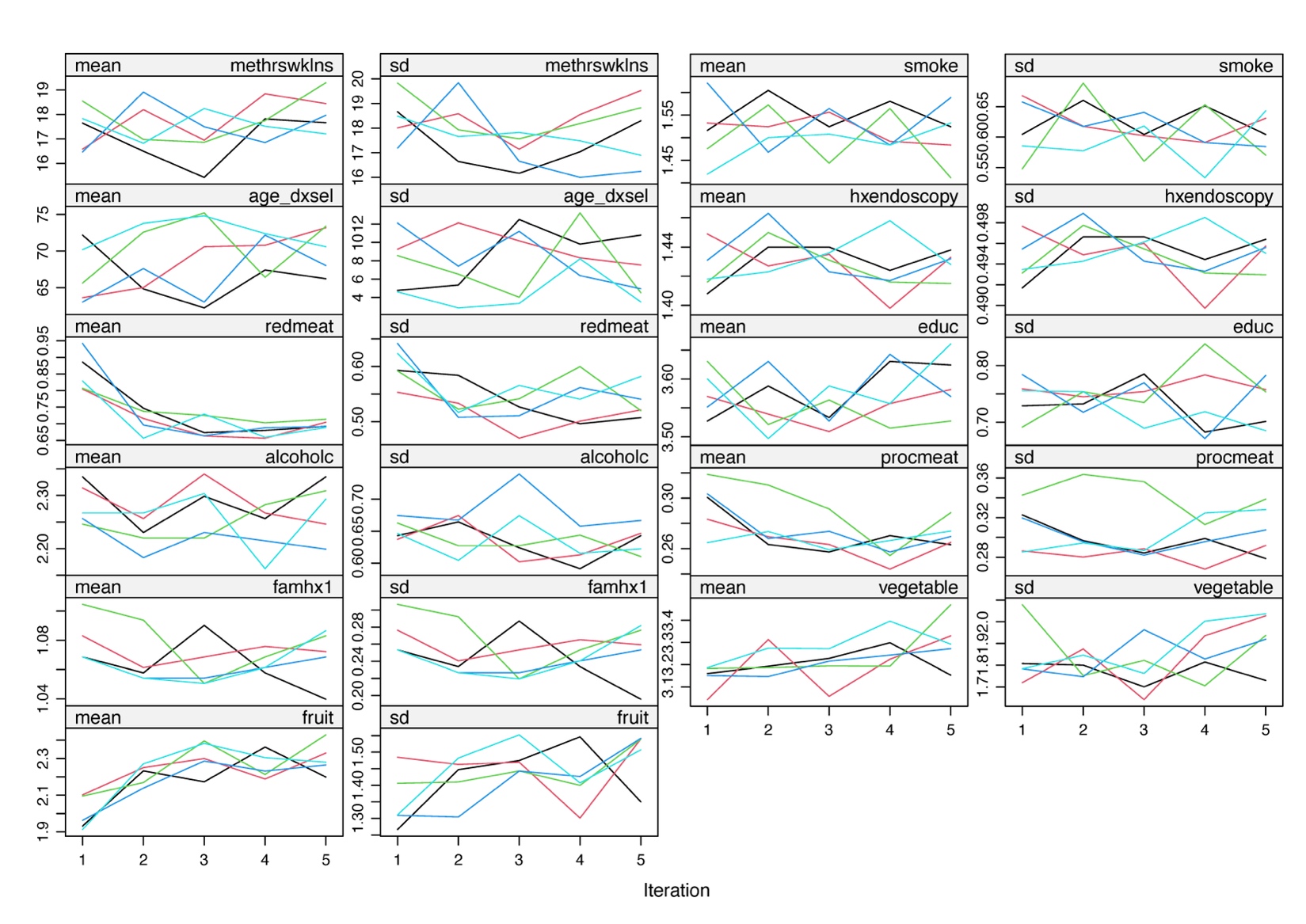


**Figure S2**. Diagnostic trace plots for the convergence of the Multiple Imputation by Chained Equations (MICE). The plots show the mean and standard deviation of the imputed values of each variable at each iteration of the imputation process. Each coloured line shows a different imputed dataset.


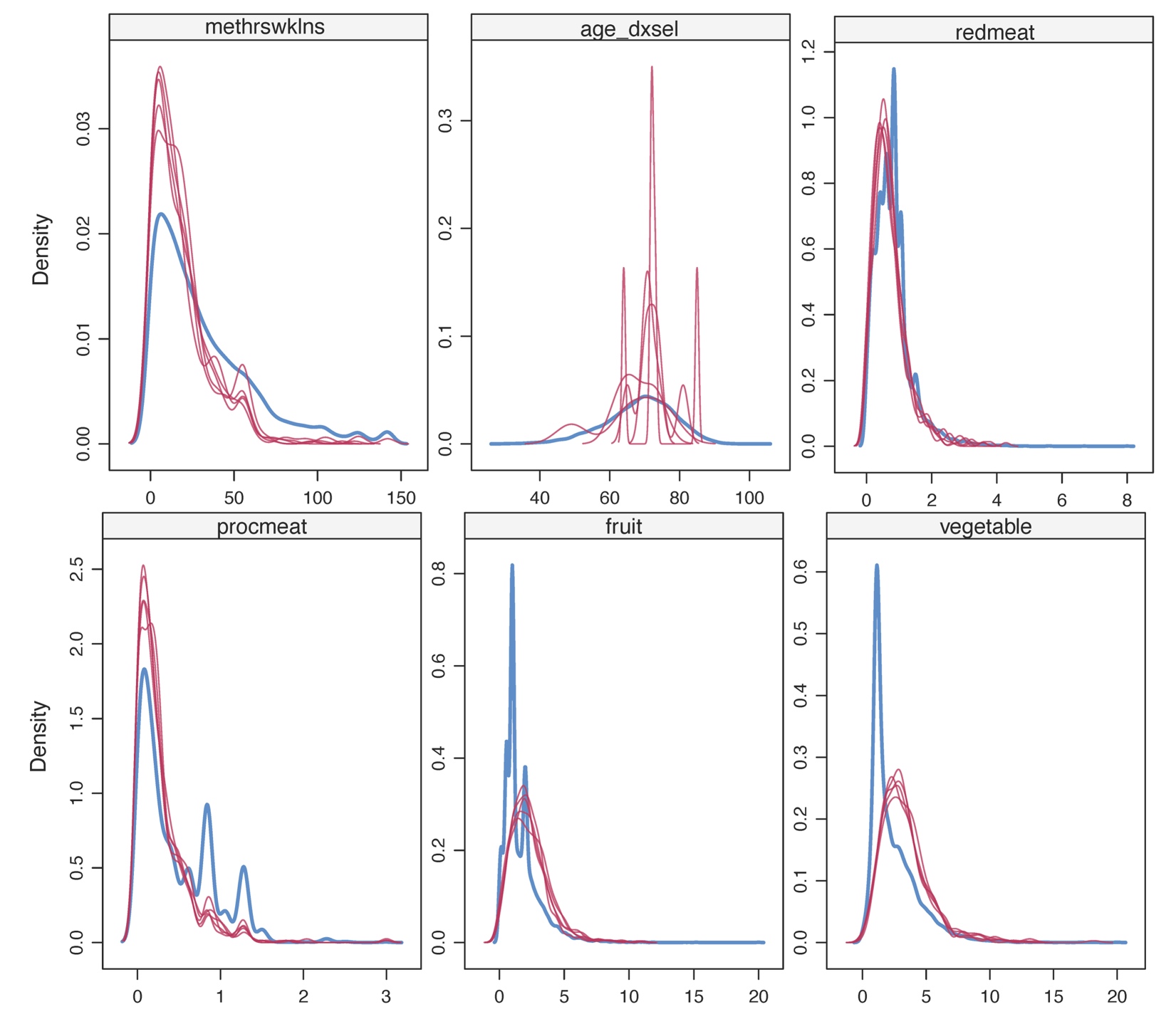


**Figure S3**. Density plots of continuous variables comparing the observed (blue line) and imputed (red lines) values.


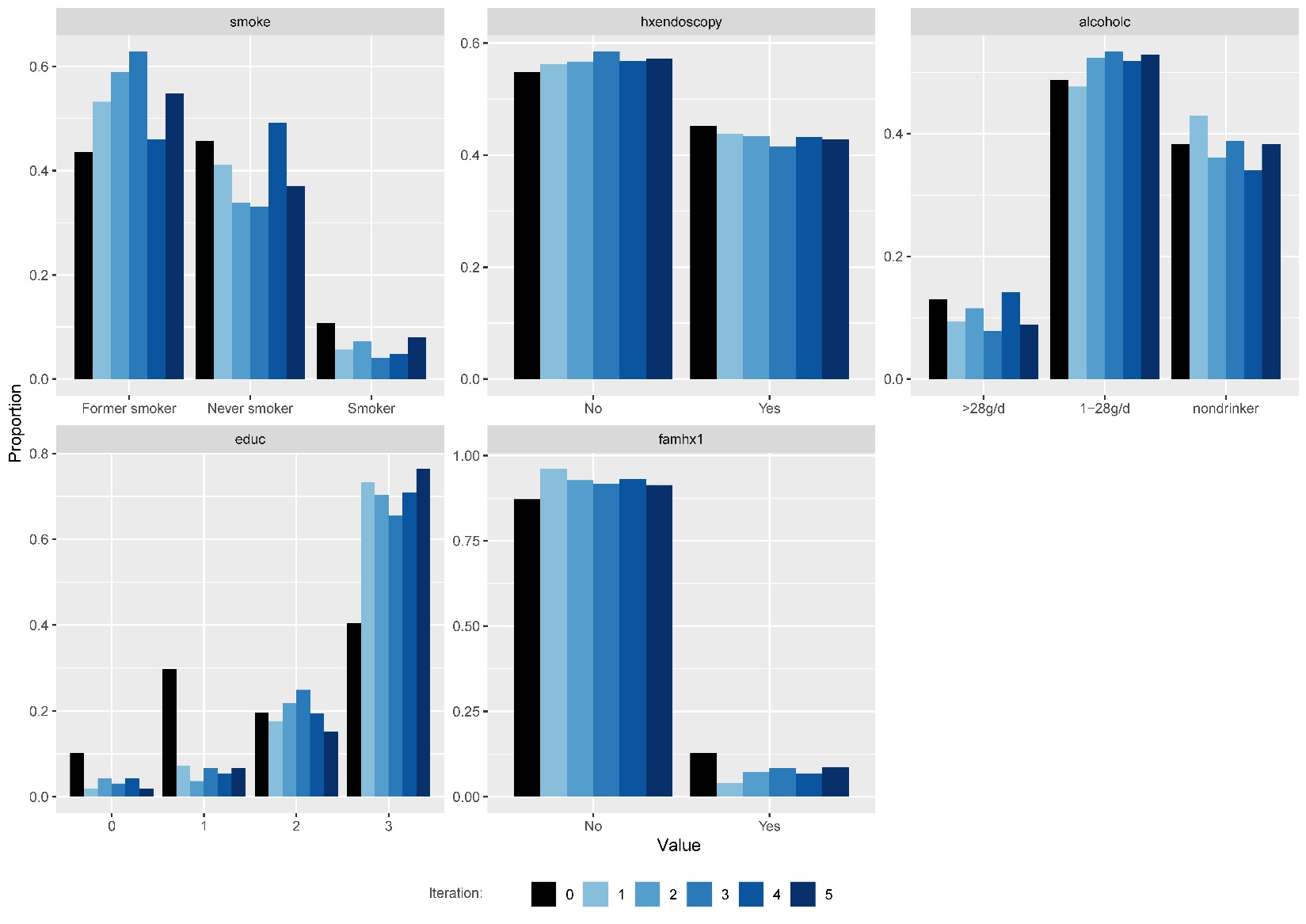


**Figure S4**. Density plots of categorical variables comparing the observed (black bar) and imputed (blue bars) values

**Table S4**. Baseline characteristics overall and by individual study used in the Mendelian randomisation analysis.

|  | **Overall** | **CCFR** | **CPSII** | **DACHS** | **DALS** | **EDRN** | **EPIC Sweden** | **HPFS** | **MCCS** | **NHS** | **NSHDS** |
| --- | --- | --- | --- | --- | --- | --- | --- | --- | --- | --- | --- |
| **Cases** |  |  |  |  |  |  |  |  |  |  |  |
| **N** | 8,178 | 3,421 | 330 | 1,813 | 841 | 198 | 144 | 330 | 477 | 306 | 318 |
| **Age of Diagnosis** | 62.4 (20-94) | 54.3 (20-83) | 76.1 (57-90) | 69.0 (33-94) | 65.5 (31-79) | 62.3 (26-87) | 64.0 (42-75) | 70.3 (45-91) | 67.8 (43-84) | 68.0 (37-86) | 64.2 (37-82) |
| **Sex** |  |  |  |  |  |  |  |  |  |  |  |
| Female | 4,299 (46.9) | 1,678 (49%) | 175 (53) | 763 (42.1) | 387 (46) | 76 (38.4) | 63 (43.8) | 0 (0%) | 223 (46.8) | 306 (100) | 203 (63.8) |
| Male | 4,873 (53.1) | 1,743 (51) | 155 (47) | 1,050 (57.9) | 454 (54) | 122 (61.6) | 81 (56.2) | 330 (100) | 254 (53.2) | 0 (0%) | 115 (36.2) |
| **Controls** |  |  |  |  |  |  |  |  |  |  |  |
| **N** | 10,472 | 2,629 | 883 | 2,168 | 1,162 | 312 | 381 | 595 | 673 | 1255 | 414 |
| **Age at Selection** | 65.2 (20-99) | 55.6 (20, 88) | 76.5 (57, 90) | 69 (34, 99) | 65.3 (30, 79) | 59.5 (20, 85) | 68.6 (45, 82) | 69.9 (44, 93) | 69.8 (43, 87) | 66.9 (37, 91) | 64.2 (35, 82) |
| **Sex** |  |  |  |  |  |  |  |  |  |  |  |
| Female | 5,292 (50.5) | 1,301 (49.5) | 441 (49.9) | 849 (39.2) | 525 (45.2) | 160 (51.3) | 175 (45.9) | 0 (0) | 325 (48.3) | 1,255 (100) | 261 (63) |
| Male | 5,180 (49.5) | 1,328 (50.5) | 442 (50.1) | 1,319 (60.8) | 637 (54.8) | 152 (48.7) | 206 (54.1) | 595 (100) | 348 (51.7) | 0 (0) | 153 (37) |
| Abbreviations: Colon Cancer Family Registry (CCFR), Cancer Prevention Study-II (CPS-II), Darmkrebs: Chancen der Verhütung durch Screening (DACHS), Diet, Activity, and Lifestyle Study (DALS), Early Detection Research Network (EDRN), European Prospective Investigation into Cancer (EPIC), Health Professionals Follow-up Study (HPFS), Melbourne Collaborative Cohort Study (MCCS), Nurses’ Health Study (NHS), Northern Sweden Health and Disease Study (NSHDS) | | | | | | | | | | | |

**Table S5**. Sample size by molecular subtype status and sex of the summary statistics used in Mendelian randomisation analysis.

| **Molecular subtype** | **Total** | **Males** | **Females** |
| --- | --- | --- | --- |
| **MSI** |  |  |  |
| MSI-high | 1,165 | 505 | 660 |
| MSS/MSI-L | 6,505 | 3,520 | 2,985 |
| **CIMP** |  |  |  |
| CIMP high | 1,000 | 383 | 617 |
| CIMP low/negative | 5,262 | 2,951 | 2,311 |
| ***BRAF*** |  |  |  |
| Mutated | 872 | 317 | 555 |
| Wild-type | 6,426 | 3,526 | 2,900 |
| ***KRAS*** |  |  |  |
| Mutated | 2,165 | 1,147 | 1,018 |
| Wild-type | 4,474 | 2,367 | 2,107 |


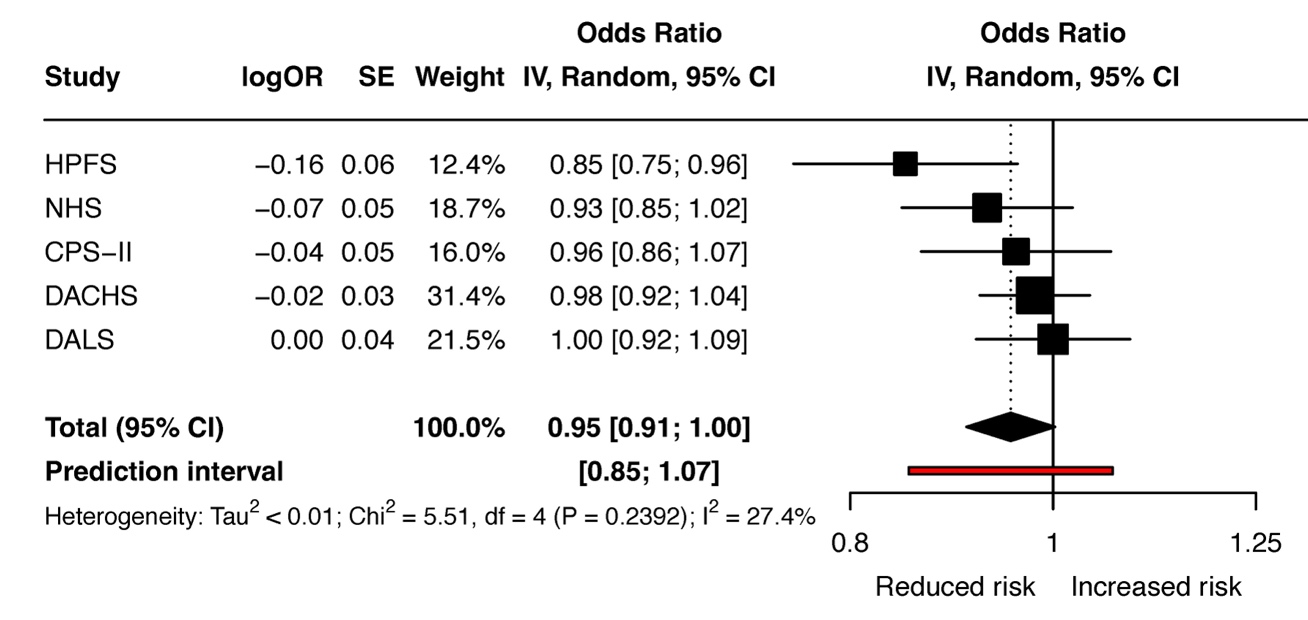


**Figure S5**. Meta-analysis of the association between physical activity (per 1 standard deviation increase in MET-hr/week) and colorectal cancer risk across five studies.

**Table S6**. Association between physical activity, colorectal cancer, and its molecular subtypes^a, b, c^ stratified by study design.

|  |  | **Microsatellite instability** | | **CpG island methylator phenotype** | | ***BRAF*** | | ***KRAS*** | |
| --- | --- | --- | --- | --- | --- | --- | --- | --- | --- |
| **MET hr/wk.** | **Overall CRC**  **OR (95%CI)** | **MSS/MSI-L**  **RRR (95%CI)** | **MSI-H**  **RRR (95%CI)** | **CIMP-low/negative**  **RRR (95%CI)** | **CIMP-high**  **RRR (95%CI)** | ***BRAF*-wild type**  **RRR (95%CI)** | ***BRAF*-mutated**  **RRR (95%CI)** | ***KRAS*-wild type**  **RRR (95%CI)** | ***KRAS*-mutated**  **RRR (95%CI)** |
| **Cohort studies** | | | | | | | | | |
| **Sex-combined** |  |  |  |  |  |  |  |  |  |
| No cases | 1,942 | 1,420 | 297 | 1,372 | 413 | 1,486 | 267 | 1,024 | 614 |
| -1.45 to -0.692 | Reference | Reference | Reference | Reference | Reference | Reference | Reference | Reference | Reference |
| -0.691 to 0.212 | 0.90 (0.78-1.05) | 0.86 (0.73-1.02) | 0.95 (0.71-1.327) | 0.86 (0.73-1.01) | 1.03 (0.80-1.33) | 0.90 (0.77-1.06) | 0.88 (0.65-1.18) | 0.90 (0.75-1.08) | 0.83 (0.67-1.03) |
| 0.211 to 3.025 | 0.79 (0.67-0.92) | 0.79 (0.68-0.94) | 0.68 (0.49-0.94) | 0.80 (0.68-0.96) | 0.76 (0.57-1.01) | 0.81 (0.68-0.96) | 0.64 (0.45-0.89) | 0.80 (0.66-0.97) | 0.77 (0.61-0.97) |
| P_trend_ | 0.0029 | 0.009 | 0.0215 | 0.0144 | 0.0569 | 0.0142 | 0.0085 | 0.0245 | 0.0279 |
| Per 1-SD | 0.89 (0.84-0.95) | 0.90 (0.84-0.96) | 0.86 (0.75-0.98) | 0.90 (0.84-0.96) | 0.89 (0.80-1.00) | 0.89 (0.83-0.96) | 0.87 (0.76-1.00) | 0.90 (0.83-0.97) | 0.89 (0.81-0.98) |
| P-value | 0.0005 | 0.0025 | 0.0238 | 0.03 | 0.0474 | 0.0014 | 0.0556 | 0.0093 | 0.0180 |
| P_difference_ | NA | 0.36 | | 0.67 | | 0.55 | | 0.90 | |
| **Males** |  |  |  |  |  |  |  |  |  |
| No cases | 972 | 747 | 110 | 714 | 153 | 796 | 84 | 498 | 312 |
| -1.45 to -0.692 | Reference | Reference | Reference | Reference | Reference | Reference | Reference | Reference | Reference |
| -0.691 to 0.212 | 0.77 (0.62-0.97) | 0.73 (0.57-0.93) | 0.98 (0.61-1.57) | 0.72 (0.56-0.91) | 1.00 (0.66-1.53) | 0.74 (0.59-0.94) | 0.89 (0.51-1.56) | 0.76 (0.58-0.99) | 0.73 (0.53-1.00) |
| 0.211 to 3.025 | 0.62 (0.49-0.78) | 0.64 (0.50-0.82) | 0.56 (0.32-0.96) | 0.62 (0.42-0.80) | 0.65 (0.41-1.05) | 0.60 (0.47-0.77) | 0.72 (0.40-1.29) | 0.59 (0.45-0.79) | 0.70 (0.50-0.98) |
| P_trend_ | 0.0001 | 0.0005 | 0.0353 | 0.0002 | 0.0689 | 0.0001 | 0.2601 | 0.0004 | 0.0388 |
| Per 1-SD | 0.83 (0.75-0.91) | 0.83 (0.75-0.92) | 0.81 (0.65-1.01) | 0.83 (0.75-0.92) | 0.81 (0.67-0.98) | 0.80 (0.73-0.89) | 0.94 (0.75-1.18) | 0.80 (0.71-0.90) | 0.87 (0.76-1.00) |
| P-value | 0.0001 | 0.0004 | 0.0601 | 0.0003 | 0.0280 | <0.0001 | 0.6162 | 0.0002 | 0.0445 |
| P_difference_ | NA | 0.70 | | 0.74 | | 0.20 | | 0.29 | |
| **Females** |  |  |  |  |  |  |  |  |  |
| No cases | 970 | 673 | 187 | 658 | 260 | 690 | 183 | 526 | 302 |
| -1.45 to -0.692 | Reference | Reference | Reference | Reference | Reference | Reference | Reference | Reference | Reference |
| -0.691 to 0.212 | 1.01 (0.83-1.24) | 0.99 (0.79-1.25) | 0.95 (0.66-1.36) | 1.00 (0.79-1.26) | 1.06 (0.77-1.47) | 1.07 (0.85-1.35) | 0.90 (0.63-1.29) | 1.04 (0.81-1.34) | 0.92 (0.68-1.25) |
| 0.211 to 3.025 | 0.96 (0.78-1.20) | 0.96 (0.76-1.23) | 0.79 (0.53-1.19) | 1.02 (0.80-1.30) | 0.85 (0.59-1.20) | 1.07 (0.84-1.36) | 0.62 (0.41-0.94) | 1.04 (0.80-1.35) | 0.82 (0.591.13) |
| P_trend_ | 0.6291 | 0.7687 | 0.2648 | 0.8821 | 0.3749 | 0.5951 | 0.0274 | 0.7818 | 0.2294 |
| Per 1-SD | 0.95 (0.87-1.04) | 0.96 (0.87-1.06) | 0.90 (0.76-1.06) | 0.96 (0.87-1.06) | 0.95 (0.85-1.09) | 0.98 (0.89-1.09) | 0.85 (0.71-1.01) | 1.00 (0.90-1.11) | 0.89 (0.72-1.02) |
| P-value | 0.3033 | 0.3793 | 0.2088 | 0.4558 | 0.4491 | 0.7517 | 0.0626 | 0.9767 | 0.1065 |
| P_difference_ | NA | 0.47 | | 0.84 | | 0.10 | | 0.17 | |
| **Case-controls studies** | | | | | | | | | |
| **Sex-combined** |  |  |  |  |  |  |  |  |  |
| No cases | 3,016 | 2,495 | 354 | 2,348 | 436 | 2,461 | 226 | 1,906 | 887 |
| -1.58 to -0.569 | Reference | Reference | Reference | Reference | Reference | Reference | Reference | Reference | Reference |
| -0.568 to 0.182 | 0.82 (0.73-0.93) | 0.81 (0.71-0.92) | 0.71 (0.54-0.93) | 0.83 (0.74-0.93) | 0.73 (0.57-0.93) | 0.82 (0.72-0.93) | 0.76 (0.54-1.06) | 0.76 (0.67-0.87) | 0.88 (0.74-1.05) |
| 0.181 to 2.73 | 0.92 (0.81-1.03) | 0.91 (0.80-1.03) | 0.91 (0.70-1.19) | 0.94 (0.83-1.06) | 0.81 (0.64-1.04) | 0.91 (0.80-1.03) | 0.94 (0.67-1.31) | 0.84 (0.74-0.97) | 0.91 (0.76-1.09) |
| P_trend_ | 0.1176 | 0.1185 | 0.4267 | 0.2947 | 0.0766 | 0.1135 | 0.6667 | 0.0093 | 0.3070 |
| Per 1-SD | 0.97 (0.92-1.02) | 0.97 (0.92-1.02) | 0.95 (0.84-1.07) | 0.98 (0.93-1.03) | 0.91 (0.81-1.01) | 0.97 (0.92-1.02) | 0.93 (0.80-1.08) | 0.94 (0.89-0.99) | 0.95 (0.88-1.03) |
| P-value | 0.1863 | 0.2517 | 0.3710 | 0.4041 | 0.0761 | 0.2454 | 0.3449 | 0.0332 | 0.2285 |
| P_difference_ | NA | 0.34 | | 0.14 | | 0.51 | | 0.65 | |
| **Males** |  |  |  |  |  |  |  |  |  |
| No cases | 1,746 | 1,459 | 158 | 1,409 | 207 | 1,446 | 96 | 1,085 | 524 |
| -1.58 to -0.569 | Reference | Reference | Reference | Reference | Reference | Reference | Reference | Reference | Reference |
| -0.568 to 0.182 | 0.84 (0.71-0.98) | 0.81 (0.68-0.95) | 0.76 (0.51-1.13) | 0.83 (0.71-0.99) | 0.71 (0.50-1.00) | 0.80 (0.68-0.94) | 0.91 (0.56-1.47) | 0.79 (0.66-0.95) | 0.86 (0.68-1.09) |
| 0.181 to 2.73 | 0.85 (0.73-0.99) | 0.86 (0.74-1.02) | 0.73 (0.49-1.09) | 0.90 (0.77-1.06) | 0.62 (0.44-0.88) | 0.88 (0.75-1.03) | 0.60 (0.35-1.01) | 0.79 (0.66-0.94) | 0.89 (0.71-1.12) |
| P_trend_ | 0.0402 | 0.0765 | 0.1218 | 0.2246 | 0.0069 | 0.1051 | 0.0652 | 0.0081 | 0.3153 |
| Per 1-SD | 0.95 (0.89-1.01) | 0.96 (0.90-1.03) | 0.88 (0.74-1.04) | 0.98 (0.92-1.05) | 0.81 (0.70-0.95) | 0.97 (0.91-1.03) | 0.78 (0.62-0.99) | 0.93 (0.86-1.00) | 0.95 (0.86-1.04) |
| P-value | 0.1166 | 0.2745 | 0.1317 | 0.5231 | 0.0088 | 0.3373 | 0.0411 | 0.0468 | 0.2897 |
| P_difference_ | NA | 0.28 | | **0.009** | | 0.05 | | 0.69 | |
| **Females** |  |  |  |  |  |  |  |  |  |
| No cases | 1,301 | 1,036 | 196 | 939 | 229 | 1,015 | 130 | 821 | 363 |
| -1.58 to -0.569 | Reference | Reference | Reference | Reference | Reference | Reference | Reference | Reference | Reference |
| -0.568 to 0.182 | 0.80 (0.67-0.95) | 0.83 (0.68-0.99) | 0.67 (0.46-0.98) | 0.83 (0.68-1.01) | 0.76 (0.54-1.07) | 0.85 (0.70-1.03) | 0.63 (0.40-1.01) | 0.75 (0.61-0.92) | 0.90 (0.69-1.19) |
| 0.181 to 2.73 | 1.03 (0.86-1.24) | 0.99 (0.82-1.21) | 1.13 (0.79-1.61) | 1.00 (0.82-1.22) | 1.07 (0.76-1.50) | 0.97 (0.79-1.18) | 1.27 (0.83-1.94) | 0.95 (0.77-1.17) | 0.95 (0.71-1.27) |
| P_trend_ | 0.6367 | 0.8305 | 0.6428 | 0.9064 | 0.8018 | 0.6603 | 0.3301 | 0.4914 | 0.7030 |
| Per 1-SD | 1.00 (0.92-1.08) | 0.98 (0.90-1.07) | 1.03 (0.87-1.21) | 0.98 (0.89-1.07) | 1.02 (0.87-1.19) | 0.97 (0.89-1.06) | 1.06 (0.87-1.30) | 0.96 (0.87-1.06) | 0.97 (0.85-1.10) |
| P-value | 0.8985 | 0.7176 | 0.7557 | 0.6155 | 0.7950 | 0.5495 | 0.5549 | 0.4328 | 0.5898 |
| P_difference_ | NA | 0.57 | | 0.49 | | 0.30 | | 0.91 | |

^a^ Controls are used as the reference for all odds ratios. CI, confidence interval; CIMP, CpG island methylator phenotype; CRC, colorectal cancer; MSI, microsatellite instability; MSS, microsatellite stable; OR, odds ratio.

^b^ Odds ratios are adjusted for study, age, sex (when not stratified), smoking status, education, red meat intake, and alcohol consumption.

^c^ Case-only analyses used to calculate *P*_difference_.

**Table S7**. Association between physical activity, proximal colon cancer, and its molecular subtypes^a, b, c^.

|  |  | **Microsatellite instability** | | **CpG island methylator phenotype** | | ***BRAF*** | | ***KRAS*** | |
| --- | --- | --- | --- | --- | --- | --- | --- | --- | --- |
| **MET hr/wk.** | **Proximal colon cancer**  **OR (95%CI)** | **MSS/MSI-L**  **RRR (95%CI)** | **MSI-H**  **RRR (95%CI)** | **CIMP-low/negative**  **RRR (95%CI)** | **CIMP-high**  **RRR (95%CI)** | ***BRAF*-wild type**  **RRR (95%CI)** | ***BRAF*-mutated**  **RRR (95%CI)** | ***KRAS*-wild type**  **RRR (95%CI)** | ***KRAS*-mutated**  **RRR (95%CI)** |
| **Sex-combined** | |  |  |  |  |  |  |  |  |
| No cases | 2,213 | 1,455 | 560 | 1,352 | 657 | 1,522 | 412 | 1,244 | 707 |
| -1.58 to -0.591 | Reference | Reference | Reference | Reference | Reference | Reference | Reference | Reference | Reference |
| -0.591 to 0.2 | 0.92 (0.82-1.04) | 0.97 (0.84-1.11) | 0.82 (0.66-1.01) | 0.96 (0.83-1.11) | 0.91 (0.75-1.11) | 0.94 (0.982-1.08) | 0.89 (0.70-1.13) | 0.92 (0.80-1.07) | 0.85 (0.70-1.02) |
| 0.2 to 3.03 | 0.95 (0.84-1.07) | 0.99 (0.86-1.14) | 0.81 (0.65-1.00) | 1.04 (0.90-1.21) | 0.84 (0.69-1.03) | 0.99 (0.86-1.14) | 0.85 (0.66-1.09) | 0.95 (0.82-1.11) | 0.87 (0.72-1.05) |
| P_trend_ | 0.4005 | 0.8644 | 0.0450 | 0.5839 | 0.0961 | 0.8383 | 0.1935 | 0.5094 | 0.1351 |
| Per 1-SD | 0.96 (0.92-1.02) | 0.98 (0.922-1.04) | 0.91 (0.83-1.00) | 1.00 (0.94-1.06) | 0.92 (0.84-1.00) | 0.98 (0.92-1.04) | 0.92 (0.82-1.03) | 0.98 (0.91-1.04) | 0.92 (0.84-1.00) |
| P-value | 0.1695 | 0.4863 | 0.0546 | 0.8978 | 0.0518 | 0.5147 | 0.1322 | 0.4569 | 0.0401 |
| P_difference_ | NA | 0.11 | | 0.07 | | 0.24 | | 0.35 | |
| **Males** |  |  |  |  |  |  |  |  |  |
| No cases | 1,083 | 770 | 212 | 725 | 255 | 810 | 137 | 564 | 396 |
| -1.58 to -0.591 | Reference | Reference | Reference | Reference | Reference | Reference | Reference | Reference | Reference |
| -0.591 to 0.2 | 0.83 (0.70-0.99) | 0.85 (0.70-1.03) | 0.81 (0.58-1.13) | 0.85 (0.70-1.04) | 0.79 (0.58-1.08) | 0.81 (0.67-0.98) | 0.94 (0.63-1.42) | 0.85 (0.68-1.05) | 0.75 (0.58-0.97) |
| 0.2 to 3.03 | 0.79 (0.67-0.94) | 0.85 (0.70-1.04) | 0.62 (0.44-0.89) | 0.89 (0.73-1.09) | 0.64 (0.47-0.89) | 0.84 (0.70-1.02) | 0.68 (0.44-1.06) | 0.78 (0.62-0.97) | 0.77 (0.60-1.00) |
| P_trend_ | 0.0081 | 0.1105 | 0.0087 | 0.2585 | 0.0078 | 0.0793 | 0.0936 | 0.0283 | 0.0450 |
| Per 1-SD | 0.92 (0.86-0.99) | 0.95 (0.88-1.03) | 0.84 (0.72-0.98) | 0.97 (0.89-1.05) | 0.83 (0.72-0.95) | 0.94 (0.87-1.02) | 0.88 (0.73-1.06) | 0.93 (0.85-1.02) | 0.88 (0.79-0.98) |
| P-value | 0.0220 | 0.2460 | 0.0247 | 0.4296 | 0.0086 | 0.1561 | 0.1698 | 0.1463 | 0.0224 |
| P_difference_ | NA | 0.10 | | 0.03 | | 0.42 | | 0.53 | |
| **Females** |  |  |  |  |  |  |  |  |  |
| No cases | 1,130 | 685 | 348 | 627 | 402 | 712 | 275 | 680 | 311 |
| -1.58 to -0.591 | Reference | Reference | Reference | Reference | Reference | Reference | Reference | Reference | Reference |
| -0.591 to 0.2 | 1.02 (0.86-1.21) | 1.12 (0.91-1.37) | 0.83 (0.63-1.09) | 1.08 (0.88-1.34) | 1.00 (0.78-1.29) | 1.11 (0.91-1.36) | 0.87 (0.64-1.18) | 1.00 (0.82-1.23) | 1.00 (0.75-1.032) |
| 0.2 to 3.03 | 1.13 (0.95-1.35) | 1.15 (0.93-1.42) | 0.97 (0.73-1.28) | 1.24 (1.00-1.54) | 1.01 (0.78-1.32) | 1.17 (0.95-1.44) | 0.96 (0.71-1.31) | 1.13 (0.92-1.39) | 1.01 (0.76-1.36) |
| P_trend_ | 0.1631 | 0.1768 | 0.7191 | 0.0540 | 0.9130 | 0.1275 | 0.7435 | 0.2681 | 0.9303 |
| Per 1-SD | 1.01 (0.94-1.09) | 1.01 (0.92-1.10) | 0.97 (0.85-1.09) | 1.03 (0.94-1.13) | 0.98 (0.88-1.10) | 1.03 (0.94-1.12) | 0.94 (0.82-1.08) | 1.02 (0.93-1.11) | 0.97 (0.86-1.11) |
| P-value | 0.7287 | 0.8791 | 0.5938 | 0.5425 | 0.7832 | 0.5731 | 0.4011 | 0.7372 | 0.6883 |
| P_difference_ | NA, | 0.66 | | 0.71 | | 0.35 | | 0.52 | |

^a^ Controls are used as the reference for all odds ratios and relative risk ratio. CI, confidence interval; CIMP, CpG island methylator phenotype; CRC, colorectal cancer; MSI, microsatellite instability; MSS, microsatellite stable; OR, odds ratio; RRR, relative risk ratio.

^b^ Odds and relative risk ratios were adjusted for study population, age, sex (when not stratified), smoking status, alcohol consumption, education, and red meat intake.

^c^ Case-only analyses used to calculate *P*_difference_.

**Table S8**. Association between physical activity, proximal colon cancer, and its molecular subtypes stratified by study design ^a, b, c^.

|  |  | **Microsatellite instability** | | **CpG island methylator phenotype** | | ***BRAF*** | | ***KRAS*** | |
| --- | --- | --- | --- | --- | --- | --- | --- | --- | --- |
| **MET hr/wk.** | **Proximal colon cancer**  **OR (95%CI)** | **MSS/MSI-L**  **RRR (95%CI)** | **MSI-H**  **RRR (95%CI)** | **CIMP-low/negative**  **RRR (95%CI)** | **CIMP-high**  **RRR (95%CI)** | ***BRAF*-wild type**  **RRR (95%CI)** | ***BRAF*-mutated**  **RRR (95%CI)** | ***KRAS*-wild type**  **RRR (95%CI)** | ***KRAS*-mutated**  **RRR (95%CI)** |
| **Cohort studies** | | | | | | | | | |
| **Sex-combined** |  |  |  |  |  |  |  |  |  |
| No cases | 966 | 578 | 258 | 554 | 345 | 622 | 220 | 481 | 32 |
| -1.45 to -0.692 | Reference | Reference | Reference | Reference | Reference | Reference | Reference | Reference | Reference |
| -0.691 to 0.212 | 0.97 (0.80-1.16) | 0.98 (0.78-1.23) | 0.95 (0.70-1.30) | 0.93 (0.74-1.18) | 1.02 (0.78-1.35) | 0.98 (0.78-1.22) | 0.97 (0.69-1.34) | 1.00 (0.78-1.27) | 0.88 (0.66-1.18) |
| 0.211 to 3.025 | 0.88 (0.73-1.07) | 0.91 (0.72-1.14) | 0.73 (0.52-1.02) | 0.97 (0.77-1.22) | 0.80 (0.60-1.07) | 0.92 (0.74-1.15) | 0.74 (0.52-1.05) | 0.87 (0.68-1.11) | 0.89 (0.69-1.19) |
| P_trend_ | 0.2078 | 0.4045 | 0.0697 | 0.7749 | 0.1409 | 0.4569 | 0.1034 | 0.2811 | 0.4153 |
| Per 1-SD | 0.94 (0.87-1.02) | 0.96 (0.87-1.05) | 0.87 (0.76-1.01) | 0.97 (0.87-1.07) | 0.90 (0.80-1.02) | 0.95 (0.87-1.04) | 0.89 (0.77-1.04) | 0.94 (0.85-1.05) | 0.94 (0.83-1.06) |
| P-value | 0.1346 | 0.3617 | 0.0637 | 0.5289 | 0.1080 | 0.2968 | 0.1367 | 0.2828 | 0.3035 |
| P_difference_ | NA | 0.18 | | 0.25 | | 0.32 | | 0.95 | |
| **Males** |  |  |  |  |  |  |  |  |  |
| No cases | 437 | 292 | 88 | 274 | 119 | 321 | 64 | 194 | 163 |
| -1.45 to -0.692 | Reference | Reference | Reference | Reference | Reference | Reference | Reference | Reference | Reference |
| -0.691 to 0.212 | 0.81 (0.61-1.08) | 0.83 (0.60-1.15) | 0.84 (0.50-1.43) | 0.80 (0.57-1.11) | 0.81 (0.50-1.29) | 0.80 (0.58-1.09) | 0.79 (0.42-1.48) | 0.84 (0.57-1.22) | 0.70 (0.46-1.07) |
| 0.211 to 3.025 | 0.72 (0.54-0.95) | 0.75 (0.54-1.05) | 0.59 (0.33-1.03) | 0.76 (0.54-1.07) | 0.65 (0.40-1.06) | 0.71 (0.52-0.98) | 0.69 (0.36-1.31) | 0.65 (0.43-0.96) | 0.77 7 (0.51-1.16) |
| P_trend_ | 0.0223 | 0.0933 | 0.0658 | 0.1193 | 0.0844 | 0.0373 | 0.2544 | 0.0308 | 0.21155 |
| Per 1-SD | 0.88 (0.78-0.99) | 0.90 (0.79-1.04) | 0.80 (0.63-1.02) | 0.90 (0.78-1.04) | 0.83 (0.67-1.02) | 0.87 (0.76-0.99) | 0.92 (0.71-1.20) | 0.86 (0.73-1.02) | 0.87 (0.73-1.04) |
| P-value | 0.0341 | 0.1461 | 0.0776 | 0.1402 | 0.0812 | 0.0369 | 0.5276 | 0.0797 | 0.1379 |
| P_difference_ | NA | 0.30 | | 0.42 | | 0.80 | | 0.88 | |
| **Females** |  |  |  |  |  |  |  |  |  |
| No cases | 529 | 286 | 170 | 280 | 226 | 301 | 156 | 287 | 150 |
| -1.45 to -0.692 | Reference | Reference | Reference | Reference | Reference | Reference | Reference | Reference | Reference |
| -0.691 to 0.212 | 1.12 (0.87-1.43) | 1.15 (0.84-1.58) | 1.03 (0.70-1.52) | 1.08 (0.78-1.49) | 1.19 (0.85-1.67) | 1.20 (0.88-1.63) | 1.08 (0.73-1.60) | 1.15 (0.84-1.58) | 1.09 (0.72-1.64) |
| 0.211 to 3.025 | 1.03 (0.80-1.34) | 1.05 (0.76-1.45) | 0.83 (0.55-1.25) | 1.19 (0.87-1.64) | 0.89 (0.61-1.28) | 1.16 (0.84-1.59) | 0.76 (0.49-1-.17) | 1.05 (0.76-1.46) | 0.98 (0.64-1.50) |
| P_trend_ | 0.7559 | 0.7134 | 0.4116 | 0.2811 | 0.5966 | 0.3392 | 0.2591 | 0.7034 | 0.9667 |
| Per 1-SD | 0.99 (0.89-1.10) | 1.00 (0.87-1.14) | 0.92 (0.77-1.09) | 1.03 (0.90-1.18) | 0.94 (0.81--1.10) | 1.03 (0..91-1.18) | 0.88 (0.73-1.06) | 1.00 (0.87-1.14) | 0.99 (0.83-1.18) |
| P-value | 0.8035 | 0.9495 | 0.3377 | 0.6557 | 0.4285 | 0.6244 | 0.1724 | 0.9646 | 0.8798 |
| P_difference_ | NA | 0.45 | | 0.46 | | 0.15 | | 0.82 | |
| **Case-controls studies** | | | | | | | | | |
| **Sex-combined** |  |  |  |  |  |  |  |  |  |
| No cases | 1,247 | 877 | 302 | 798 | 312 | 900 | 192 | 763 | 394 |
| -1.58 to -0.569 | Reference | Reference | Reference | Reference | Reference | Reference | Reference | Reference | Reference |
| -0.568 to 0.182 | 0.89 (0.76-1.04) | 0.95 (0.80-1.14) | 0.73 (0.54-0.97) | 0.96 (0.80-1.17) | 0.81 (0.61-1.07) | 0.91 (0.76-1.10) | 0.81 (0.57-1.16) | 0.89 (0.73-1.07) | 0.82 (0.64-1.06) |
| 0.181 to 2.73 | 1.01 (0.86-1.18) | 1.05 (0.88-1.27) | 0.89 (0.66-1.18) | 1.10 (0.91-1.33) | 0.91 (0.68-1.21) | 1.04 (0.87-1.25) | 1.00 (0.70-1.43) | 1.02 (0.84-1.24) | 0.85 (0.66-1.10) |
| P_trend_ | 0.9614 | 0.5934 | 0.3409 | 0.3317 | 0.47311 | 0.6913 | 0.9436 | 0.8719 | 0.2088 |
| Per 1-SD | 0.98 (0.92-1.05) | 1.00 (0.92-1.08) | 0.95 (0.83-1.08) | 1.02 (0.94-1.10) | 0.94 (0.82-1.06) | 1.00 (0.93-1.08) | 0.96 (0.81-1.12) | 1.00 (0.92-1.09) | 0.90 (0.80-1.01) |
| P-value | 0.6369 | 0.9380 | 0.4145 | 0.7031 | 0.3080 | 0.9514 | 0.5845 | 0.9770 | 0.0681 |
| P_difference_ | NA | 0.36 | | 0.18 | | 0.57 | | 0.22 | |
| **Males** |  |  |  |  |  |  |  |  |  |
| No cases | 646 | 478 | 124 | 451 | 136 | 489 | 73 | 370 | 2330 |
| -1.58 to -0.569 | Reference | Reference | Reference | Reference | Reference | Reference | Reference | Reference | Reference |
| -0.568 to 0.182 | 0.83 (0.67-1.03) | 0.84 (0.65-1.08) | 0.79 (0.51-1.22) | 0.87 (0.67-1.12) | 0.78 (0.52-1.19) | 0.80 (0.62-1.02) | 1.05 (0.61-1.81) | 0.85 (0.64-1.11) | 0.75 (0.54-1.05) |
| 0.181 to 2.73 | 0.83 (0.66-1.03) | 0.90 (0.70-1.15) | 0.64 (0.41-1.01) | 0.95 (0.74-1.22) | 0.64 (0.41-0.98) | 0.91 (0.72-1.16) | 0.63 (0.34-1.18) | 0.85 (0.64-1.11) | 0.74 (0.53-1.03) |
| P_trend_ | 0.0825 | 0.3817 | 0.0560 | 0.6936 | 0.0412 | 0.4541 | 0.1732 | 0.2248 | 0.0703 |
| Per 1-SD | 0.94 (0.86-1.02) | 0.98 (0.88-1.08) | 0.86 (0.71-1.05) | 1.00 (0.90-1.10) | 0.82 (0.68-1.00) | 0.98 (0.89-1.08) | 0.83 (0.64-1.08) | 0.97 (0.86-1.08) | 0.87 (0.75-1.00) |
| P-value | 0.1596 | 0.6245 | 0.1329 | 0.9713 | 0.0472 | 0.7459 | 0.1612 | 0.5389 | 0.0493 |
| P_difference_ | NA | 0.22 | | 0.03 | | 0.21 | | 0.33 | |
| **Females** |  |  |  |  |  |  |  |  |  |
| No cases | 601 | 399 | 178 | 347 | 176 | 411 | 119 | 393 | 161 |
| -1.58 to -0.569 | Reference | Reference | Reference | Reference | Reference | Reference | Reference | Reference | Reference |
| -0.568 to 0.182 | 0.97 (0.77-1.22) | 1.12 (0.86-1.47) | 0.68 (0.46-1.01) | 1.11 (0.83-1.48) | 0.84 (0.57-1.24) | 1.08 (0.83-1.41) | 0.67 (0.41-1.08) | 0.94 (0.72-1.24) | 0.94 (0.64-1.39) |
| 0.181 to 2.73 | 1.27 (1.00-1.62) | 1.29 (0.97-1.71) | 1.14 (0.78-1.66) | 1.33 (0.99-1.79) | 1.23 (0.84-1.82) | 1.23 (0.93-1.62) | 1.27 (0.81-1.99) | 1.25 (0.95-1.66) | 1.06 (0.70-1.60) |
| P_trend_ | 0.0542 | 0.0788 | 0.6220 | 0.0627 | 0.3321 | 0.1477 | 0.3556 | 0.1304 | 0.8018 |
| Per 1-SD | 1.05 (0.94-1.17) | 1.03 (0.91-1.17) | 1.03 (0.86-1.23) | 1.04 (0.91-1.18) | 1.05 (0.88-1.26) | 1.03 (0.91-1.17) | 1.04 (0.84-1.29) | 1.05 (0.92-1.19) | 0.96 (0.80-1.16) |
| P-value | 0.3627 | 0.6560 | 0.7349 | 0.5709 | 0.5583 | 0.6314 | 0.6930 | 0.4631 | 0.7054 |
| P_difference_ | NA | 0.94 | | 0.68 | | 0.73 | | 0.46 | |

^a^ Controls are used as the reference for all odds ratios. CI, confidence interval; CIMP, CpG island methylator phenotype; CRC, colorectal cancer; MSI, microsatellite instability; MSS, microsatellite stable; OR, odds ratio.

^b^ Odds ratios are adjusted for study population, age, sex (when not stratified), smoking status, alcohol consumption, education, and red meat intake.

^c^ Case-only analyses used to calculate *P*_difference_.

**Table S9**. Association between physical activity, distal colon cancer, and its molecular subtypes^a, b, c^.

|  |  | **Microsatellite instability** | | **CpG island methylator phenotype** | | ***BRAF*** | | ***KRAS*** | |
| --- | --- | --- | --- | --- | --- | --- | --- | --- | --- |
| **MET hr/wk.** | **Distal colon cancer**  **OR (95%CI)** | **MSS/MSI-L**  **RRR (95%CI)** | **MSI-H**  **RRR (95%CI)** | **CIMP-low/negative**  **RRR (95%CI)** | **CIMP-high**  **RRR (95%CI)** | ***BRAF*-wild type**  **RRR (95%CI)** | ***BRAF*-mutated**  **RRR (95%CI)** | ***KRAS*-wild type**  **RRR (95%CI)** | ***KRAS*-mutated**  **RRR (95%CI)** |
| **Sex-combined** | |  |  |  |  |  |  |  |  |
| No cases | 1,608 | 1,420 | 73 | 1,339 | 110 | 1,378 | 55 | 991 | 442 |
| -1.58 to -0.591 | Reference | Reference | Reference | Reference | Reference | Reference | Reference | Reference | Reference |
| -0.591 to 0.2 | 0.79 (0.69-0.90) | 0.75 (0.65-0.87) | 1.01 (0.58-1.76) | 0.77 (0.66-0.89) | 0.99 (0.63-1.54) | 0.78 (0.67-0.90) | 1.16 (0.62-2.17) | 0.75 (0.64-0.89) | 0.82 (0.65-1.04) |
| 0.2 to 3.03 | 0.82 (0.71-0.94) | 0.82 (0.71-0.95) | 0.97 (0.54-1.73) | 0.82 (0.71-0.95) | 0.81 (0.50-1.32) | 0.80 (0.69-0.93) | 0.76 (0.38-1.54) | 0.73 (0.62-0.86) | 0.87 (0.69-1.11) |
| P_trend_ | 0.003 | 0.005 | 0.9187 | 0.0064 | 0.4194 | 0.002 | 0.4716 | 0.0002 | 0.2439 |
| Per 1-SD | 0.94 (0.89-1.00) | 0.95 (0.89-1.01) | 0.96 (0.75-1.23) | 0.95 (0.89-1.01) | 0.95 (0.78-1.16) | 0.93 (0.88-0.99) | 0.93 (0.70-1.24) | 0.89 (0.83-0.96) | 1.00 (0.91-1.10) |
| P-value | 0.0546 | 0.0976 | 0.7450 | 0.1042 | 0.6220 | 0.029 | 0.6317 | 0.0024 | 0.994 |
| P_difference_ | NA | 0.97 | | 0.98 | | 0.82 | | 0.06 | |
| **Males** |  |  |  |  |  |  |  |  |  |
| No cases | 918 | 799 | 43 | 765 | 58 | 797 | 27 | 569 | 250 |
| -1.58 to -0.591 | Reference | Reference | Reference | Reference | Reference | Reference | Reference | Reference | Reference |
| -0.591 to 0.2 | 0.78 (0.65-0.94) | 0.72 (0.59-0.88) | 0.96 (0.47-1.98) | 0.75 (0.62-0.91) | 0.74 (0.40-1.39) | 0.75 (0.62-0.91) | 0.82 (0.33-2.01) | 0.77 (0.62-0.99) | 0.74 (0.53-1.02) |
| 0.2 to 3.03 | 0.77 (0.64-0.92) | 0.78 (0.64-0.95) | 0.79 (0.36-1.71) | 0.78 (0.65-0.95) | 0.64 (0.34-1.23) | 0.76 (0.62-0.92) | 0.64 (0.24-1.69) | 0.70 (0.56-0.88) | 0.85 (0.62-1.16) |
| P_trend_ | 0.005 | 0.0107 | 0.5637 | 0.0143 | 0.1814 | 0.0044 | 0.3688 | 0.002 | 0.2873 |
| Per 1-SD | 0.92 (0.85-0.99) | 0.93 (0.83-1.00) | 0.94 (0.69-1.29) | 0.94 (0.87-1.02) | 0.84 (0.63-1.11) | 0.91 (0.84-0.99) | 0.86 (0.56-1.30) | 0.87 (0.79-0.96) | 1.00 (0.88-1.14) |
| P-value | 0.033 | 0.0589 | 0.7121 | 0.1246 | 0.2133 | 0.0276 | 0.4655 | 0.0044 | 0.9725 |
| P_difference_ | NA | 0.47 | | 0.34 | | 0.83 | | 0.08 | |
| **Females** |  |  |  |  |  |  |  |  |  |
| No cases | 690 | 621 | 30 | 574 | 52 | 581 | 28 | 422 | 192 |
| -1.58 to -0.591 | Reference | Reference | Reference | Reference | Reference | Reference | Reference | Reference | Reference |
| -0.591 to 0.2 | 0.78 (0.65-0.94) | 0.80 (0.64-0.99) | 1.11 (0.46-2.71) | 0.79 (0.63-0.98) | 1.34 (0.70-2.58) | 0.81 (0.65-1.01) | 1.72 (0.71-4.22) | 0.74 (0.58-0.95) | 0.94 (0.66-1.33) |
| 0.2 to 3.03 | 0.88 (0.72-1.09) | 0.88 (0.70-1.09) | 1.34 (0.55-3.28) | 0.87 (0.70-1.09) | 1.10 (0.54-2.77) | 0.86 (0.69-1.08) | 0.95 (0.34-2.68) | 0.78 (0.60-1.01) | 0.90 (0.62-1.30) |
| P_trend_ | 0.1959 | 0.1812 | 0.5262 | 0.1871 | 0.7429 | 0.1572 | 0.9626 | 0.0387 | 0.5619 |
| Per 1-SD | 0.98 (0.90-1.07) | 0.98 (0.89-1.08) | 1.00 (0.68-1.47) | 0.97 (0.87-1.07) | 1.12 (0.84-1.49) | 0.96 (0.87-1.06) | 1.04 (0.71-1.52) | 0.93 (0.83-1.04) | 0.99 (0.85-1.16) |
| P-value | 0.6863 | 0.7357 | 0.995 | 0.4907 | 0.4305 | 0.4497 | 0.8577 | 0.2123 | 0.9313 |
| P_difference_ | NA | 0.72 | | 0.23 | | 0.27 | | 0.97 | |

^a^ Controls are used as the reference for all odds ratios and relative risk ratio. CI, confidence interval; CIMP, CpG island methylator phenotype; CRC, colorectal cancer; MSI, microsatellite instability; MSS, microsatellite stable; OR, odds ratio; RRR, relative risk ratio.

^b^ Odds and relative risk ratios were adjusted for study, age, sex (when not stratified), smoking status, alcohol consumption, education, and red meat intake.

^c^ Case-only analyses used to calculate *P*_difference_.

**Table S10**. Association between physical activity, distal colon cancer, and its molecular subtypes stratified by study design ^a, b, c^.

|  |  | **Microsatellite instability** | | **CpG island methylator phenotype** | | ***BRAF*** | | ***KRAS*** | |
| --- | --- | --- | --- | --- | --- | --- | --- | --- | --- |
| **MET hr/wk.** | **Distal colon cancer**  **OR (95%CI)** | **MSS/MSI-L**  **RRR (95%CI)** | **MSI-H**  **RRR (95%CI)** | **CIMP-low/negative**  **RRR (95%CI)** | **CIMP-high**  **RRR (95%CI)** | ***BRAF*-wild type**  **RRR (95%CI)** | ***BRAF*-mutated**  **RRR (95%CI)** | ***KRAS*-wild type**  **RRR (95%CI)** | ***KRAS*-mutated**  **RRR (95%CI)** |
| **Cohort studies** | | | | | | | | | |
| **Sex-combined** |  |  |  |  |  |  |  |  |  |
| No cases | 556 | 474 | 31 | 461 | 44 | 481 | 32 | 315 | 164 |
| -1.45 to -0.692 | Reference | Reference | Reference | Reference | Reference | Reference | Reference | Reference | Reference |
| -0.691 to 0.212 | 0.82 (0.66-1.03) | 0.76 (0.60-0.97) | 1.30 (0.56-3.50) | 0.78 (0.61-0.99) | 1.39 (0.69-2.80) | 0.78 (0.61-0.99) | 1.56 (0.66-3.68) | 0.87 (0.65-1.15) | 0.61 (0.41-0.92) |
| 0.211 to 3.025 | 0.67 (0.53-0.85) | 0.68 (0.53-0.88) | 0.90 (0.34-2.34) | 0.68 (0.53-0.88) | 0.71 (0.31-1.62) | 0.65 (0.51-0.84) | 0.98 (0.38-2.51) | 0.68 (0.50-0.92) | 0.63 (0.43-0.94) |
| P_trend_ | 0.001 | 0.0022 | 0.8566 | 0.0027 | 0.4638 | 0.0008 | 0.9468 | 0.0119 | 0.016 |
| Per 1-SD | 0.84 (0.76-0.93) | 0.85 (0.76-0.95) | 0.91 (0.62-1.33) | 0.86 (0.77-0.96) | 0.90 (0.65-1.23) | 0.82 (0.73-0.92) | 1.04 (0.73-1.47) | 0.83 (0.73-0.95) | 0.84 (0.70-1.00) |
| P-value | 0.001 | 0.0033 | 0.6192 | 0.0065 | 0.5062 | 0.0005 | 0.8448 | 0.0064 | 0.0539 |
| P_difference_ | NA | 0.74 | | 0.80 | | 0.17 | | 0.83 | |
| **Males** |  |  |  |  |  |  |  |  |  |
| No cases | 301 | 253 | 18 | 245 | 20 | 265 | 12 | 166 | 90 |
| -1.45 to -0.692 | Reference | Reference | Reference | Reference | Reference | Reference | Reference | Reference | Reference |
| -0.691 to 0.212 | 0.73 (0.53-1.01) | 0.67 (0.47-0.94) | 0.79 (0.27-2.30) | 0.71 (0.50-1.00) | 0.85 (0.31-2.30) | 0.69 (0.49-0.96) | 1.06 (0.23-4.96) | 0.76 (0.51-1.12) | 0.51 (0.29-0.89) |
| 0.211 to 3.025 | 0.53 (0.38-0.74) | 0.56 (0.39-0.79) | 0.39 (0.10-1.54) | 0.55 (0.38-0.79) | 0.29 (0.08-1.14) | 0.50 (0.35-0.72) | 1.44 (0.32-6.42) | 0.50 (0.33-0.77) | 0.58 (0.34-0.98) |
| P_trend_ | 0.0002 | 0.0011 | 0.1847 | 0.0011 | 0.0801 | 0.0001 | 0.6185 | 0.0017 | 0.0345 |
| Per 1-SD | 0.78 (0.67-0.90) | 0.78 (0.67-0.91) | 0.89 (0.53-1.08) | 0.80 (0.69-0.94) | 0.76 (0.45-1.27) | 0.75 (0.64-0.87) | 1.32 (0.76-2.29) | 0.71 (0.59-0.87) | 0.88 (0.70-1.11) |
| P-value | 0.0008 | 0.0021 | 0.6615 | 0.0056 | 0.2892 | 0.0003 | 0.3160 | 0.0007 | 0.2837 |
| P_difference_ | NA | 0.69 | | 0.76 | | 0.06 | | 0.19 | |
| **Females** |  |  |  |  |  |  |  |  |  |
| No cases | 255 | 221 | 13 | 216 | 24 | 216 | 20 | 149 | 74 |
| -1.45 to -0.692 | Reference | Reference | Reference | Reference | Reference | Reference | Reference | Reference | Reference |
| -0.691 to 0.212 | 0.95 (0.68-1.32) | 0.89 (0.62-1.26) | 3.74 (0.74-19.01) | 0.87 (0.61-1.25) | 2.38 (0.86-6.62) | 0.92 (0.65-1.32) | 1.92 (0.68-5.42) | 1.02 (0.67-1.54) | 0.81 (0.45-1.44) |
| 0.211 to 3.025 | 0.87 (0.62-1.22) | 0.84 (0.59-1.21) | 3.34 (0.63-17.82) | 0.86 (0.60-1.23) | 1.56 (0.51-4.77) | 0.89 (0.62-1.28) | 0.69 (0.19-2.53) | 0.93 (0.61-1.42) | 0.70 (0.39-1.26) |
| P_trend_ | 0.4266 | 0.3387 | 0.1692 | 0.3906 | 0.4196 | 0.5149 | 0.6541 | 0.7497 | 0.2190 |
| Per 1-SD | 0.91 (0.78-1.05) | 0.91 (0.78-1.07) | 0.98 (0.55-1.76) | 0.91 (0.78-1.07) | 1.07 (0.71-1.62) | 0.91 (0.77-1.06) | 0.91 (0.56-1.45) | 0.96 (0.80-1.16) | 0.78 (0.60-1.03) |
| P-value | 0.2152 | 0.2653 | 0.9497 | 0.2604 | 0.7327 | 0.2266 | 0.6792 | 0.6842 | 0.0769 |
| P_difference_ | NA | 0.49 | | 0.31 | | 0.47 | | 0.30 | |
| **Case-controls studies** | | | | | | | | | |
| **Sex-combined** |  |  |  |  |  |  |  |  |  |
| No cases | 1,052 | 946 | 42 | 878 | 66 | 897 | 23 | 676 | 278 |
| -1.58 to -0.569 | Reference | Reference | Reference | Reference | Reference | Reference | Reference | Reference | Reference |
| -0.568 to 0.182 | 0.77 (0.64-0.91) | 0.75 (0.63-0.90) | 0.77 (0.36-1.64) | 0.76 (0.63-0.92) | 0.78 (0.43-1.42) | 0.77 (0.65-0.93) | 0.84 (0.33-2.15) | 0.71 (0.58-0.86) | 0.97 (0.72-1.32) |
| 0.181 to 2.73 | 0.91 (0.77-1.08) | 0.91 (0.77-1.09) | 1.01 (0.48-2.12) | 0.92 (0.77-1.10) | 0.88 (0.48-1.60) | 0.90 (0.75-1.08) | 0.58 (0.20-1.75) | 0.77 (0.63-0.94) | 1.06 (0.78-1.43) |
| P_trend_ | 0.2568 | 0.2729 | 0.9908 | 0.3133 | 0.6534 | 0.2203 | 0.3451 | 0.0082 | 0.7182 |
| Per 1-SD | 1.00 (0.93-1.08) | 1.01 (0.93-1.08) | 1.00 (0.73-1.36) | 1.00 (0.93-1.08) | 0.98 (0.76-1.26) | 1.00 (0.93-1.08) | 0.78 (0.42-1.27) | 0.93 (0.85-1.01) | 1.09 (0.97-1.23) |
| P-value | 0.9127 | 0.8558 | 0.9779 | 0.9094 | 0.8688 | 0.9644 | 0.3223 | 0.1026 | 0.1594 |
| P_difference_ | NA | 0.94 | | 0.81 | | 0.31 | | 0.04 | |
| **Males** |  |  |  |  |  |  |  |  |  |
| No cases | 617 | 546 | 25 | 520 | 38 | 532 | 15 | 403 | 160 |
| -1.58 to -0.569 | Reference | Reference | Reference | Reference | Reference | Reference | Reference | Reference | Reference |
| -0.568 to 0.182 | 0.80 (0.64-1.01) | 0.75 (0.59-0.95) | 1.06 (0.39-2.89) | 0.77 (0.60-0.98) | 0.71 (0.31-1.60) | 0.78 (0.61-1.00) | 0.70 (0.22-2.49) | 0.77 (0.59-1.01) | 0.91 (0.61-1.37) |
| 0.181 to 2.73 | 0.91 (0.73-1.14) | 0.91 (0.72-1.14) | 1.18 (0.44-3.18) | 0.92 (0.73-1.17) | 0.91 (0.43-1.97) | 0.92 (0.73-1.16) | 0.30 (0.06-1.46) | 0.81 (0.62-1.06) | 1.03 (0.69-1.53) |
| P_trend_ | 0.4210 | 0.4113 | 0.7330 | 0.5165 | 0.8074 | 0.4686 | 0.1321 | 0.1168 | 0.8799 |
| Per 1-SD | 0.99 (0.90-1.08) | 0.99 (0.90-1.09) | 0.97 (0.65-1.44) | 1.00 (0.91-1.10) | 0.89 (0.63-1.25) | 0.99 (0.90-1.09) | 0.52 (0.24-1.11) | 0.94 (0.84-1.05) | 1.06 (0.90-1.23) |
| P-value | 0.7475 | 0.8523 | 0.8608 | 0.9460 | 0.4910 | 0.8962 | 0.0917 | 0.2579 | 0.4890 |
| P_difference_ | NA | 0.87 | | 0.42 | | 0.07 | | 0.25 | |
| **Females** |  |  |  |  |  |  |  |  |  |
| No cases | 435 | 400 | 17 | 358 | 28 | 365 | 8 | 273 | 118 |
| -1.58 to -0.569 | Reference | Reference | Reference | Reference | Reference | Reference | Reference | Reference | Reference |
| -0.568 to 0.182 | 0.73 (0.56-0.94) | 0.76 (0.58-1.00) | 0.52 (0.15-1.77) | 0.76 (0.57-1.00) | 0.87 (0.36-2.09) | 0.77 (0.58-1.01) | 1.37 (0.23-8.34) | 0.63 (0.46-0.86) | 1.06 (0.67-1.67) |
| 0.181 to 2.73 | 0.92 (0.71-1.21) | 0.92 (0.70-1.22) | 0.84 (0.27-2.67) | 0.91 (0.68-1.22) | 0.82 (0.31-2.17) | 0.88 (0.66-1.17) | 1.78 (0.29-10.96) | 0.73 (0.53-1.02) | 1.08 (0.67-1.75) |
| P_trend_ | 0.4481 | 0.4808 | 0.6931 | 0.4576 | 0.6876 | 0.2995 | 0.5347 | 0.0357 | 0.7399 |
| Per 1-SD | 1.04 (0.96-1.16) | 1.04 (0.92-1.17) | 1.02 (0.61-1.71) | 1.01 (0.89-1.15) | 1.14 (0.77-1.69) | 1.01 (0.89-1.14) | 1.34 (0.69-2.61) | 0.92 (0.80-1.07) | 1.14 (0.94-1.38) |
| P-value | 0.5332 | 0.5701 | 0.9444 | 0.8775 | 0.5080 | 0.9107 | 0.3878 | 0.2772 | 0.1752 |
| P_difference_ | NA | 0.96 | | 0.54 | | 0.43 | | 0.08 | |

^a^ Controls are used as the reference for all odds ratios. CI, confidence interval; CIMP, CpG island methylator phenotype; CRC, colorectal cancer; MSI, microsatellite instability; MSS, microsatellite stable; OR, odds ratio.

^b^ Odds ratios are adjusted for study, age, sex (when not stratified), smoking status, alcohol consumption, education, and red meat intake.

^c^ Case-only analyses used to calculate *P*_difference_.

**Table S11**. Association between physical activity, colon (proximal and distal) cancer, and its molecular subtypes^a, b, c^.

|  |  | **Microsatellite instability** | | **CpG island methylator phenotype** | | ***BRAF*** | | ***KRAS*** | |
| --- | --- | --- | --- | --- | --- | --- | --- | --- | --- |
| **MET hr/wk.** | **Distal colon cancer**  **OR (95%CI)** | **MSS/MSI-L**  **RRR (95%CI)** | **MSI-H**  **RRR (95%CI)** | **CIMP-low/negative**  **RRR (95%CI)** | **CIMP-high**  **RRR (95%CI)** | ***BRAF*-wild type**  **RRR (95%CI)** | ***BRAF*-mutated**  **RRR (95%CI)** | ***KRAS*-wild type**  **RRR (95%CI)** | ***KRAS*-mutated**  **RRR (95%CI)** |
| **Sex-combined** | |  |  |  |  |  |  |  |  |
| No cases | 3,851 | 2,901 | 636 | 2,709 | 769 | 2,923 | 469 | 2,251 | 1,158 |
| -1.58 to -0.591 | Reference | Reference | Reference | Reference | Reference | Reference | Reference | Reference | Reference |
| -0.591 to 0.2 | 0.86 (0.78-0.95) | 0.85 (0.77-0.95) | 0.85 (0.70-1.04) | 0.85 (0.76-0.96) | 0.92 (0.76-1.10) | 0.86 (0.77-0.96) | 0.93 (0.74-1.16) | 0.85 (0.75-0.95) | 0.84 (0.72-0.98) |
| 0.2 to 3.03 | 0.90 (0.81-0.99) | 0.91 (0.81-1.01) | 0.83 (0.67-1.02) | 0.93 (0.83-1.04) | 0.83 (0.69-1.01) | 0.90 (0.80-1.00) | 0.84 (0.66-1.06) | 0.85 (0.75-.96) | 0.88 (0.75-1.02) |
| P_trend_ | 0.028 | 0.077 | 0.0675 | 0.1859 | 0.0615 | 0.0432 | 0.1461 | 0.0072 | 0.0869 |
| Per 1-SD | 0.96 (0.92-1.00) | 0.97 (0.92-1.01) | 0.92 (0.84-1.00) | 0.97 (0.93-1.02) | 0.92 (0.85-1.00) | 0.96 (0.92-1.00) | 0.92 (0.83-1.02) | 0.94 (0.89-0.99) | 0.95 (0.89-1.02) |
| P-value | 0.043 | 0.1532 | 0.0583 | 0.2631 | 0.0478 | 0.0728 | 0.1147 | 0.0168 | 0.1364 |
| P_difference_ | NA | 0.22 | | 0.18 | | 0.45 | | 0.55 | |
| **Males** |  |  |  |  |  |  |  |  |  |
| No cases | 2,024 | 1,588 | 258 | 1,502 | 315 | 1,624 | 166 | 1,145 | 654 |
| -1.58 to -0.591 | Reference | Reference | Reference | Reference | Reference | Reference | Reference | Reference | Reference |
| -0.591 to 0.2 | 0.81 (0.70-0.93) | 0.78 (0.67-0.91) | 0.87 (0.64-1.18) | 0.80 (0.68-0.93) | 0.79 (0.59-1.04) | 0.78 (0.67-0.91) | 0.95 (0.65-1.37) | 0.81 (0.68-0.96) | 0.75 (0.61-0.92) |
| 0.2 to 3.03 | 0.78 (0.68-0.90) | 0.82 (0.70-0.95) | 0.65 (0.47-0.91) | 0.83 (0.72-0.97) | 0.64 (0.48-0.64) | 0.80 (0.69-0.92) | 0.67 (0.45-1.01) | 0.74 (0.62-0.87) | 0.81 (0.66-0.99) |
| P_trend_ | 0.0006 | 0.0089 | 0.0121 | 0.0188 | 0.0033 | 0.0025 | 0.0627 | 0.0004 | 0.0395 |
| Per 1-SD | 0.92 (0.87-0.98) | 0.94 (0.89-1.00) | 0.86 (0.75-0.99) | 0.95 (0.90-1.01) | 0.83 (0.73-0.94) | 0.93 (0.87-0.99) | 0.88 (0.74-1.04) | 0.90 (0.80-0.97) | 0.93 (0.85-1.01) |
| P-value | 0.0051 | 0.0579 | 0.0333 | 0.1207 | 0.0043 | 0.0174 | 0.1230 | 0.0042 | 0.0987 |
| P_difference_ | NA | 0.17 | | 0.03 | | 0.44 | | 0.44 | |
| **Females** |  |  |  |  |  |  |  |  |  |
| No cases | 1,827 | 1,3113 | 378 | 1,207 | 454 | 1,299 | 303 | 1,1106 | 504 |
| -1.58 to -0.591 | Reference | Reference | Reference | Reference | Reference | Reference | Reference | Reference | Reference |
| -0.591 to 0.2 | 0.92 (0.80-1.06) | 0.95 (0.81-1.11) | 0.84 (0.65-1.09) | 0.92 (0.78-1.08) | 1.03 (0.81-1.03) | 0.96 (0.82-1.12) | 0.92 (0.69-1.22) | 0.89 (0.75-1.05) | 0.97 (0.77-1.21) |
| 0.2 to 3.03 | 1.04 (0.90-1.20) | 1.02 (0.86-1.20) | 1.00 (0.76-1.30) | 1.06 (0.89-1.25) | 1.02 (0.79-1.31) | 1.03 (0.87-1.22) | 0.96 (0.71-1.29) | 0.99 (0.83-1.18) | 0.97 (0.76-1.23) |
| P_trend_ | 0.6834 | 0.8604 | 0.8904 | 0.5774 | 0.8762 | 0.7618 | 0.7471 | 0.8318 | 0.7891 |
| Per 1-SD | 1.00 (0.94-1.07) | 1.00 (0.93-1.07) | 0.97 (0.87-1.06) | 1.00 (0.93-1.08) | 1.00 (0.90-1.11) | 1.00 (0.93-1.07) | 0.95 (0.83-1.09) | 0.98 (0.91-1.06) | 0.98 (0.88-1.09) |
| P-value | 0.9499 | 0.9472 | 0.6133 | 0.9950 | 0.9863 | 0.9783 | 0.4684 | 0.6863 | 0.7097 |
| P_difference_ | NA | 0.86 | | 0.73 | | 0.74 | | 0.93 | |

^a^ Controls are used as the reference for all odds ratios and relative risk ratio. CI, confidence interval; CIMP, CpG island methylator phenotype; CRC, colorectal cancer; MSI, microsatellite instability; MSS, microsatellite stable; OR, odds ratio; RRR, relative risk ratio.

^b^ Odds and relative risk ratios were adjusted for study, age, sex (when not stratified), smoking status, alcohol consumption, education, and red meat intake.

^c^ Case-only analyses used to calculate *P*_difference_.

**Table S12**. Association between physical activity, colon (proximal and distal) cancer, and its molecular subtypes stratified by study design^a, b, c^.

|  |  | **Microsatellite instability** | | **CpG island methylator phenotype** | | ***BRAF*** | | ***KRAS*** | |
| --- | --- | --- | --- | --- | --- | --- | --- | --- | --- |
| **MET hr/wk.** | **Distal colon cancer**  **OR (95%CI)** | **MSS/MSI-L**  **RRR (95%CI)** | **MSI-H**  **RRR (95%CI)** | **CIMP-low/negative**  **RRR (95%CI)** | **CIMP-high**  **RRR (95%CI)** | ***BRAF*-wild type**  **RRR (95%CI)** | ***BRAF*-mutated**  **RRR (95%CI)** | ***KRAS*-wild type**  **RRR (95%CI)** | ***KRAS*-mutated**  **RRR (95%CI)** |
| **Cohort studies** | | | | | | | | | |
| **Sex-combined** |  |  |  |  |  |  |  |  |  |
| No cases | 1,530 | 1058 | 290 | 1,021 | 390 | 1,110 | 252 | 799 | 481 |
| -1.45 to -0.692 | Reference | Reference | Reference | Reference | Reference | Reference | Reference | Reference | Reference |
| -0.691 to 0.212 | 0.91 (0.77-1.06) | 0.87 (0.73-1.05) | 0.99 (0.74-1.32) | 0.85 (0.71-1.03) | 1.06 (0.82-1.38) | 0.88 (0.74-1.06) | 1.02 (0.75-1.39) | 0.94 (0.78-1.15) | 0.77 (0.61-0.99) |
| 0.211 to 3.025 | 0.79 (0.67-0.93 | 0.80 (0.66-0.95) | 0.72 (0.53-0.99) | 0.82 (0.68-0.99) | 0.77 (0.58-1.02) | 0.79 (0.66-0.94) | 0.74 (0.53-1.04) | 0.78 (0.63-0.95) | 0.79 (0.62-1.01) |
| P_trend_ | 0.0052 | 0.0132 | 0.0519 | 0.0346 | 0.0807 | 0.0088 | 0.0946 | 0.0177 | 0.0492 |
| Per 1-SD | 0.90 (0.84-0.96) | 0.91 (0.84-0.98) | 0.75 (0.57-0.98) | 0.92 (0.85-0.99) | 0.90 (0.80-1.01) | 0.89 (0.82-0.96) | 0.90 (0.79-1.04) | 0.89 (0.82-0.98) | 0.90 (0.81-1.00) |
| P-value | 0.0027 | 0.0128 | 0.0374 | 0.0268 | 0.0654 | 0.0031 | 0.1502 | 0.0115 | 0.0593 |
| P_difference_ | NA | 0.36 | | 0.55 | | 0.94 | | 0.68 | |
| **Males** |  |  |  |  |  |  |  |  |  |
| No cases | 746 | 551 | 107 | 525 | 140 | 593 | 76 | 363 | 257 |
| -1.45 to -0.692 | Reference | Reference | Reference | Reference | Reference | Reference | Reference | Reference | Reference |
| -0.691 to 0.212 | 0.77 (0.67-0.98) | 0.75 (0.57-0.97) | 0.86 (0.54-1.38) | 0.75 (0.57-0.97) | 0.82 (0.54-1.27) | 0.74 (0.58-0.96) | 0.85 (0.47-1.51) | 0.80 (0.60-1.08) | 0.62 (0.44-0.88) |
| 0.211 to 3.025 | 0.63 (0.50-0.80) | 0.66 (0.50-0.85) | 0.54 (0.32-0.92) | 0.65 (0.50-0.85) | 0.59 (0.37-0.93) | 0.60 (0.47-0.78) | 0.78 (0.43-1.40) | 0.57 (0.42-0.78) | 0.70 (0.50-0.98) |
| P_trend_ | 0.0002 | 0.0017 | 0.0242 | 0.0017 | 0.0244 | 0.0001 | 0.4090 | 0.0005 | 0.0343 |
| Per 1-SD | 0.84 (0.76-0.93) | 0.85 (0.76-0.95) | 0.81 (0.65-1.01) | 0.85 (0.76-0.95) | 0.82 (0.68-1.00) | 0.81 (0.73-0.91) | 0.97 (0.77-1.23) | 0.80 (0.70-0.91) | 0.88 (0.76-1.02) |
| P-value | 0.0006 | 0.0043 | 0.0644 | 0.0056 | 0.0461 | 0.0002 | 0.8219 | 0.0008 | 0.0867 |
| P_difference_ | NA | 0.50 | | 0.60 | | 0.20 | | 0.24 | |
| **Females** |  |  |  |  |  |  |  |  |  |
| No cases |  |  |  |  |  |  |  |  |  |
| -1.45 to -0.692 | Reference | Reference | Reference | Reference | Reference | Reference | Reference | Reference | Reference |
| -0.691 to 0.212 | 1.04 (0.83-1.29) | 1.01 (0.78-1.30) | 1.09 (0.75-1.58) | 0.96 (0.74-1.26) | 1.24 (0.90-1.72) | 1.05 (0.81-1.35) | 1.13 (0.78-1.64) | 1.07 (0.82-1.40) | 0.97 (0.69-1.37) |
| 0.211 to 3.025 | 0.96 (0.77-1.20) | 0.94 (0.73-1.22) | 0.88 (0.59-1.31) | 1.02 (0.79-1.32) | 0.91 (0.64-1.29) | 1.02 (0.79-1.32) | 0.74 (0.46-1.11) | 0.99 (0.75-1.30) | 0.87 (0.61-1.23) |
| P_trend_ | 0.7532 | 0.6746 | 0.5736 | 0.8826 | 0.6898 | 0.8691 | 0.1957 | 0.9815 | 0.4361 |
| Per 1-SD | 0.95 (0.87-1.05) | 0.96 (0.76-1.07) | 0.91 (0.77-1.08) | 0.97 (0.87-1.09) | 0.94 (0.82-1.09) | 0.97 (0.87-1.09) | 0.87 (0.73-1.04) | 0.98 (0.87-1.10) | 0.91 (0.78-1.06) |
| P-value | 0.3396 | 0.4175 | 0.2899 | 0.6491 | 0.4416 | 0.6322 | 0.1317 | 0.7037 | 0.2417 |
| P_difference_ | NA | 0.63 | | 0..79 | | 0.29 | | 0.45 | |
| **Case-controls studies** | | | | | | | | | |
| **Sex-combined** |  |  |  |  |  |  |  |  |  |
| No cases | 2,321 | 1,843 | 346 | 1,688 | 379 | 1,813 | 217 | 1,452 | 677 |
| -1.58 to -0.569 | Reference | Reference | Reference | Reference | Reference | Reference | Reference | Reference | Reference |
| -0.568 to 0.182 | 0.83 (0.73-0.95) | 0.84 (0.73-0.97) | 0.76 (0.58-0.99) | 0.85 (0.74-0.98) | 0.80 (0.62-1.03) | 0.84 (0.73-0.97) | 0.85 (0.61-1.18) | 0.80 (0.69-0.93) | 0.89 (0.73-1.08) |
| 0.181 to 2.73 | 0.97 (0.85-1.11) | 0.99 (0.86-1.14) | 0.92 (0.70-1.20) | 1.01 (0.87-1.16) | 0.90 (0.69-1.17) | 0.97 (0.85-1.12) | 0.95 (0.68-1.34) | 0.90 (0.77-1.05) | 0.94 (0.77-1.15) |
| P_trend_ | 0.6373 | 0.8310 | 0.4735 | 0.9534 | 0.3936 | 0.6780 | 0.7545 | 0.1509 | 0.5340 |
| Per 1-SD | 1.00 (0.94-1.05) | 1.01 (0.95-1.07) | 0.96 (0.85-1.08) | 1.01 (0.95-1.07) | 0.94 (0.84-1.06) | 1.00(0.95-1.06) | 0.94 (0.80-1.09) | 0.97 (0.91-1.03) | 0.98 (0.90-1.07) |
| P-value | 0.816 | 0.8494 | 0.4836 | 0.7096 | 0.3181 | 0.9304 | 0.3898 | 0.3274 | 0.6730 |
| P_difference_ | NA | 0.37 | | 0.20 | | 0.32 | | 0.65 | |
| **Males** |  |  |  |  |  |  |  |  |  |
| No cases | 1,278 | 1,037 | 151 | 977 | 175 | 1,031 | 90 | 782 | 397 |
| -1.58 to -0.569 | Reference | Reference | Reference | Reference | Reference | Reference | Reference | Reference | Reference |
| -0.568 to 0.182 | 0.82 (0.69-0.97) | 0.79 (0.65-0.95) | 0.86 (0.58-1.28) | 0.82 (0.68-0.99) | 0.75 (0.52-1.09) | 0.79 (0.65-0.95) | 1.02 (0.63-1.65) | 0.80 (0.65-0.98) | 0.82 (0.63-1.07) |
| 0.181 to 2.73 | 0.87 (0.73-1.03) | 0.91 (0.76-1.09) | 1.01 (0.99-1.10) | 0.93 (0.78-1.12) | 0.68 (0.46-0.99) | 0.91 (0.76-1.09) | 0.56 (0.31-0.99) | 0.82 (0.67-1.01) | 0.85 (0.66-1.11) |
| P_trend_ | 0.1104 | 0.3010 | 0.1351 | 0.4871 | 0.0447 | 0.3191 | 0.0612 | 0.00576 | 0.2393 |
| Per 1-SD | 0.96 (0.90-1.03) | 0.99 (092-1.06) | 0.89 (0.74-1.06) | 1.00 (0.93-1.08) | 0.83 (0.70-0.98) | 0.99 (0.92-1.06) | 0.78 (0.61-0.99) | 0.95 (0.88-1.03) | 0.95 (0.85-1.05) |
| P-value | 0.2780 | 0.7167 | 0.1776 | 0.9975 | 0.0323 | 0.7550 | 0.0436 | 0.2286 | 0.3199 |
| P_difference_ | NA | 0.22 | | 0.02 | | 0.03 | | 0.97 | |
| **Females** |  |  |  |  |  |  |  |  |  |
| No cases | 1,043 | 806 | 195 | 711 | 204 | 782 | 127 | 670 | 280 |
| -1.58 to -0.569 | Reference | Reference | Reference | Reference | Reference | Reference | Reference | Reference | Reference |
| -0.568 to 0.182 | 0.86 (0.71-1.04) | 0.92 (0.75-1.14) | 0.68 (0.47-0.99) | 0..91 (0.73-1.13) | 0.85 (0.59-1.21) | 0.92 (0.75-1.13) | 0.71 (0.44-1.12) | 0.80 (0.64-1.00) | 0.98 (0.72-1.34) |
| 0.181 to 2.73 | 1.14 (0.93-1.39) | 1.11 (0.90-1.38) | 1.14 (0.79-1.14) | 1.12 (0.89-1.40) | 1.18 (0.82-1.70) | 1.07 (0.86-1.33) | 1.32 (0.85-2.04) | 1.03 (0.82-1.30) | 1.08 (0.78-1.49) |
| P_trend_ | 0.2505 | 0.3713 | 0.6144 | 0.36 | 0.4228 | 0.5836 | 0.2627 | 0.9322 | 0.6537 |
| Per 1-SD | 1.05 (0.986-1.15) | 1.04 (0.95-1.14) | 1.04 (0.88-1.23) | 1.03 (0.93-1.14) | 1.07 (0.91-1.26) | 1.03 (0.93-1.13) | 1.07 (0.88-1.31) | 1.00 (0.90-1.11) | 1.05 (0.91-1.20) |
| P-value | 0.2493 | 0.4213 | 0.6661 | 0.5414 | 0.3910 | 0.5816 | 0.5012 | 0.99890 | 0.5282 |
| P_difference_ | NA | 0.91 | | 0.47 | | 0.51 | | 0.53 | |

^a^ Controls are used as the reference for all odds ratios. CI, confidence interval; CIMP, CpG island methylator phenotype; CRC, colorectal cancer; MSI, microsatellite instability; MSS, microsatellite stable; OR, odds ratio.

^b^ Odds ratios are adjusted for study, age, sex (when not stratified), smoking status, alcohol consumption, education, and red meat intake.

^c^ Case-only analyses used to calculate *P*_difference_.

**Table S13**. Association between physical activity, rectal cancer, and its molecular subtypes^a, b, c^.

|  |  | **Microsatellite instability** | | **CpG island methylator phenotype** | | ***BRAF*** | | ***KRAS*** | |
| --- | --- | --- | --- | --- | --- | --- | --- | --- | --- |
| **MET hr/wk.** | **Rectal cancer**  **OR (95%CI)** | **MSS/MSI-L**  **RRR (95%CI)** | **MSI-H**  **RRR (95%CI)** | **CIMP-low/negative**  **RRR (95%CI)** | **CIMP-high**  **RRR (95%CI)** | ***BRAF*-wild type**  **RRR (95%CI)** | ***BRAF*-mutated**  **RRR (95%CI)** | ***KRAS*-wild type**  **RRR (95%CI)** | ***KRAS*-mutated**  **RRR (95%CI)** |
| **Sex-combined** | |  |  |  |  |  |  |  |  |
| No cases | 1,122 | 1,000 | 13 | 998 | 77 | 1,012 | 20 | 669 | 339 |
| -1.58 to -0.591 | Reference | Reference | Reference | Reference | Reference | Reference | Reference | Reference | Reference |
| -0.591 to 0.2 | 0.84 (0.71-0.98( | 0.83 (0.70-0.97) | 0.43 (0.11-1.69) | 0.84 (0.74-0.99) | 0.80 (0.47-1.34) | 0.86 (0.73-1.01) | 0.41 (0.13-1.33) | 0.80 (0.66-0.97) | 0.91 (0.70-1.18) |
| 0.2 to 3.03 | 0.76 *0.65-0.90) | 0.77 (0.65-0.91) | 0.41 (0.11-1.64) | 0.77 (0.65-0.91) | 0.55 (0.30-0.99) | 0.77 (0.65-0.92) | 0.61 (0.21-173) | 0.76 (0.62-0.93) | 0.78 (0.59-1.02) |
| P_trend_ | 0.0011 | 0.002 | 0.1770 | 0.0023 | 0.0451 | 0.0032 | 0.3057 | 0.0066 | 0.0750 |
| Per 1-SD | 0.88 (0.82-0.94) | 0.88 (0.82-0.95) | 0.64 (0.33-1.26) | 0.88 (0.81-0.94) | 0.75 (0.57-0.98) | 0.89 (0.82-0.95) | 0.69 (0.40-1.19) | 0.88 (0.80-0.96) | 0.87 (0.77-0.98) |
| P-value | 0.0003 | 0.0005 | 0.1953 | 0.0005 | 0.0342 | 0.0011 | 0.1844 | 0.003 | 0.0188 |
| P_difference_ | NA | 0.42 | | 0.24 | | 0.61 | | 0.88 | |
| **Males** |  |  |  |  |  |  |  |  |  |
| No cases | 688 | 613 | 9 | 617 | 43 | 614 | 12 | 434 | 180 |
| -1.58 to -0.591 | Reference | Reference | Reference | Reference | Reference | Reference | Reference | Reference | Reference |
| -0.591 to 0.2 | 0.86 (0.70-1.06) | 0.84 (0.68-1.05) | 0.46 (0.008-2.56) | 0.86 (0.69-1.07) | 0.87 (0.43-1.78) | 0.86 (0.69-1.06) | 0.66 (0.15-3.03) | 0.79 (00.61-1.01) | 1.11 (0.76-1.61) |
| 0.2 to 3.03 | 0.76 (0.61-0.94) | 0.76 (0.61-0.95) | 0.65 (0.14-2.99) | 0.76 (0.61-0.95) | 0.60 (0.27-1.31) | 0.75 (0.60-0.94) | 1.02 (0.26-3.99) | 0.71 (0.55-0.91) | 1.00 (0.68-1.46) |
| P_trend_ | 0.0107 | 0.0140 | 0.5669 | 0.0142 | 0.2014 | 0.0109 | 0.9752 | 0.0075 | 0.9670 |
| Per 1-SD | 0.89 (0.81-0.97) | 0.89 (0.81-0.97) | 0.77 (0.37-1.58) | 0.88 (0.81-0.97) | 0.75 (0.53-1.06) | 0.88 (0.81-0.97) | 0.86 (0.47-1.59) | 0.86 (0.77-0.95) | 0.94 (0.81-1.00) |
| P-value | 0.0064 | 0.01 | 0.4693 | 0.0087 | 0.1003 | 0.008 | 0.3676 | 0.0051 | 0.4522 |
| P_difference_ | NA | 0.92 | | 0.25 | | 0.42 | | 0.42 | |
| **Females** |  |  |  |  |  |  |  |  |  |
| No cases | 434 | 387 | 4 | 381 | 34 | 398 | 8 | 235 | 159 |
| -1.58 to -0.591 | Reference | Reference | Reference | Reference | Reference | Reference | Reference | Reference | Reference |
| -0.591 to 0.2 | 0.80 (0.63-1.02) | 0.81 (0.62-1.05) | 0.53 (0.05-5.66) | 0.82 (0.63-1.06) | 0.78 (0.36-1.79) | 0.87 (0.67-1.12) | 0.22 (0.03-1.84) | 0.86 (0.62-1.19) | 0.74 (0.51-1.08) |
| 0.2 to 3.03 | 0.78 (0.60-1.01) | 0.80 (0.61-1.05) | NA | 0.79 (0.60-1.04) | 0.53 (0.21-1.32) | 0.82 (0.63-1.08) | 0.23 (0.03-1.96) | 0.89 (0.63-1.25) | 0.61 (0.40-0.92) |
| P_trend_ | 0.052 | 0.0918 | 0.0821 | 0.0780 | 0.1720 | 0.1530 | 0.1065 | 0.4607 | 0.0161 |
| Per 1-SD | 0..87 (0.77-0.98) | 0.87 (0.77-0.99) | 0.18 (0.03-1.18) | 0.87 (0.76-0.98) | 0.79 (0.52-1.20) | 0.90 (0.80-1.01) | 0.35 (0.09-1.32) | 0.93 (0.80-1.08) | 0.77 (0.64-0.94) |
| P-value | 0.0253 | 0.0340 | 0.0738 | 0.0268 | 0.2680 | 0.0847 | 0.1203 | 0.3503 | 0.011 |
| P_difference_ | NA | 0.40 | | 0.63 | | 0.16 | | 0.16 | |

^a^ Controls are used as the reference for all odds ratios and relative risk ratio. CI, confidence interval; CIMP, CpG island methylator phenotype; CRC, colorectal cancer; MSI, microsatellite instability; MSS, microsatellite stable; OR, odds ratio; RRR, relative risk ratio.

^b^ Odds and relative risk ratios were adjusted for study, age, sex (when not stratified), smoking status, alcohol consumption, education, and red meat intake.

^c^ Case-only analyses used to calculate *P*_difference_.

**Table S14**. Association between physical activity, rectal cancer, and its molecular subtypes stratified by study design ^a, b, c^.

|  |  | **Microsatellite instability** | | **CpG island methylator phenotype** | | ***BRAF*** | | ***KRAS*** | |
| --- | --- | --- | --- | --- | --- | --- | --- | --- | --- |
| **MET hr/wk.** | **Rectal cancer**  **OR (95%CI)** | **MSS/MSI-L**  **RRR (95%CI)** | **MSI-H**  **RRR (95%CI)** | **CIMP-low/negative**  **RRR (95%CI)** | **CIMP-high**  **RRR (95%CI)** | ***BRAF*-wild type**  **RRR (95%CI)** | ***BRAF*-mutated**  **RRR (95%CI)** | ***KRAS*-wild type**  **RRR (95%CI)** | ***KRAS*-mutated**  **RRR (95%CI)** |
| **Cohort studies** | | | | | | | | | |
| **Sex-combined** |  |  |  |  |  |  |  |  |  |
| No cases | 393 | 345 | 5 | 336 | 19 | 362 | 10 | 214 | 127 |
| -1.45 to -0.692 | Reference | Reference | Reference | Reference | Reference | Reference | Reference | Reference | Reference |
| -0.691 to 0.212 | 0.81 (0.62-1.05) | 0.81 (0.61-1.07) | 0.35 (0.03-3.66) | 0.80 (0.60-1.06) | 0.46 (0.13-1.61) | 0.89 (0.68-1.17) | 0.09 (0.01-0.77) | 0.72 (0.50-1.02) | 0.98 (0.63-1.50) |
| 0.211 to 3.025 | 0.80 (0.60-1.06) | 0.85 (0.64-1.15) | 0.33 (0.03-4.10) | 0.76 (0.56-1.03) | 0.97 (0.32-2.94) | 0.90 (0.67-1.20) | 0.21 (0.04-1.16) | 0.94 (0.66-1.35) | 0.79 (0.49-1.27) |
| P_trend_ | 0.1185 | 0.2886 | 0.3379 | 0.0734 | 0.9770 | 0.4663 | 0.0388 | 0.7363 | 0.3447 |
| Per 1-SD | 0.90 (0.80-1.02) | 0.91 (0.81-1.04) | 0.53 (0.15-1.85) | 0.88 (0.77-1.00) | 0.95 (0.58-1.56) | 0.94 (0.83-1.06) | 0.44 (0.16-1.22) | 0.97 (0.83-1.13) | 0.90 (0.74-1.10) |
| P-value | 0.0950 | 0.1653 | 0.3205 | 0.0505 | 0.8428 | 0.3013 | 0.1152 | 0.6864 | 0.3017 |
| P_difference_ | NA | 0.72 | | 0.64 | | 0.25 | | 0.33 | |
| **Males** |  |  |  |  |  |  |  |  |  |
| No cases | 216 | 187 | 2 | 182 | 10 | 196 | 5 | 130 | 51 |
| -1.45 to -0.692 | Reference | Reference | Reference | Reference | Reference | Reference | Reference | Reference | Reference |
| -0.691 to 0.212 | 0.63 (0.43-0.91) | 0.61 (0.41-0.91) | NE | 0.58 (0.39-0.86) | 0.87 (0.14-5.50) | 0.67 (0.46-0.99) | 0.21 (0.02-2.90) | 0.49 (0.30-0.78) | 1.32 (0.63-2.75) |
| 0.211 to 3.025 | 0.60 (0.41-0.89) | 0.64 (0.42-0.96) | NE | 0.56 (0.37-0.84) | 1.15 (0.20-6.80) | 0.64 (0.43-0.96) | 0.52 (0.05-5.00) | 0.66 (0.41-1.04) | 0.93 (0.41-2.07) |
| P_trend_ | 0.0117 | 0.0322 | NE | 0.0059 | 0.8294 | 0.0335 | 0.6246 | 0.0727 | 0.7723 |
| Per 1-SD | 0.80 (0.67-0.95) | 0.81 (0.68-0.97) | 1.83 (0.39-8.71) | 0.78 (0.65-0.94) | 0.71 (0.34-1.49) | 0.82 (0.68-0.97) | 0.79 (0.27-2.30) | 0.83 (0.68-1.03) | 0.89 (0.66-1.21) |
| P-value | 0.0102 | 0.0246 | 0.4453 | 0.0088 | 0.3673 | 0.0224 | 0.6719 | 0.0910 | 0.4655 |
| P_difference_ | NA | 0.99 | | 0.99 | | 0.19 | | 0.64 | |
| **Females** |  |  |  |  |  |  |  |  |  |
| No cases | 177 | 158 | 3 | 154 | 9 | 166 | 5 | 84 | 76 |
| -1.45 to -0.692 | Reference | Reference | Reference | Reference | Reference | Reference | Reference | Reference | Reference |
| -0.691 to 0.212 | 1.04 (0.70-1.53) | 1.07 (0.71-1.62) | 0.18 (0.01-6.03) | 1.11 (0.74-1.68) | 0.18 (0.02-1.71) | 1.20 (0.80-1.80) | NE | 1.26 (0.71-2.21) | 0.84 (0.49-1.47) |
| 0.211 to 3.025 | 1.07 (0.71-1.62) | 1.17 (0.75-1.80) | NE | 1.05 (0.67-1.63) | 1.01 (0.21-4.99) | 1.29 (0.84-1.98) | NE | 1.58 (0.89-2.80) | 0.75 (0.41-1.40) |
| P_trend_ | 0.7336 | 0.4897 | 0.1054 | 0.8139 | 0.8135 | 0.2432 | NE | 0.1206 | 0.3566 |
| Per 1-SD | 1.02 (0.86-1.20) | 1.03 (0.86-1.23) | NE | 0.98 (0.82-1.18) | 1.26 (0.65-2.47) | 1.08 (0.91-1.28) | NE | 1.17 (0.93-1.46) | 0.89 (0.68-1.17) |
| P-value | 0.8335 | 0.7602 | NE | 0.8708 | 0.4949 | 0.3803 | NE | 0.1801 | 0.4026 |
| P_difference_ | NA | 0.95 | | 0.71 | | 0.0 | | 0.07 | |
| **Case-controls studies** | | | | | | | | | |
| **Sex-combined** |  |  |  |  |  |  |  |  |  |
| No cases | 713 | 641 | 8 | 649 | 57 | 638 | 9 | 446 | 206 |
| -1.58 to -0.569 | Reference | Reference | Reference | Reference | Reference | Reference | Reference | Reference | Reference |
| -0.568 to 0.182 | 0.88 (0.71-1.07) | 0.83 (0.67-1.03) | 0.96 (0.19-4.88) | 0.86 (0.70-1.07) | 0.98 (0.54-1.79) | 0.88 (0.71-1.09) | 0.67 (0.11-4.08) | 0.79 (0.62-1.01) | 1.03 (0.73-1.46) |
| 0.181 to 2.73 | 0.81 (0.66-1.00) | 0.78 (0.63-0.97) | 0.62 (0.10-3.85) | 0.82 (0.66-1.02) | 0.55 (0.27-1.12) | 0.79 (0.63-0.98) | 1.29 (0.27-6.12) | 0.73 (0.56-0.94) | 0.90 (0.63-1.29) |
| P_trend_ | 0.0486 | 0.0258 | 0.6143 | 0.0706 | 0.1143 | 0.0349 | 0.7195 | 0.0114 | 0.5778 |
| Per 1-SD | 0.91 (0.83-0.99) | 0.91 (0.83-0.99) | 0.72 (0.32-1.61) | 0.92 (0.84-1.01) | 0.75 (0.54-1.02) | 0.90 (0.82-0.99) | 0.93 (0.46-1.87) | 0.88 (0.79-0.98) | 0.90 (0.77-1.05) |
| P-value | 0.0349 | 0.0376 | 0.4271 | 0.0701 | 0.0682 | 0.0349 | 0.8293 | 0.0207 | 0.1742 |
| P_difference_ | NA | 0.35 | | 0.65 | | 0.62 | | 0.47 | |
| **Males** |  |  |  |  |  |  |  |  |  |
| No cases | 465 | 420 | 7 | 430 | 32 | 414 | 6 | 301 | 126 |
| -1.58 to -0.569 | Reference | Reference | Reference | Reference | Reference | Reference | Reference | Reference | Reference |
| -0.568 to 0.182 | 0.94 (0.72-1.22) | 0.90 (0.69-1.18) | 0.62 (0.10-3.87) | 0.93 (0.71-1.22) | 0.96 (0.42-2.20) | 0.92 (0.70-1.20) | 0.44 (0.04-4.98) | 0.86 (0.63-1.17) | 1.09 (0.69-1.74) |
| 0.181 to 2.73 | 0.89 (0.68-1.15) | 0.86 (0.66-1.13) | 0.54 (0.09-3.37) | 0.90 (0.69-1.18) | 0.57 (0.22-1.46) | 0.87 (0.66-1.14) | 1.16 (0.18-7.62) | 0.76 (0.56-1.04) | 1.18 (0.75-1.85) |
| P_trend_ | 0.3702 | 0.2784 | 0.5069 | 0.4528 | 0.2531 | 0.3046 | 0.8216 | 0.0848 | 0.4837 |
| Per 1-SD | 0.95 (0.85-1.05) | 0.95 (0.85-1.06) | 0.72 (0.31-1.65) | 0.96 (0.86-1.07) | 0.81 (0.55-1.19) | 0.95 (0.85-1.06) | 0.87 (0.38-2.01) | 0.90 (0.79-1.02) | 1.00 (0.83-1.09) |
| P-value | 0.3426 | 0.3514 | 0.4353 | 0.4292 | 0.2834 | 0.3353 | 0.7498 | 0.1025 | 0.9734 |
| P_difference_ | NA | 0.46 | | 0.13 | | 0.12 | | 0.14 | |
| **Females** |  |  |  |  |  |  |  |  |  |
| No cases | 248 | 221 | 1 | 219 | 25 | 224 | 3 | 145 | 80 |
| -1.58 to -0.569 | Reference | Reference | Reference | Reference | Reference | Reference | Reference | Reference | Reference |
| -0.568 to 0.182 | 0.81 (0.58-1.13) | 0.76 (0.54-1.07) | NE | 0.77 (0.55-1.10) | 1.16 (0.47-2.878) | 0.86 (0.61-1.21) | 1.28 (0.08-21.71) | 0.74 (0.49-1.12) | 0.94 (0.56-1.58) |
| 0.181 to 2.73 | 0.71 (0.49-1.02) | 0.68 (0.47-0.99) | NE | 0.70 (0.48-1.02) | 0.61 (0.20-1.86) | 0.69 (0.47-1.00) | 1.49 (0.09-24.94) | 0.71 (0.45-1.11) | 0.57 (0.30-1.06) |
| P_trend_ | 0.0574 | 0.0370 | NE | 0.0569 | 0.4272 | 0.0537 | 0.7814 | 0.1105 | 0.0897 |
| Per 1-SD | 0.82 (0.69-0.97) | 0.82 (0.68-0.98) | NE | 0.83 (0.70-1.00) | 0.71 (0.42-1.20) | 0.82 (0.69-0.98) | 1.02 (0.26-3.92) | 0.85 (0.69-1.05) | 0.72 (0.53-0.97) |
| P-value | 0.0255 | 0.0274 | NE | 0.0447 | 0.1956 | 0.0291 | 0.9822 | 0.1299 | 0.0285 |
| P_difference_ | NA | NE | | 0.36 | | 0.54 | | 0.32 | |

^a^ Controls are used as the reference for all odds ratios. CI, confidence interval; CIMP, CpG island methylator phenotype; CRC, colorectal cancer; MSI, microsatellite instability; MSS, microsatellite stable; OR, odds ratio.

^b^ Odds ratios are adjusted for study, age, sex (when not stratified), smoking status, alcohol consumption, education, and red meat intake.

^c^ Case-only analyses used to calculate *P*_difference_.

**Table S15**. Association between physical activity, colorectal cancer, and its molecular subtypes^a, b, c^ stratifying by early and later-onset.

|  |  | **Microsatellite instability** | | **CpG island methylator phenotype** | | ***BRAF*** | | ***KRAS*** | |
| --- | --- | --- | --- | --- | --- | --- | --- | --- | --- |
| **MET hr/wk.** | **Overall CRC**  **OR (95%CI)** | **MSS/MSI-L**  **RRR (95%CI)** | **MSI-H**  **RRR (95%CI)** | **CIMP-low/negative**  **RRR (95%CI)** | **CIMP-high**  **RRR (95%CI)** | ***BRAF*-wild type**  **RRR (95%CI)** | ***BRAF*-mutated**  **RRR (95%CI)** | ***KRAS*-wild type**  **RRR (95%CI)** | ***KRAS*-mutated**  **RRR (95%CI)** |
| **Early-onset (Age ≤ 50 years old)** | | | | | | | | | |
| **Sex-combined** |  |  |  |  |  |  |  |  |  |
| No cases | 224 | 180 | 27 | 182 | 17 | 192 | 8 | 142 | 67 |
| -1.58 to -0.499 | Reference | Reference | Reference | Reference | Reference | Reference | Reference | Reference | Reference |
| -0.500 to 0.139 | 0.61 (0.68-0.98) | 0.54 (0.32-0.90) | 1.21 (0.45-3.29) | 0.70 (0.43-1.16) | 0.62 (0.16-2.45) | 0.68 (0.42-1.11) | 0.31 (0.03-3.88) | 0.61 (0.35-1.05) | 0.73 (0.37-1.47) |
| 0.140 to 2.73 | 0.97 (0.62-1.51) | 0.96 (0.60-1.52) | 0.67 (0.22-2.04) | 1.03 (0.64-1.65) | 1.16 (0.35-3.86) | 1.01 (0.64-1.61) | 1.54 (0.26-9.24) | 0.94 (0.56-1.56) | 0.91 (0.47-1.77) |
| P_trend_ | 0.9402 | 0.8795 | 0.5080 | 0.8602 | 0.7856 | 0.9265 | 0.4591 | 0.8207 | 0.8068 |
| Per 1-SD | 1.05 (0.87-1.27) | 1.08 (0.88-1.32) | 0.78 (0.49-1.22) | 1.10 (0.90-1.35) | 0.84 (0.44-1.58) | 1.07 (0.87-1.30) | 2.78 (1.23-6.02) | 1.08 (0.87-1.35) | 0.90 (0.67-1.22) |
| P-value | 0.6338 | 0.4442 | 0.2766 | 0.3343 | 0.5861 | 0.5256 | 0.0135 | 0.4677 | 0.5118 |
| P_difference_ | NA | 0.15 | | 0.30 | | 0.02 | | 0.366 | |
| **Later-onset (Age > 50 years old)** | | | | | | | | | |
| **Sex-combined** |  |  |  |  |  |  |  |  |  |
| No cases | 4,765 | 3,735 | 624 | 3,538 | 832 | 3,755 | 485 | 2,788 | 1,434 |
| -1.58 to -0.591 | Reference | Reference | Reference | Reference | Reference | Reference | Reference | Reference | Reference |
| -0.590 to 0.2 | 0.86 (0.78-0.94) | 0.85 (0.77-0.94) | 0.82 (0.67-1.01) | 0.85 (0.77-0.94) | 0.90 (0.76-1.07) | 0.86 (0.78-0.95) | 0.90 (0.72-1.13) | 0.85 (0.76-0.95) | 0.84 (0.73-0.96) |
| 0.21 to 3.025 | 0.85 (0.77-0.94) | 0.86 (0.77-0.95) | 0.80 (0.65-0.99) | 0.87 (0.79-0.99) | 0.79 (0.65-0.95) | 0.85 (0.77-0.94) | 0.79 (0.62-0.99) | 0.82 (0.73-0.91) | 0.84 (0.73-0.97) |
| P_trend_ | 0.0008 | 0.0027 | 0.0328 | 0.0072 | 0.0113 | 0.0018 | 0.0458 | 0.0004 | 0.0131 |
| Per 1-SD | 0.93 (0.90-0.97) | 0.94 (0.90-0.98) | 0.91 (0.83-0.99) | 0.94 (0.90-0.98) | 0.90 (0.83-0.97) | 0.93 (0.90-0.98) | 0.89 (0.80-0.98) | 0.92 (0.87-0.96) | 0.93 (0.87-0.99) |
| P-value | 0.0007 | 0.0024 | 0.038 | 0.0054 | 0.0091 | 0.0018 | 0.0202 | 0.0003 | 0.0176 |
| P_difference_ | NA | 0.42 | | 0.21 | | 0.25 | | 0.59 | |

^a^ Controls are used as the reference for all odds ratios. CI, confidence interval; CIMP, CpG island methylator phenotype; CRC, colorectal cancer; MSI, microsatellite instability; MSS, microsatellite stable; OR, odds ratio.

^b^ Odds ratios are adjusted for study, age, sex (when not stratified), smoking status, alcohol consumption, education, and red meat intake.

^c^ Case-only analyses used to calculate *P*_difference_.

**Table S16**. Association between physical activity, colorectal cancer, and its molecular subtypes according to the observational analysis after conducting multiple imputation^a, b, c^.

|  |  | **Microsatellite instability** | | **CpG island methylator phenotype** | | **BRAF** | | **KRAS** | |
| --- | --- | --- | --- | --- | --- | --- | --- | --- | --- |
| **MET hr/wk.** | **Overall CRC**  **OR (95%CI)** | **MSS/MSI-L**  **RRR (95%CI)** | **MSI-H**  **RRR (95%CI)** | **CIMP-low/negative**  **RRR (95%CI)** | **CIMP-high**  **RRR (95%CI)** | **BRAF-wild type**  **RRR (95%CI)** | **BRAF-mutated**  **RRR (95%CI)** | **KRAS-wild type**  **RRR (95%CI)** | **KRAS-mutated**  **RRR (95%CI)** |
| **Sex-combined** |  |  |  |  |  |  |  |  |  |
| -1.58 to -0.591 | Reference | Reference | Reference | Reference | Reference | Reference | Reference | Reference | Reference |
| -0.590 to 0.2 | 0.88 (0.81-0.96) | 0.87 (0.79-0.96) | 0.85 (0.70-1.03) | 0.88 (0.80-0.97) | 0.91 (0.77-1.02) | 0.89 (0.81-0.98) | 0.92 (0.74-1.14) | 0.86 (0.77-0.95) | 0.89 (0.78-1.02) |
| 0.21 to 3.03 | 0.90 (0.82-0.99) | 0.90 (0.82-0.99) | 0.85 (0.70-1.04) | 0.92 (0.83-1.01) | 0.83 (0.69-0.99) | 0.90 (0.81-0.99) | 0.87 (0.69-1.09) | 0.86 (0.77-0.96) | 0.89 (0.77-1.02) |
| P_trend_ | 0.021 | 0.0285 | 0.1132 | 0.0775 | 0.0442 | 0.0267 | 0.2214 | 0.0054 | 0.0885 |
| Per 1-SD | 0.96 (0.92-1.00) | 0.96 (0.92-1.00) | 0.94 (0.86-1.02) | 0.96 (0.92-1.01) | 0.92 (0.85-1.00) | 0.96 (0.92-1.00) | 0.93 (0.84-1.03) | 0.94 (0.90-0.99) | 0.95 (0.89-1.00) |
| P-value | 0.028 | 0.0444 | 0.1386 | 0.0878 | 0.0457 | 0.0365 | 0.1713 | 0.0128 | 0.070 |
| P_difference_ | NA | 0.5244 | | 0.2345 | | 0.5937 | | 0.7762 | |
| **Males** |  |  |  |  |  |  |  |  |  |
| -1.58 to -0.591 | Reference | Reference | Reference | Reference | Reference | Reference | Reference | Reference | Reference |
| -0.590 to 0.2 | 0.84 (0.74-0.95) | 0.81 (0.71-0.93) | 0.84 (0.62-1.13) | 0.83 (0.72-0.95) | 0.79 (0.61-1.03) | 0.82 (0.72-0.93) | 0.91 (0.63-1.29) | 0.81 (0.70-0.94) | 0.84 (0.70-1.01) |
| 0.21 to 3.03 | 0.81 (0.71-0.92) | 0.82 (0.72-0.94) | 0.71 (0.52-0.97) | 0.85 (0.74-0.97) | 0.65 (0.49-0.86) | 0.82 (0.72-0.94) | 0.69 (0.47-1.02) | 0.75 (0.65-0.88) | 0.88 (0.73-1.06) |
| P_trend_ | 0.0012 | 0.004 | 0.0328 | 0.0143 | 0.0025 | 0.0031 | 0.0668 | 0.0002 | 0.1828 |
| Per 1-SD | 0.93 (0.88-0.98) | 0.94 (0.89-0.99) | 0.89 (0.78-1.01) | 0.95 (0.90-1.00) | 0.83 (0.74-0.94) | 0.93 (0.89-0.99) | 0.88 (0.75-1.03) | 0.90 (0.85-0.96) | 0.95 (0.88-1.03) |
| P-value | 0.0053 | 0.0174 | 0.0858 | 0.0583 | 0.0027 | 0.0132 | 0.1244 | 0.0012 | 0.2108 |
| P_difference_ | NA | 0.4262 | | 0.024 | | 0.4548 | | 0.2040 | |
| **Females** |  |  |  |  |  |  |  |  |  |
| -1.58 to -0.591 | Reference | Reference | Reference | Reference | Reference | Reference | Reference | Reference | Reference |
| -0.590 to 0.2 | 0.92 (0.80-1.05) | 0.94 (0.81-1.08) | 0.87 (0.68-1.12) | 0.93 (0.80-1.07) | 1.01 (0.81-1.26) | 0.96 (0.84-1.11) | 0.94 (0.72-1.23) | 0.91 (0.78-1.06) | 0.94 (0.78-1.14) |
| 0.21 to 3.03 | 1.04 (0.90-1.19) | 0.99 (0.86-1.14) | 1.00 (0.77-1.29) | 1.01 (0.87-1.17) | 1.00 (0.79-1.26) | 1.00 (0.86-1.15) | 0.99 (0.75-1.32) | 1.00 (0.85-1.17) | 0.89 (0.72-1.09) |
| P_trend_ | 0.6950 | 0.8461 | 0.9033 | 0.9951 | 0.9914 | 0.9162 | 0.9242 | 0.9016 | 0.2562 |
| Per 1-SD | 0.99 (0.94-1.05) | 0.99 (0.93-1.06) | 0.98 (0.88-1.10) | 0.98 (0.92-1.05) | 1.00 (0.90-1.11) | 0.99 (0.93-1.05) | 0.97 (0.85-1.10) | 0.99 (0.93-1.07) | 0.94 (0.86-1.03) |
| P-value | 0.8103 | 0.6721 | 0.7731 | 0.6276 | 0.9937 | 0.7071 | 0.6121 | 0.8558 | 0.1704 |
| P_difference_ | NA | 0.9598 | | 0.6331 | | 0.8899 | | 0.3008 | |

^a^ Controls are used as the reference for all effect sizes. CI, confidence interval; CIMP, CpG island methylator phenotype; CRC, colorectal cancer; MSI, microsatellite instability; MSS, microsatellite stable; OR, odds ratio; RRR, relative risk ratio.

^b^ Odds ratios are adjusted for study, age, sex (when not stratified), smoking status, alcohol consumption, education, and red meat intake.

^c^ Case-only analysis used to calculate *P*_difference_.

**Table S17**. Genetic instrument-physical activity and instrument-colorectal cancer molecular subtypes associations used for the Mendelian randomisation analyses.

| **Exposure** | **Outcome** | **rsid** | **pa_effectallele** | **pa_noneffectallele** | **pa_eaf** | **pa_beta** | **pa_se** | **pval** | **Outcome_effectallele** | **Outcome_noneffectallele** | **Outcome_beta_not_aligned** | **Outcome_se** | **Outcome_pval** | **Outcome_beta** |
| --- | --- | --- | --- | --- | --- | --- | --- | --- | --- | --- | --- | --- | --- | --- |
| Physical activity | **Overall CRC** | rs11012732 | A | G | 0.668 | 0.028 | 0.005 | 4.10E-09 | G | A | 0.0194 | 0.0078 | 1.33E-02 | -0.0194 |
| Physical activity | **Overall CRC** | rs62055696 | A | G | 0.783 | -0.037 | 0.005 | 6.20E-12 | G | A | -0.0518 | 0.0169 | 2.15E-03 | 0.0518 |
| Physical activity | **Overall CRC** | rs1518139 | G | T | 0.662 | -0.028 | 0.005 | 3.00E-09 | T | G | -0.0084 | 0.0078 | 2.79E-01 | 0.0084 |
| Physical activity | **Overall CRC** | rs336606 | T | C | 0.276 | 0.027 | 0.005 | 4.00E-08 | C | T | 0.0145 | 0.0081 | 7.43E-02 | -0.0145 |
| Physical activity | **Overall CRC** | rs6873698 | C | T | 0.663 | 0.027 | 0.005 | 2.60E-08 | T | C | -0.0132 | 0.0149 | 3.75E-01 | 0.0132 |
| Physical activity | **Overall CRC** | rs9293503 | T | C | 0.888 | 0.039 | 0.007 | 4.90E-08 | C | T | 0.0112 |  |  | -0.0112 |
| Physical activity | ***BRAF* mutation** | rs11012732 | A | G | 0.6678 | 0.0277 | 0.0047 | 4.10E-09 | G | A | 0.0901 | 0.0544 | 9.76E-02 | -0.0901 |
| Physical activity | ***BRAF* mutation** | rs56194509 | T | G | 0.7796 | -0.0368 | 0.0054 | 7.40E-12 | G | T | 0.0475 | 0.0625 | 4.48E-01 | -0.0475 |
| Physical activity | ***BRAF* mutation** | rs59499656 | A | T | 0.6555 | -0.0281 | 0.0047 | 1.90E-09 | T | A | -0.0137 | 0.0531 | 7.96E-01 | 0.0137 |
| Physical activity | ***BRAF* mutation** | rs6775319 | A | T | 0.2707 | 0.0274 | 0.0050 | 3.90E-08 | T | A | -0.0069 | 0.0564 | 9.03E-01 | 0.0069 |
| Physical activity | ***BRAF* mutation** | rs12522261 | G | A | 0.6566 | 0.0260 | 0.0047 | 2.80E-08 | A | G | 0.0063 | 0.0538 | 9.06E-01 | -0.0063 |
| Physical activity | ***BRAF* mutation** | rs9293503 | T | C | 0.8884 | 0.0391 | 0.0072 | 4.90E-08 | C | T | -0.0053 | 0.0808 | 9.48E-01 | 0.0053 |
| Physical activity | ***BRAF* wildtype** | rs11012732 | A | G | 0.6678 | 0.0277 | 0.0047 | 4.10E-09 | G | A | 0.0028 | 0.0257 | 9.14E-01 | -0.0028 |
| Physical activity | ***BRAF* wildtype** | rs56194509 | T | G | 0.7796 | -0.0368 | 0.0054 | 7.40E-12 | G | T | -0.0265 | 0.0285 | 3.51E-01 | 0.0265 |
| Physical activity | ***BRAF* wildtype** | rs59499656 | A | T | 0.6555 | -0.0281 | 0.0047 | 1.90E-09 | T | A | -0.0288 | 0.0243 | 2.36E-01 | 0.0288 |
| Physical activity | ***BRAF* wildtype** | rs6775319 | A | T | 0.2707 | 0.0274 | 0.0050 | 3.90E-08 | T | A | 0.0322 | 0.0257 | 2.11E-01 | -0.0322 |
| Physical activity | ***BRAF* wildtype** | rs12522261 | G | A | 0.6566 | 0.0260 | 0.0047 | 2.80E-08 | A | G | 0.0230 | 0.0246 | 3.49E-01 | -0.0230 |
| Physical activity | ***BRAF* wildtype** | rs9293503 | T | C | 0.8884 | 0.0391 | 0.0072 | 4.90E-08 | C | T | 0.0484 | 0.0357 | 1.75E-01 | -0.0484 |
|  |  |  |  |  |  |  |  |  |  |  |  |  |  |  |
| Physical activity | **CIMP-high** | rs11012732 | A | G | 0.6678 | 0.0277 | 0.0047 | 4.10E-09 | G | A | 0.0852 | 0.0522 | 1.03E-01 | -0.0852 |
| Physical activity | **CIMP-high** | rs56194509 | T | G | 0.7796 | -0.0368 | 0.0054 | 7.40E-12 | G | T | 0.0443 | 0.0584 | 4.48E-01 | -0.0443 |
| Physical activity | **CIMP-high** | rs59499656 | A | T | 0.6555 | -0.0281 | 0.0047 | 1.90E-09 | T | A | -0.0056 | 0.0498 | 9.10E-01 | 0.0056 |
| Physical activity | **CIMP-high** | rs6775319 | A | T | 0.2707 | 0.0274 | 0.0050 | 3.90E-08 | T | A | 0.0171 | 0.0529 | 7.47E-01 | -0.0171 |
| Physical activity | **CIMP-high** | rs12522261 | G | A | 0.6566 | 0.0260 | 0.0047 | 2.80E-08 | A | G | 0.0126 | 0.0505 | 8.03E-01 | -0.0126 |
| Physical activity | **CIMP-high** | rs9293503 | T | C | 0.8884 | 0.0391 | 0.0072 | 4.90E-08 | C | T | 0.0323 | 0.0736 | 6.61E-01 | -0.0323 |
| Physical activity | **CIMP-low/negative** | rs11012732 | A | G | 0.6678 | 0.0277 | 0.0047 | 4.10E-09 | G | A | 0.0019 | 0.0274 | 9.46E-01 | -0.0019 |
| Physical activity | **CIMP-low/negative** | rs56194509 | T | G | 0.7796 | -0.0368 | 0.0054 | 7.40E-12 | G | T | -0.0146 | 0.0301 | 6.28E-01 | 0.0146 |
| Physical activity | **CIMP-low/negative** | rs59499656 | A | T | 0.6555 | -0.0281 | 0.0047 | 1.90E-09 | T | A | -0.0184 | 0.0256 | 4.73E-01 | 0.0184 |
| Physical activity | **CIMP-low/negative** | rs6775319 | A | T | 0.2707 | 0.0274 | 0.0050 | 3.90E-08 | T | A | 0.0231 | 0.0271 | 3.94E-01 | -0.0231 |
| Physical activity | **CIMP-low/negative** | rs12522261 | G | A | 0.6566 | 0.0260 | 0.0047 | 2.80E-08 | A | G | 0.0143 | 0.0260 | 5.82E-01 | -0.0143 |
| Physical activity | **CIMP-low/negative** | rs9293503 | T | C | 0.8884 | 0.0391 | 0.0072 | 4.90E-08 | C | T | 0.0168 | 0.0378 | 6.58E-01 | -0.0168 |
|  |  |  |  |  |  |  |  |  |  |  |  |  |  |  |
| Physical activity | ***KRAS* mutation** | rs11012732 | A | G | 0.6678 | 0.0277 | 0.0047 | 4.10E-09 | G | A | -0.0152 | 0.0375 | 6.85E-01 | 0.0152 |
| Physical activity | ***KRAS* mutation** | rs56194509 | T | G | 0.7796 | -0.0368 | 0.0054 | 7.40E-12 | G | T | 0.0139 | 0.0412 | 7.37E-01 | -0.0139 |
| Physical activity | ***KRAS* mutation** | rs59499656 | A | T | 0.6555 | -0.0281 | 0.0047 | 1.90E-09 | T | A | -0.0163 | 0.0354 | 6.46E-01 | 0.0163 |
| Physical activity | ***KRAS* mutation** | rs6775319 | A | T | 0.2707 | 0.0274 | 0.0050 | 3.90E-08 | T | A | 0.0398 | 0.0377 | 2.92E-01 | -0.0398 |
| Physical activity | ***KRAS* mutation** | rs12522261 | G | A | 0.6566 | 0.0260 | 0.0047 | 2.80E-08 | A | G | -0.0024 | 0.0359 | 9.47E-01 | 0.0024 |
| Physical activity | ***KRAS* mutation** | rs9293503 | T | C | 0.8884 | 0.0391 | 0.0072 | 4.90E-08 | C | T | -0.0383 | 0.0533 | 4.72E-01 | 0.0383 |
| Physical activity | ***KRAS* wildtype** | rs11012732 | A | G | 0.6678 | 0.0277 | 0.0047 | 4.10E-09 | G | A | 0.0347 | 0.0286 | 2.25E-01 | -0.0347 |
| Physical activity | ***KRAS* wildtype** | rs56194509 | T | G | 0.7796 | -0.0368 | 0.0054 | 7.40E-12 | G | T | -0.0151 | 0.0317 | 6.35E-01 | 0.0151 |
| Physical activity | ***KRAS* wildtype** | rs59499656 | A | T | 0.6555 | -0.0281 | 0.0047 | 1.90E-09 | T | A | -0.0463 | 0.0271 | 8.74E-02 | 0.0463 |
| Physical activity | ***KRAS* wildtype** | rs6775319 | A | T | 0.2707 | 0.0274 | 0.0050 | 3.90E-08 | T | A | 0.0002 | 0.0285 | 9.95E-01 | -0.0002 |
| Physical activity | ***KRAS* wildtype** | rs12522261 | G | A | 0.6566 | 0.0260 | 0.0047 | 2.80E-08 | A | G | 0.0251 | 0.0273 | 3.57E-01 | -0.0251 |
| Physical activity | ***KRAS* wildtype** | rs9293503 | T | C | 0.8884 | 0.0391 | 0.0072 | 4.90E-08 | C | T | 0.0461 | 0.0399 | 2.47E-01 | -0.0461 |
|  |  |  |  |  |  |  |  |  |  |  |  |  |  |  |
| Physical activity | **MSI-high** | rs11012732 | A | G | 0.6678 | 0.0277 | 0.0047 | 4.10E-09 | G | A | 0.1500 | 0.0480 | 1.79E-03 | -0.1500 |
| Physical activity | **MSI-high** | rs56194509 | T | G | 0.7796 | -0.0368 | 0.0054 | 7.40E-12 | G | T | -0.0064 | 0.0550 | 9.07E-01 | 0.0064 |
| Physical activity | **MSI-high** | rs59499656 | A | T | 0.6555 | -0.0281 | 0.0047 | 1.90E-09 | T | A | 0.0155 | 0.0468 | 7.40E-01 | -0.0155 |
| Physical activity | **MSI-high** | rs6775319 | A | T | 0.2707 | 0.0274 | 0.0050 | 3.90E-08 | T | A | -0.0429 | 0.0493 | 3.85E-01 | 0.0429 |
| Physical activity | **MSI-high** | rs12522261 | G | A | 0.6566 | 0.0260 | 0.0047 | 2.80E-08 | A | G | 0.0166 | 0.0473 | 7.26E-01 | -0.0166 |
| Physical activity | **MSI-high** | rs9293503 | T | C | 0.8884 | 0.0391 | 0.0072 | 4.90E-08 | C | T | 0.0829 | 0.0688 | 2.28E-01 | -0.0829 |
| Physical activity | **MSS/MSI-L** | rs11012732 | A | G | 0.6678 | 0.0277 | 0.0047 | 4.10E-09 | G | A | -0.0016 | 0.0255 | 9.50E-01 | 0.0016 |
| Physical activity | **MSS/MSI-L** | rs56194509 | T | G | 0.7796 | -0.0368 | 0.0054 | 7.40E-12 | G | T | 0.0030 | 0.0281 | 9.16E-01 | -0.0030 |
| Physical activity | **MSS/MSI-L** | rs59499656 | A | T | 0.6555 | -0.0281 | 0.0047 | 1.90E-09 | T | A | -0.0384 | 0.0241 | 1.12E-01 | 0.0384 |
| Physical activity | **MSS/MSI-L** | rs6775319 | A | T | 0.2707 | 0.0274 | 0.0050 | 3.90E-08 | T | A | 0.0462 | 0.0256 | 7.12E-02 | -0.0462 |
| Physical activity | **MSS/MSI-L** | rs12522261 | G | A | 0.6566 | 0.0260 | 0.0047 | 2.80E-08 | A | G | 0.0188 | 0.0244 | 4.42E-01 | -0.0188 |
| Physical activity | **MSS/MSI-L** | rs9293503 | T | C | 0.8884 | 0.0391 | 0.0072 | 4.90E-08 | C | T | 0.0469 | 0.0355 | 1.87E-01 | -0.0469 |

**Table S18**. Results from Mendelian randomisation analysis for physical activity and risk of colorectal cancer defined by molecular markers.

| **Exposure** | **Outcome** | **MR-Method** | **OR** | **95%LCI** | **95%UCI** | **P-value** | **P-difference** |
| --- | --- | --- | --- | --- | --- | --- | --- |
| Physical activity | Overall CRC | IVW | 0.631 | 0.447 | 0.891 | **0.009** | NA |
| Physical activity | Overall CRC | Weighted median | 0.687 | 0.489 | 0.964 | **0.030** |  |
| Physical activity | Overall CRC | Weighted mode | 0.693 | 0.478 | 1.005 | 0.053 |  |
| Physical activity | Overall CRC | MR-Egger | 0.318 | 0.021 | 4.724 | 0.406 |  |
| Physical activity | Overall CRC | (intercept) |  |  |  | 0.615 |  |
| Physical activity | *BRAF* mutation | IVW | 0.730 | 0.154 | 3.455 | 0.6917 |  |
| Physical activity | *BRAF* mutation | Weighted median | 0.981 | 0.142 | 6.771 | 0.9843 |  |
| Physical activity | *BRAF* mutation | Weighted mode | 0.921 | 0.055 | 15.428 | 0.9546 |  |
| Physical activity | *BRAF* mutation | MR-Egger | 114.275 | 0.003 | 4118002.157 | 0.3761 |  |
| Physical activity | *BRAF* mutation | (intercept) |  |  |  | 0.3399 | 0.3040 |
| Physical activity | *BRAF* wildtype | IVW | 0.427 | 0.210 | 0.867 | **0.0186** |  |
| Physical activity | *BRAF* wildtype | Weighted median | 0.394 | 0.170 | 0.915 | **0.0303** |  |
| Physical activity | *BRAF* wildtype | Weighted mode | 0.364 | 0.113 | 1.171 | 0.0901 |  |
| Physical activity | *BRAF* wildtype | MR-Egger | 0.263 | 0.002 | 29.779 | 0.5797 |  |
| Physical activity | *BRAF* wildtype | (intercept) |  |  |  | 0.8389 |  |
|  |  |  |  |  |  |  |  |
| Physical activity | CIMP-high | IVW | 0.583 | 0.136 | 2.496 | 0.4668 |  |
| Physical activity | CIMP-high | Weighted median | 0.599 | 0.098 | 3.673 | 0.5794 |  |
| Physical activity | CIMP-high | Weighted mode | 0.583 | 0.039 | 8.660 | 0.6950 |  |
| Physical activity | CIMP-high | MR-Egger | 29.361 | 0.002 | 490276.962 | 0.4957 |  |
| Physical activity | CIMP-high | (intercept) |  |  |  | 0.4242 | 0.7390 |
| Physical activity | CIMP-low/negative | IVW | 0.614 | 0.290 | 1.301 | 0.2032 |  |
| Physical activity | CIMP-low/negative | Weighted median | 0.625 | 0.261 | 1.494 | 0.2904 |  |
| Physical activity | CIMP-low/negative | Weighted mode | 0.624 | 0.190 | 2.052 | 0.4377 |  |
| Physical activity | CIMP-low/negative | MR-Egger | 0.917 | 0.006 | 136.994 | 0.9729 |  |
| Physical activity | CIMP-low/negative | (intercept) |  |  |  | 0.8740 |  |
|  |  |  |  |  |  |  |  |
| Physical activity | *KRAS* mutation | IVW | 1.013 | 0.358 | 2.860 | 0.9812 |  |
| Physical activity | *KRAS* mutation | Weighted median | 1.281 | 0.366 | 4.476 | 0.6985 |  |
| Physical activity | *KRAS* mutation | Weighted mode | 1.484 | 0.249 | 8.850 | 0.6648 |  |
| Physical activity | *KRAS* mutation | MR-Egger | 20.758 | 0.020 | 21695.788 | 0.3925 |  |
| Physical activity | *KRAS* mutation | (intercept) |  |  |  | 0.3891 | 0.158 |
| Physical activity | *KRAS* wildtype | IVW | 0.408 | 0.185 | 0.899 | **0.0261** |  |
| Physical activity | *KRAS* wildtype | Weighted median | 0.351 | 0.133 | 0.925 | **0.0343** |  |
| Physical activity | *KRAS* wildtype | Weighted mode | 0.304 | 0.076 | 1.215 | 0.0920 |  |
| Physical activity | *KRAS* wildtype | MR-Egger | 0.687 | 0.004 | 133.720 | 0.8889 |  |
| Physical activity | *KRAS* wildtype | (intercept) |  |  |  | 0.8448 |  |
|  |  |  |  |  |  |  |  |
| Physical activity | MSI-high | IVW | 0.368 | 0.053 | 2.553 | 0.3120 |  |
| Physical activity | MSI-high | Weighted median | 0.704 | 0.115 | 4.295 | 0.7035 |  |
| Physical activity | MSI-high | Weighted mode | 0.986 | 0.093 | 10.512 | 0.9909 |  |
| Physical activity | MSI-high | MR-Egger | 0.220 | 0.000 | 432562.153 | 0.8376 |  |
| Physical activity | MSI-high | (intercept) |  |  |  | 0.9436 | 0.897 |
| Physical activity | MSS/MSI-L | IVW | 0.470 | 0.232 | 0.950 | **0.0355** |  |
| Physical activity | MSS/MSI-L | Weighted median | 0.411 | 0.169 | 1.003 | 0.0508 |  |
| Physical activity | MSS/MSI-L | Weighted mode | 0.283 | 0.067 | 1.196 | 0.0860 |  |
| Physical activity | MSS/MSI-L | MR-Egger | 1.568 | 0.014 | 171.399 | 0.8509 |  |
| Physical activity | MSS/MSI-L | (intercept) |  |  |  | 0.6106 |  |

**Supplementary references**

1. Calle EE, Rodriguez C, Jacobs EJ, Almon ML, Chao A, McCullough ML, et al. The American Cancer Society Cancer Prevention Study II Nutrition Cohort: Rationale, study design, and baseline characteristics. Cancer. 2002 Jan 15;94(2):500–11.

2. Campbell PT, Deka A, Briggs P, Cicek M, Farris AB, Gaudet MM, et al. Establishment of the Cancer Prevention Study II Nutrition Cohort Colorectal Tissue Repository. Cancer Epidemiol Biomarkers Prev. 2014 Dec 1;23(12):2694–702.

3. Belanger CF, Hennekens CH, Rosner B, Speizer FE. The Nurses’ Health Study. Am J Nurs. 1978 Jun;78(6):1039.

4. Giles GG, English DR. The Melbourne Collaborative Cohort Study. IARC Sci Publ 2002;156:69–70.

5. Brenner H, Chang–Claude J, Jansen L, Knebel P, Stock C, Hoffmeister M. Reduced Risk of Colorectal Cancer Up to 10 Years After Screening, Surveillance, or Diagnostic Colonoscopy. Gastroenterology. 2014 Mar;146(3):709–17.

6. Jia M, Jansen L, Walter V, Tagscherer K, Roth W, Herpel E, et al. No association of CpG island methylator phenotype and colorectal cancer survival: population-based study. Br J Cancer. 2016 Nov;115(11):1359–66.

7. Slattery ML, Friedman GD, Potter JD, Edwards S, Caan BJ, Samowitz W. A description of age, sex, and site distributions of colon carcinoma in three geographic areas. Cancer. 1996 Oct 15;78(8):1666–70.

8. Slattery ML, Berry TD, Potter J, Cann B. Diet diversity, diet composition, and risk of colon cancer (United States). Cancer Causes and Control. 1997;8:872–82.

9. Amin W, Singh H, Dzubinski LA, Schoen RE, Parwani AV. Design and utilization of the colorectal and pancreatic neoplasm virtual biorepository: An early detection research network initiative. J Pathol Inform. 2010 Jan;1(1):22.

10. Riboli E, Hunt K, Slimani N, Ferrari P, Norat T, Fahey M, et al. European Prospective Investigation into Cancer and Nutrition (EPIC): study populations and data collection. Public Health Nutr. 2002 Dec;5(6b):1113–24.

11. Dahlin AM, Palmqvist R, Henriksson ML, Jacobsson M, Eklöf V, Rutegård J, et al. The Role of the CpG Island Methylator Phenotype in Colorectal Cancer Prognosis Depends on Microsatellite Instability Screening Status. Clin Cancer Res. 2010 Mar 15;16(6):1845–55.

12. Newcomb PA, Baron J, Cotterchio M, Gallinger S, Grove J, Haile R, et al. Colon Cancer Family Registry: An International Resource for Studies of the Genetic Epidemiology of Colon Cancer. Cancer Epidemiol Biomarkers Prev. 2007 Nov 1;16(11):2331–43.

13. Ogino S, Brahmandam M, Cantor M, Namgyal C, Kawasaki T, Kirkner G, et al. Distinct molecular features of colorectal carcinoma with signet ring cell component and colorectal carcinoma with mucinous component. Mod Pathol. 2006 Jan;19(1):59–68.

14. Boland CR, Thibodeau SN, Hamilton SR, Sidransky D, Eshleman JR, Burt RW, et al. A National Cancer Institute Workshop on Microsatellite Instability for Cancer Detection and Familial Predisposition: Development of International Criteria for the Determination of Microsatellite Instability in Colorectal Cancer.

15. Samowitz WS, Slatteryt ML. Transforming Growth Factor-13 Receptor Type 2 Mutations and Microsatellite Instability in Sporadic Colorectal Adenomas and Carcinomas.

16. Samowitz WS, Slattery ML, Kerber RA. Microsatellite instability in human colonic cancer is not a useful clinical indicator of familial colorectal cancer. Gastroenterology. 1995 Dec;109(6):1765–71.

17. Samowitz WS, Slattery ML, Potter JD, Leppert MF. BAT-26 and BAT-40 Instability in Colorectal Adenomas and Carcinomas and Germline Polymorphisms. Am J Pathol. 1999 Jun;154(6):1637–41.

18. Giovannucci E. Physical Activity, Obesity, and Risk for Colon Cancer and Adenoma in Men. Ann Intern Med. 1995 Mar 1;122(5):327.

19. Hoffmeister M, Bläker H, Kloor M, Roth W, Toth C, Herpel E, et al. Body Mass Index and Microsatellite Instability in Colorectal Cancer: A Population-based Study. Cancer Epidemiol Biomarkers Prev. 2013 Dec 1;22(12):2303–11.

20. Findeisen P, Kloor M, Merx S, Sutter C, Woerner SM, Dostmann N, et al. T25 Repeat in the 3′ Untranslated Region of the *CASP2* Gene: A Sensitive and Specific Marker for Microsatellite Instability in Colorectal Cancer. Cancer Res. 2005 Sep 15;65(18):8072–8.

21. Ogino S, Kawasaki T, Brahmandam M, Yan L, Cantor M, Namgyal C, et al. Sensitive Sequencing Method for KRAS Mutation Detection by Pyrosequencing. J Mol Diagn. 2005 Aug;7(3):413–21.

22. Ogino S, Kawasaki T, Kirkner GJ, Loda M, Fuchs CS. CpG Island Methylator Phenotype-Low (CIMP-Low) in Colorectal Cancer: Possible Associations with Male Sex and KRAS Mutations. J Mol Diagn. 2006 Nov;8(5):582–8.

23. Ogino S, Meyerhardt JA, Cantor M, Brahmandam M, Clark JW, Namgyal C, et al. Molecular Alterations in Tumors and Response to Combination Chemotherapy with Gefitinib for Advanced Colorectal Cancer. Clin Cancer Res. 2005 Sep 15;11(18):6650–6.

24. Imamura Y, Lochhead P, Yamauchi M, Kuchiba A, Qian ZR, Liao X, et al. Analyses of clinicopathological, molecular, and prognostic associations of KRAS codon 61 and codon 146 mutations in colorectal cancer: cohort study and literature review. Mol Cancer. 2014 Dec;13(1):135.

25. Samowitz WS, Sweeney C, Herrick J, Albertsen H, Levin TR, Murtaugh MA, et al. Poor Survival Associated with the *BRAF* V600E Mutation in Microsatellite-Stable Colon Cancers. Cancer Res. 2005 Jul 15;65(14):6063–9.

26. Ogino S, Kawasaki T, Kirkner GJ, Kraft P, Loda M, Fuchs CS. Evaluation of Markers for CpG Island Methylator Phenotype (CIMP) in Colorectal Cancer by a Large Population-Based Sample. J Mol Diagn. 2007 Jul;9(3):305–14.

27. English DR, Young JP, Simpson JA, Jenkins MA, Southey MC, Walsh MD, et al. Ethnicity and Risk for Colorectal Cancers Showing Somatic *BRAF* V600E Mutation or CpG Island Methylator Phenotype. Cancer Epidemiol Biomarkers Prev. 2008 Jul 1;17(7):1774–80.

28. Eads CA. MethyLight: a high-throughput assay to measure DNA methylation. Nucleic Acids Res. 2000 Apr 15;28(8):32e–0.

29. Warth A, Kloor M, Schirmacher P, Bläker H. Genetics and epigenetics of small bowel adenocarcinoma: the interactions of CIN, MSI, and CIMP. Mod Pathol. 2011 Apr;24(4):564–70.

30. Park SJ, Rashid A, Lee JH, Kim SG, Hamilton SR, Wu TT. Frequent CpG Island Methylation in Serrated Adenomas of the Colorectum. 2003;162(3).

31. Rashid A, Shen L, Morris JS, Issa JPJ, Hamilton SR. CpG Island Methylation in Colorectal Adenomas. Am J Pathol. 2001 Sep;159(3):1129–35.
